# Supplementary material for: Women and ARV-based prevention: opportunities and challenges
Source: J Int AIDS Soc. 2014 Sep 8;17(3Suppl 2):19419. doi: 10.7448/IAS.17.3.19419 (PMC4165039; doi:10.7448/IAS.17.3.19419)
Supplement: Related texts

# Sub article: Women and ARV-based HIV prevention – challenges and opportunities

**DOI:** 10.7448/IAS.17.3.19356

**Published:** 2014-09-08

**Authors:** Cynthia W Geary, Elizabeth A Bukusi

**Affiliations:** 1 FHI 360, Durham, NC, USA; 2 Centre for Microbiology Research, Kenya Medical Research Institute, Nairobi, Kenya; 3 Departments of Obstetrics and Gynecology, and Global health, University of Washington, Seattle,WA, USA; 4 Department of Obstetrics and Gynecology Aga Khan University, Nairobi, Kenya; 5 Department of Obstetrics and Gynecology, Nairobi University, Nairobi, Kenya; 6 Department of Obstetrics, Gynecology and Reproductive Sciences, University of California San Francisco, USA

**Keywords:** women, gender, HIV prevention, ARVs, microbicides, PrEP, risk compensation

## Abstract

ARV-based HIV prevention methods available in pill, gel or ring formulations (broadly referred to as microbicides) offer the possibility of protection against HIV for women who find it difficult because they cannot ask their partners to use condoms or even refuse sex. Partial efficacy of ARV-based medications has been demonstrated in a number of clinical trials around the world among various populations, building the evidence that ARV-based technologies will contribute to reducing the AIDS epidemic worldwide. Disappointing results, however, from two trials in sub-Saharan Africa, where poor adherence contributed to study closure due to futility, have raised questions about whether women at the centre of the epidemic are able to effectively use products that require routine use. Also, there are fears by some of risk compensation by decreased condom use because of the availability of microbicides when only partial efficacy has been demonstrated in microbicide trials to date. Of note, sub-analyses of biologic measures of adherence in trials where this was possible have shown a strong correlation between good adherence and efficacy, reinforcing the necessity of good adherence. Research conducted in conjunction with clinical trials and post-trials in advance of possible rollout of ARV-based products have examined social and cultural factors, gender-related and otherwise, influencing adherence and other aspects of women's use of products. These include HIV stigma, women's perception of risk, partner and community influences and the differing needs of women in various stages of life and in different circumstances. It is the purpose of this supplement to give voice to the needs of women who can benefit from woman-initiated methods by presenting research results and commentary to contribute to the global conversation about optimizing women's experience with ARV-based prevention.

## References

[1] Heise L, Ellsberg M, Gottemoeller M (1999). Ending violence against women. Population reports, Series L., No. 11.

[2] Abdool Karim Q, Abdool Karim SS, Frohlich JA, Grobler AC, Baxter C, Mansoor LE (2010). Effectiveness and safety of tenofovir gel, an antiretroviral microbicide for the prevention of HIV infection in women. Science.

[3] Thigpen MC, Kebaabetswe PM, Paxton LA, Smith DK, Rose CE, Segolodi TM (2012). Antiretroviral pre-exposure prophylaxis for heterosexual HIV transmission in Botswana. N Eng J Med.

[4] Grant RM, Lama JR, Anderson PL, McMahan V, Liu AY, Vargas L (2012). Pre-exposure chemoprophylaxis for HIV prevention in heterosexual men and women. N Eng J Med.

[5] Baeten JM, Donnell D, Ndase P, Mugo NR, Campbell JD, Wangisi J (2012). Antiretroviral prophylaxis for HIV prevention in heterosexual men and women. N Engl J Med.

[6] Choopanya K, Martin M, Suntharasamai P, Sangkum U, Mock PA, Leethochawalit M (2013). Antiretroviral prophylaxis for HIV infection in injecting drug users in Bangkok, Thailand (The Bangkok Tenofovir Study): a randomized, double-blind, placebo-controlled phase 3 trial. Lancet.

[7] Joint United Nations Programme on HIV/AIDS (UNAIDS) (2013). Global report: UNAIDS report on the global AIDS epidemic [Internet].

[8] Van Damme L, Corneli A, Ahmed K, Agot K, Lombaard J, Kapiga S (2012). Pre-exposure prophylaxis for HIV infection among African women. N Engl J Med.

[9] Marrazzo J, Ramjee G, Nair G, Palance T, Mkhize B, Nakabiito C 20th Conference on Retroviruses and Opportunistic Infections. Pre-exposure prophylaxis for HIV in women: daily oral tenofovir, oral tenofoviremtricitabine, or vaginal tenofovir gel in the VOICE Study (MTN 003).

[10] Corneli A, Deese J, Wang, Meng MS, Taylor D, Ahmed K (2014). Adherence patterns and factors associated with adherence to a daily oral study product for pre-exposure prophylaxis. J Acquir Immune Defic Syndr.

[11] Van der Straten A, Stadler J, Luecke E, Laborde N, Hartmann M, Montgomery E (2014). Perspectives on use of oral and vaginal antiretrovirals for 1 HIV prevention: the VOICE-C study in Johannesburg, South Africa. J Int AIDS Soc.

[12] Corneli A, Perry B, McKenna K, Agot K, Ahmed K, Malamatsho F Participants’ explanations for non-adherence in the FEM-PrEP clinical trial.

[13] Corneli A, McKenna K, Headley J, Ahmed K, Odhiambo J, Skhosana J (2014). A descriptive analysis of perceptions of HIV risk and worry about acquiring HIV among FEM-PrEP participants who seroconverted in Kenya and South Africa. J Int AIDS Soc.

[14] Eaton L, Kalichman S (2007). Risk compensation in HIV prevention. Curr HIV/AIDS Rep.

[15] Mastro T, Sista N, Abdool-Karim Q (2014). ARV-based HIV prevention for women – where are we in 2014?. J Int AIDS Soc.

[16] Lanham M, Wilcher R, Montgomery E, Pool R, Schuler S, Lenzi R (2014). Engaging male partners in women's microbicide use: evidence from clinical trials and implications for future research and microbicide introduction. J Int AIDS Soc.

[17] The DHS Program STAT Compiler (c2012). http://www.statcompiler.com.

[18] AVAC (2013). AVAC Report 2013: research and reality.

[19] MacQueen K, Tolley E, Owen D, Amico R, Morrow K, Moench T (2014). An interdisciplinary framework for measuring and supporting adherence in HIV prevention trials of ARV-based vaginal rings. J Int AIDS Soc.

[20] Tolley E, Kaaya S, Kaale A, Minja A, Bangapi D, Kalungura H (2014). Comparing patterns of sexual risk among adolescent and young women in a mixed methods study in Tanzania: implications for adolescent participation in HIV prevention trials. J Int AIDS Soc.

[21] Sidibe S, Pack A, Tolley E, Ryan E, Mackenzie C, Bockh E (2014). Communicating about microbicides with women in mind: Tailoring messages for specific audiences. J Int AIDS Soc.

[22] Mack N, Evens E, Tolley E, Brelsford K, McKenzie C, Milford C (2014). The importance of choice in the rollout of ARV-based prevention to user groups in Kenya and South Africa: a qualitative study. J Int AIDS Soc.

[23] Woodsong C, Mustambi J, Ntshele S, Modikoe P (2014). Community and research collaboration for development of materials to inform microbicide trial participants in Africa. J Int AIDS Soc.

[24] Lin A, Barnhart M, Breger T, Harris E, Kim A, Vansgaard C (2014). Learning from the private sector: towards a keener understanding of the end-user for microbicide introduction planning. J Int AIDS Soc.

[25] Lusti-Narasimhan M, Khosla R, Baggaley R, Temmerman M, McGrory E, Farley T (2014). WHO guidance grounded in a comprehensive approach to sexual and reproductive health and human rights: topical pre-exposure prophylaxis. J Int AIDS Soc.

# Sub article: Perspectives on use of oral and vaginal antiretrovirals for HIV prevention: the VOICE-C qualitative study in Johannesburg, South Africa

**DOI:** 10.7448/IAS.17.3.19146

**Published:** 2014-09-08

**Authors:** Ariane van der Straten, Jonathan Stadler, Ellen Luecke, Nicole Laborde, Miriam Hartmann, Elizabeth T Montgomery, on behalf of the VOICE-C Study Team

**Affiliations:** 1 Women's Global Health Imperative, RTI International San Francisco, CA, USA; 2 Department of Medicine, Center for AIDS Prevention Studies, University of California San Francisco San Francisco, CA, USA; 3 Wits Reproductive Health and HIV Institute, Faculty of Health Sciences, University of the Witwatersrand, Johannesburg, South Africa

**Keywords:** HIV prevention, antiretroviral, pre-exposure prophylaxis, microbicides, adherence, South Africa, qualitative methods, HIV stigma

## Abstract

**Introduction:**

Antiretroviral (ARV)-based pre-exposure prophylaxis (PrEP) is a promising new HIV prevention strategy. However, variable levels of adherence have yielded mixed results across several PrEP trials and populations. It is not clear how taking ARV – traditionally used for HIV treatment – is perceived and how that perception may affect the use of these products as preventives. We explored the views and experiences of VOICE participants, their male partners and community members regarding the use of ARV as PrEP in the VOICE trial and the implications of these shared meanings for adherence.

**Methods:**

VOICE-C was a qualitative ancillary study conducted at the Johannesburg site of VOICE, a multisite, double-blind, placebo-controlled randomised trial testing tenofovir gel, oral tenofovir and oral Truvada^®^ for HIV PrEP. We interviewed 102 randomly selected female VOICE participants, 22 male partners and 40 community members through in-depth interviews, serial ethnography, or focus group discussions. All interviews were audiotaped, transcribed, translated and coded thematically for analysis.

**Results:**

The concept of ARV for prevention was understood to varying degrees across all study groups. A majority of VOICE participants understood that the products contained ARV, more so for the tablets than for the gel. Although participants knew they were HIV negative, ARV was associated with illness. Male partners and community members echoed these sentiments, highlighting confusion between treatment and prevention. Concerned that they would be mistakenly identified as HIV positive, VOICE participants often concealed use of or hid their study products. This occasionally led to relationship conflicts or early trial termination. HIV stigma and its association with ARV, especially the tablets, was articulated in rumour and gossip in the community, the workplace and the household. Although ARV were recognised as potent and beneficial medications, transforming the AIDS body from sickness to health, they were regarded as potentially harmful for those uninfected.

**Conclusions:**

VOICE participants and others in the trial community struggled to conceptualise the idea of using ARV for prevention. This possibly influenced willingness to adopt ARV-based prevention in the VOICE clinical trial. Greater investments should be made to increase community understanding of ARV for prevention and to mitigate pervasive HIV stigma.

## Introduction

Recently, oral antiretroviral (ARV)-based pre-exposure prophylaxis (PrEP) was established as an effective new HIV prevention strategy. Nevertheless, adequate protection hinges on correct and consistent product use. Adherence challenges have emerged as a key reason for divergent results across PrEP studies, in different locations and with different populations [1–9]. Further, across vaginal and oral routes, daily, intermittent, or coitally related dosing regimens have proved difficult to execute [1, 3, 6, 7, 10–15].

Tenofovir was originally designed for HIV treatment and is part of first-line ARV therapy regimens in Africa. Treatment adherence has been hailed as a success in reducing mortality and morbidity amongst people living with HIV [16–19]. However, public secrecy of HIV status and concealment of AIDS suffering and stigma continue to undermine treatment adherence to ARV; patients may try to avoid revealing their HIV status by concealing their medication, compromising adequate use [20–22].

Similar issues arise with regard to using ARV to prevent HIV acquisition because it may generate social stigma, particularly in contexts where this prevention approach is unfamiliar [23]. In Kenya, stigma was reported to negatively affect participants’ ability to adhere to an oral PrEP regimen [11]. Similarly, Thai participants in another oral PrEP trial feared being mistakenly identified as HIV positive and experienced stigma and relationship stress [14]. Oral PrEP tablets are readily identifiable as ARV and thus attract unwanted public scrutiny [11, 14]. Consequently, participants in prevention studies conceal their tablets because of fears of negative reactions [11, 23, 24], avoid carrying their study products with them [14], or lie about the reason for taking the tablets [11].

The VOICE trial evaluated daily oral and topical tenofovir-based HIV PrEP among women in South Africa, Uganda and Zimbabwe. In September 2011, the oral tenofovir arm (TDF; referred to as “tenofovir” by the study team) was discontinued for futility. In November 2011, a similar determination was made for the tenofovir (TFV) gel arm. The emtricitabine-tenofovir (referred to as “Truvada” by the study team) and oral placebo arms continued until planned exit in August 2012 [6]. All three products tested were found safe; however, none reduced the risk of HIV-1 acquisition, given widespread low product use, based on post-trial pharmacokinetic drug testing in biological specimens [6]. Similar to VOICE, FemPrEP, a phase III trial of oral Truvada^®^ tablets, was unable to demonstrate effectiveness because of low adherence [3].

We recently reported findings from a qualitative study, VOICE-C, which examined the contextual factors influencing daily use of vaginal gel and oral tablets for HIV prevention in the VOICE trial. We found that few participants acknowledged non-use and that social relations within the household and the community shaped women's experiences of trial participation and the trial products [25]. In this paper, we explore further the views and experiences of participants, specifically in relation to the use of ARV as PrEP in the VOICE trial and the implications of these shared meanings for product adherence.

## Methods

VOICE-C was a qualitative exploratory ancillary study implemented between July 2010 and August 2012, concurrent with the VOICE trial. As part of the VOICE trial's procedures (detailed in [6]), when the active products were referred to, staff used a pharmaceutical name (tenofovir or Truvada), or described them as having “medicine.” A number of study materials, as well as education and counselling procedures regularly provided during the trial explicitly stated that the active ingredients in the investigational products contained ARV.

VOICE-C was conducted at one of the 15 VOICE sites, the Wits Reproductive Health Institute (Wits RHI), in Johannesburg, South Africa. The research clinic is located in Hillbrow, a low-income, densely populated, inner-city suburb of Johannesburg. VOICE participants were recruited from Hillbrow, neighbouring suburbs and more distant townships. The VOICE-C study has been previously described [25]. Briefly, VOICE-C enrolled four groups of people: VOICE participants, their male partners, community advisory board (CAB) members and other community stakeholders. The VOICE participants were randomly preselected and randomly assigned to one in-depth interview (IDI; *N*=41) or serial ethnographic interviews (EI; *N*=21) conducted during the course of the VOICE study, or one exit focus group discussion (FGD; *N*=40). Male partners of VOICE participants were recruited to participate in IDI (*N*=14) conducted during the course of the study or an exit FGD (*N*=8) as previously described [25]. Finally, a convenience sample of 40 community members joined FGDs. These included 17 CAB members who participated in up to four serial FGDs and 23 community stakeholders who took part in one of three FGDs, both of which were conducted during VOICE implementation. Each of these latter FGDs comprised individuals who lived or worked in Hillbrow and were homogeneous in terms of their professional affiliation: 1) community-based organisations involved in HIV/AIDS; 2) local media, including community newspapers and radio stations; and 3) a local neighbourhood improvement programme. Out of all participants screened, one female participant, three male partners and one community stakeholder refused participation.

### Procedures

IDIs and FGDs were conducted at the research site. The EIs (a longitudinal series of 2–4 interviews) took place at the interviewees’ home or a private location of her choice. All IDIs, EIs and FGDs were conducted by trained gender-matched research staff in the participants’ languages of choice; they were audio-recorded, transcribed and translated into English (when conducted in another language). The interviews included a short demographic questionnaire. Selected survey data were collected during participants’ VOICE clinic visits.

All English language transcripts were coded in Nvivo (version 9.0, Burlington, MA) by the analysis team, which included members of the data coordinating centre and site staff, using a codebook that followed a socio-ecological framework (SEF) (Figure 1) [25]. Coded data were concatenated into coding reports by thematic area (e.g., ARV, PROTECT, SIDE EFFECT, PREFERENCE) and by SEF level codes (e.g. HOUSEHOLD, CLINIC, COMMUNITY) and then summarised into memos. Memos from each area were further analysed to reveal patterns or themes related to the use of ARV in the trial. The study team was blinded to study arm assignments of VOICE participants until the last analytical step, when participants’ perceived product assignment was assessed.

**Figure 1:** Socio-ecological framework of factors affecting perceptions about ARV for pre-exposure prophylaxis (PrEP), by levels of influence, among VOICE-C participants. Note: Some of the factors may operate at multiple levels of influence, but are only presented at the highest level at which they operate and are not repeated in lower levels. The organisational level of influence in this framework is focused on the clinical trial setting.

All participants provided written informed consent prior to participation. The study protocol was approved by the Institutional Review Boards at RTI International and the University of the Witwatersrand, and overseen by the regulatory infrastructure of the U.S. National Institutes of Health and the Microbicides Trial Network.

## Results

Of the 102 ethnically diverse VOICE female participants who joined VOICE-C, the mean age was 27 years, 68% had completed secondary school and 96% had a main male partner although only 22% were married. Women completed a structured survey at VOICE trial exit asking about their perceived product assignment. Although 51% were actually assigned to an active treatment arm, 62% perceived having received an active product (Table 1). The characteristics of the other groups in VOICE-C, the male partners (*N*=22) and the community members (*N*=40), are also presented in Table 1.

**Table 1:** Demographic characteristics of participants by VOICE-C study groups

	VOICE participants	Male partners	Community members
At time of VOICE-C (first) interview	*N*=102 (%)	*N*=22 (%)	*N*=40 (%)
Median age (mean, range)	26.8 (19–40)	31.4 (22–45)	38.5 (20–60)
Currently married	22 (22%)	9 (41%)	17 (43%)
Has current primary sex partner	98 (96%)	22 (100%)	
Length of relationship in years (mean, range)	5.5 (0.1–25)	5.9 (1–10)	
Currently living with primary sex partner	44 (43%)	16 (72%)	
Completed secondary school or more	69 (68%)	14 (64%)	31 (78%)
Does not earn an income	44 (43%)	4 (18%)	13 (33%)
Ethnic group			
Zulu	27 (26%)	4 (18%)	5 (13%)
Xhosa	13 (13%)	3 (14%)	5 (13%)
Sotho	19 (19%)	4 (18%)	10 (25%)
Ndebele	26 (25%)	5 (23%)	1 (3%)
Other^a^	17 (17%)	6 (27%)	19 (48%)
Religion			
Christian	94 (92%)	17 (77%)	36 (90%)
Muslim	0 (0%)	2 (9%)	0 (0%)
Other/none	8 (8%)	3 (14%)	4 (10%)
History of involvement with HIV research/work	43 (42%)	5 (23%)	31 (78%)
Type of interviews received^b^			
In-depth interview	41 (40%)	14 (64%)	
Ethnographic interview	21 (21%)		
Focus group discussion	40 (39%)	8 (36%)	40 (100%)
(Initial) interview conducted prior to first DSMB	44 (43%)	9 (41%)	28 (70%)
Treatment arm assignment			
Truvada^®^	22 (22%)		
Viread^®^	18 (18%)		
Oral placebo	22 (22%)		
Tenofovir (TFV) gel	21 (21%)		
Placebo gel	19 (19%)		
Perceived assignment to active product (*N*=83)^c^	53 (64%)		

a Other ethnic groups: KALANGA 1, KHALANGA 1, NYANJA 1, SHONA 2, SWATI 1, SWAZI 1, TSONGA 3, TSWANA 4, VENDA 3

b the procedural changes because of VOICE Data Safety and Monitoring Board (DSMB) futility recommendations contributed to several women randomly preselected for VOICE-C participation receiving an earlier interview than anticipated, being reallocated to a different interview modality, or to stopping their serial ethnographic interviews early

c perceived product assignment was assessed at the VOICE clinic exit visit, TDF participants (*N*=18) were not asked this question, because of early stopping of this arm following the first DSMB futility recommendation [26]. IQR=inter quartile range.

### ARV for treatment versus prevention

VOICE-C participants had varying levels of awareness that active trial products contained ARV, although there was greater cognizance that the tablets contained ARV than the vaginal gel. Among gel participants, about half were unaware that the active gel contained ARV. Participants were initially surprised when they learnt that the trial was testing ARV as a means of preventing HIV. Lily (all names are pseudonyms) recounts her feelings upon such learning:

I didn't have a problem with the tablets but I only got scared when I heard that these tablets are ARV, I got scared to think that we are now going to take tablets used by people who are sick when we weren't sick at all. (Lily, age 40, Tablet FGD)

In some cases, women speculated that the products only contained “some of the same components as ARV for treatment.” Others such as Limpho initially misunderstood the information provided by staff at the trial clinic.

(…) According to me, they said they are doing a research to find out if they [ARV] work, you see, but then in my mind I thought, okay, maybe it will help protect people that have AIDS. I had not understood correctly. (Limpho, age 21, Tablet EI)

Otillia used the acronym ART (antiretroviral therapy) to differentiate the study drugs from ARV, in a conversation with her friend.

I showed her and she said “Yoh! They look like ARV,” and I said “Yes they look like this but at the clinic they said these study tablets are ART they are not the same as ARV,” you see? (Otillia, age 26, Tablet EI)

When the interviewer prompted Otillia about the difference between ARV and ART, she first said they are different “because of what they do in the body,” but ended up confused with her own explanations.

However, several participants reacted negatively. Thoko (Gel, age 25, EI) stated that she would not use the gel if it contained ARV and would drop out of the study. According to her, “ARV are for sick people” and not for prevention. She did not understand the reasons to give ARV to healthy women.

The uncertainty and confusion expressed by many participants like Thoko stems partly from difficulties in distinguishing between treatment and prevention, particularly relating to using ARV. IsiZulu and SeSotho speakers used the words *ukuvikela* or *sirelela*, respectively, for prevention or treatment, while others used the English words *treat* and *prevent* interchangeably. With respect to ARV taken for treatment, participants also used the English word *cure*. The conflation of treatment and prevention potentially creates difficulties in conceptualising the idea of PrEP, particularly when the prevention technologies use the same ARV drugs, usually taken for treatment. Indeed, in some cases, HIV-positive family members stole participants’ tablets, presumably to treat their illness. Palesa's (Tablet, age 21, FGD) mother acquired a sexually transmitted disease and, worried that she may also be infected with HIV, started using her daughter's trial tablets. Tshepiso's (Tablet, age 27, FGD) aunt was HIV positive and stole her tablets when her own ARV ran out.

Male partners were not enrolled in the VOICE trial and unsurprisingly did not have a clear understanding of the trial in general or of using ARV for prevention in particular. Some questioned the legitimacy of the trial when they learnt that ARV were tested: for example, they asked why HIV-negative women received ARV and suspected that their female partners may be HIV positive because they used ARV.

Other male partners were more concerned about the health risks associated with ARV [27]. One man said his partner may acquire HIV from taking ARV and that this would affect their future progeny. Although he insisted that his partner cease taking the trial tablets and end trial participation, she remained. Others were more sympathetic, although they too were poorly informed. For example one man thought the study was meant to detect ARV in different biological specimens and trusted that medical researchers would not give his partner unsafe drugs. Despite the negative associations of the tablets, male partners appeared to prefer these to the gel, because of the unwanted vaginal wetness that could occur after inserting gels.

The CAB and other stakeholders articulated their difficulties in communicating the concept and providing the correct information about ARV for prevention in the community. They also expressed concerns with the side effects of PrEP, and that women in VOICE would be identified as HIV positive. Nevertheless, they said that PrEP would be acceptable if it was found to be efficacious in preventing HIV acquisition.

### PrEP and HIV stigma

All categories of participant (VOICE participants, male partners and community members) recounted overarching apprehensions with HIV stigma in their community. VOICE participants sometimes referred to HIV/AIDS as “that disease” and “the flu” and one remarked that “being HIV positive is seen as being in style” (Mandisa, age 25, Gel IDI). This appears to downplay or trivialise the seriousness of the disease, particularly in the era of effective treatment. As one male partner stated:

People they still die before knowing about AIDS and people are still dying, they are still going to die, so what's so special about this AIDS, you understand? That's what they think. If I get HIV/AIDS I will just go and get treatment and live longer than those people who are not even infected, so why worry. (Antonio, male partner FGD, Gel)

Yet, it also signifies a widespread view of the ubiquity of the HIV/AIDS epidemic and the futility or hopelessness in trying to prevent its spread.

Local constructions of HIV such as these had implications for women who used ARV to prevent HIV and feared being mistakenly identified as HIV positive and suffering discrimination and social isolation. Trial participants recognised this and went to great lengths to disguise their involvement in VOICE. They were secretive and hid their products from household members, co-workers and friends to avoid accidental disclosure, although this made taking the products more difficult. Keneoe (Gel, age 21, EI) hid her gels in the ceiling to avoid detection and Tale applied her gel in the toilet, the only private place in her home.

At times there will be a lot of people in the house and you know how it is like in South Africa. The bedroom serves a multi-purpose task of being a sitting room at the same time. Hence the bathroom is always the safer option where I would be able to do my business [insert gel] without disturbance from anyone. (Tale, age 24, Gel IDI)

For trial participants, explaining the reasons for taking the tablets was often too burdensome because it was easier to be secretive. “I hated that thing of having someone ask me because I would have to explain everything,” said Nomsa (Tablet, age 26, FGD).

Conscious of the possibility of unkind gossip and rumour, and of discriminatory action being taken against her, Naledi remarked:

The area that I live in has a bad influence because they like to gossip about people. If they could see these tablets, obviously they are going to gossip (…) if they could see me taking the tablets they will say that I have AIDS. They would not listen to my explanation about these tablets. (Naledi, age 23, Tablet IDI)

For some, the risk of being labelled HIV positive outweighed the benefits of participating in the trial. Gladys experienced discrimination first hand when her flatmates suspected she was HIV positive:

There is that stigma, discrimination and stigmatisation of those people who have got AIDS. So, they [flatmates] started to change the way in which they were living, you know. When we have drunk with this cup, they will just not touch it – they will start doing those things. (Gladys, age 33, Tablet EI)

This had implications for adherence and participation in the trial. Nogoli (Gel, age 31, FGD) recounted a study participant who withdrew from the trial because her mother-in-law pressured her to stop taking ARV by saying “you cannot take ARV when you are not sick. They [researchers] only want to make you sick.”

However, there are also several examples of women who managed these outsiders’ perceptions by referring to the VOICE trial fact sheets, undergoing HIV testing at a local clinic with suspecting relatives, or simply discounting the disparaging remarks made of them as a result of ignorance; such is the case for Thuli's cousin:

She says I will catch the flu [HIV]. I tried to give her the papers [fact sheets] to read and see what this thing [VOICE] is all about is. She is ignorant and she pretends that she knows everything. […] She said that if I continue testing I will be infected. (Thuli, age 24, Gel FGD)

Overall, VOICE participants assigned to gel preferred it to the tablet, while tablet users were divided about their preference for tablets or gel. The association of study products with ARV was seldom specifically mentioned when stating product preference. However, six women did invoke this as a reason to prefer gel over tablets: gel did not carry the HIV stigma associated with the tablets, and is less recognisable, as people don't know what the gel is for.

### ARV potency and safety

Participants’ ambivalence towards ARV was expressed several ways: on the one hand they recognised that ARV are extremely potent, transforming the AIDS body from sickness to health, and were therefore highly beneficial.

ARV are taken by positive people, they get fat isn't it? That thing encourages me because I live with positive people in my community. I see how the ARVs are working on them. So that thing encourages me to want to take them. (Javas, age 30, Tablet FGD)

This awareness of the power of ARV influenced participants’ accounts of the side effects. For example, akin to the dramatic physical transformations of HIV-infected people on treatment, many tablet participants reported weight gain, which they attributed to taking the tablets (although there was no evidence of this in the clinical data). Others regarded the side effects as confirmation that they had been randomised to the active arms of the study products, whereas those who did not experience any side effects tended to think they had been randomised to placebo. Yet, the potency of ARV was also regarded as a source of potential harm for those uninfected.

I did have my concerns with these things since well it is part of the ARVs and you know what the ARVs does in your body[…] Even today I know that ARVs can react badly in your body so this [gel] in the long run, will it not affect me? (Abri, age 31, Gel IDI)

There also was some speculation amongst community members and male partners that taking ARV could cause one to seroconvert. Stories that circulated in the media and amongst trial participants about ARV sold and stolen to mix in with a heroin or crystal methamphetamine-based drug called *Nyaope* (also known as *Whoonga* or *Wunga*) added to the popular conceptualisation of the dangerous potency of ARV [28].

I do not feel safe since there are people in the community who smoke ARVs to get high. Thugs mix ARVs with some other things […] I heard that the [thugs] sell ARVs and they get good profit. (Jewel, age 24, Tablet IDI)

These rumours added another dimension to the stories of ARV potency and danger – that of physical risk – associated with carrying ARV in public places.

## Discussion

This qualitative study conducted in Johannesburg, South Africa, explored local perspectives on using ARV as PrEP to prevent HIV in the VOICE trial, which was unable to demonstrate effectiveness because of low product adherence. Focusing our analysis on the social meanings of ARV, we found a variety of perspectives amongst trial participants, their male partners and community members. Recognition that the study products contained ARV was not unanimous and women's understandings of PrEP were mediated through confusion surrounding the use of ARV as a prevention method, versus treatment. Understandings of health that were articulated here were often incommensurate with biomedical models, revealing a tendency to conflate prevention and treatment. Yet, the very fact that many participants knew ARV are used for treatment, inferred a known distinction between prevention and treatment. This apparent contradiction may stem from the fact that ARV for prevention was difficult to grasp and reconcile with the more familiar treatment model. In other words participants had trouble distinguishing ARV for prevention from treatment and yet were aware that it wasn't the same thing.

Given high HIV prevalence and stigmatisation of the disease, taking ARV for prevention became a marker of HIV infection. Despite a few participants suggesting that HIV seropositivity may be normalized or even trendy, the strong association of ARV with a positive status threatened to engender HIV stigma and secrecy around the use of the trial products and potentially challenged product adherence and trial participation. Although trial participants had worries about side effects, concerns about the social impact of being identified as HIV positive appeared to be greater. The stigma of HIV is based not only on sexual morality, but also on the perception of HIV as an incurable illness [29] and has contributed towards the secrecy and concealment of serostatus [30]. The threat of a “spoilt identity” [31] from being mistakenly identified as HIV positive influenced participants’ willingness to use ARV for prevention, especially in the context of a placebo-controlled trial where the actual benefit of using these products was uncertain.

The novelty of PrEP for HIV prevention combined with pervasive HIV stigma [32, 33] led to anxieties about being identified as HIV positive, more so among tablet than gel participants. Although ARV were recognised as having a positive transformative effect on AIDS sufferers [34], their potency was also regarded as dangerous, particularly when used by individuals who were not HIV positive.

As growing numbers of clinical trials are conducted in settings where there is limited experience of biomedical research practices, the need for understanding local constructs of disease and responses to novel medical technologies increases. How are these new applications of drugs such as ARV understood and negotiated in contexts that are characterised by stigma and blame, and what are the implications for wider access to PrEP in mainstream public health care? In this study we found that ARV for prevention was interpreted with ambivalence. Although regarded as life-saving medications, ARV were also seen as potentially harmful; hence the belief, albeit not widely shared, that ARV taken by uninfected individuals could lead to acquiring HIV, or the association of ARV with illegal narcotics and criminality. The result of this ambivalence created uncertainty and doubt, potentially undermining women's motivation to adhere to the trial products.

Other studies of HIV prevention interventions have reported challenges with local understandings of biomedical models [35]. A potentially fruitful area of social research may be how biomedical researchers can engage with local understandings of prevention, treatment and cure to improve correct usage and sustained adherence to ARV for PrEP in southern Africa and elsewhere.

Two seemingly opposite undercurrents of opinions emerged here: one linked to HIV stigma and the other to ubiquity and trivialisation of HIV/AIDS. Although South Africa is a country most heavily burdened by the AIDS pandemic, the relatively recent universal access to ART has changed the landscape, turning a terminal disease into a chronic, manageable illness. Simultaneously, stigma and discrimination against those infected persist and prevent HIV-positive individuals from accessing the treatment and care they need [36]. Although these perceptions appear to go in different directions they may both work against acceptance of ARV for PrEP: one because prevention is no longer seen as important and the other because misattribution of seropositivity may discourage those at risk to access or use ARV for PrEP.

There are several limitations to this study: VOICE was implemented in three countries and across 15 sites, VOICE C was conducted at only one site, in Johannesburg, thus our findings may not reflect the perspectives of participants at other sites. Further, in South Africa, ARVs have been the centre of controversy, particularly under the leadership of former President Thabo Mbeki and his Health Minister, Manto Tshabalala-Msimang, who publicized the alleged toxic effects of ARV, and questioned the orthodox view of HIV causing AIDS [37]. This may have uniquely shaped VOICE-C participants’ experiences regarding both mistrust of research and ARV for prevention. Given South Africa's central place in the global AIDS pandemic, this warrants further research. Notably, studies in South Africa and other countries corroborate our findings that taking “AIDS pills” or ARVs for prevention generates concerns and may be associated with product non-use [11, 23, 24, 38].

In summary, socio-contextual factors influenced willingness to adopt ARV-based prevention, in the context of a large clinical trial. Greater investments should be made to increase community-wide understanding of ARV for prevention and to mitigate pervasive HIV stigma.

## References

[1] Grant RM, Lama JR, Anderson PL, McMahan V, Liu AY, Vargas L (2010). Pre-exposure chemoprophylaxis for HIV prevention in men who have sex with men. N Engl J Med.

[2] Baeten JM, Donnell D, Ndase P, Mugo NR, Campbell JD, Wangisi J (2012). Antiretroviral prophylaxis for HIV prevention in heterosexual men and women. N Engl J Med.

[3] Van Damme L, Corneli A, Ahmed K, Agot K, Lombaard J, Kapiga S (2012). Pre-exposure prophylaxis for HIV infection among African women. N Engl J Med.

[4] Thigpen MC, Kebaabetswe PM, Paxton LA, Smith DK, Rose CE, Segolodi TM (2012). Antiretroviral pre-exposure prophylaxis for heterosexual HIV transmission in Botswana. N Engl J Med.

[5] Choopanya K, Martin M, Suntharasamai P, Sangkum U, Mock PA, Leethochawalit M (2013). Antiretroviral prophylaxis for HIV infection in injecting drug users in Bangkok, Thailand (the Bangkok Tenofovir Study): a randomised, double-blind, placebo-controlled phase 3 trial. Lancet.

[6] Marrazzo J, Ramjee G, Nair G, Palanee T, Mkhize B, Nakabiito C (2013). Pre-exposure prophylaxis for HIV in women: daily oral tenofovir, oral tenofoviremtricitabine, or vaginal tenofovir gel in the VOICE Study (MTN 003). CROI.

[7] van der Straten A, Van Damme L, Haberer JE, Bangsberg DR (2012). Unraveling the divergent results of pre-exposure prophylaxis trials for HIV prevention. AIDS.

[8] Baeten J, Celum C (2012). Oral antiretroviral chemoprophylaxis: current status. Curr Opin HIV AIDS.

[9] Baeten J, Celum C (2013). Systemic and topical drugs for the prevention of HIV infection: antiretroviral pre-exposure prophylaxis. Annu Rev Med.

[10] Abdool Karim Q, Abdool Karim SS, Frohlich JA, Grobler AC, Baxter C, Mansoor LE (2010). Effectiveness and safety of tenofovir gel, an antiretroviral microbicide, for the prevention of HIV infection in women. Science.

[11] Van der Elst EM, Mbogua J, Operario D, Mutua G, Kuo C, Mugo P (2012). High acceptability of HIV pre-exposure prophylaxis but challenges in adherence and use: qualitative insights from a phase I trial of intermittent and daily PrEP in at-risk populations in Kenya. AIDS Behav.

[12] Minnis AM, Gandham S, Richardson BA, Guddera V, Chen B, Salata R (2013). Adherence and acceptability in MTN 001: a randomized cross-over trial of daily oral and topical tenofovir for HIV prevention in women. AIDS Behav.

[13] Muchomba FM, Gearing RE, Simoni JM, El-Bassel N (2012). State of the science of adherence in pre-exposure prophylaxis and microbicide trials. J Acquir Immune Defic Syndr.

[14] Tangmunkongvorakul A, Chariyalertsak S, Amico KR, Saokhieo P, Wannalak V, Sangangamsakun T (2013). Facilitators and barriers to medication adherence in an HIV prevention study among men who have sex with men in the iPrEx study in Chiang Mai, Thailand. AIDS Care.

[15] Amico KR, Liu A, McMahan V, Anderson PL, Lama JR, Guanira J Adherence indicators and pre-exposure prophylaxis (PrEP) drug levels in the iPrEx study.

[16] Ammassari A, Trotta MP, Shalev N, Marconi P, Antinori A (2012). Beyond virological suppression: the role of adherence in the late HAART era. Antiviral Ther.

[17] Munakata J, Benner JS, Becker S, Dezii CM, Hazard EH, Tierce JC (2006). Clinical and economic outcomes of nonadherence to highly active antiretroviral therapy in patients with human immunodeficiency virus. Med Care.

[18] Paterson DL, Swindells S, Mohr J, Brester M, Vergis EN, Squier C (2000). Adherence to protease inhibitor therapy and outcomes in patients with HIV infection. Ann Intern Med.

[19] Gross R, Bilker WB, Friedman HM, Strom BL (2001). Effect of adherence to newly initiated antiretroviral therapy on plasma viral load. AIDS.

[20] Dlamini PS, Wantland D, Makoae LN, Chirwa M, Kohi TW, Greeff M (2009). HIV stigma and missed medications in HIV-positive people in five African countries. AIDS Patient Care STDS.

[21] Skhosana NL, Struthers H, Gray GE, McIntyre JA (2006). HIV disclosure and other factors that impact on adherence to antiretroviral therapy: the case of Soweto, South Africa. Afr J AIDS Res.

[22] Ware NC, Wyatt MA, Tugenberg T (2006). Social relationships, stigma and adherence to antiretroviral therapy for HIV/AIDS. AIDS Care.

[23] Ambia J, Agot K (2013). Barriers and facilitators of adherence in user- dependent HIV prevention trials, a systematic review. Int STD Res Rev.

[24] Koenig LJ, Lyles C, Smith DK (2013). Adherence to antiretroviral medications for HIV pre-exposure prophylaxis: lessons learned from trials and treatment studies. Am J Prev Med.

[25] van der Straten A, Stadler J, Montgomery E, Hartmann M, Magazi B, Mathebula F (2014). Women's experiences with oral and vaginal pre-exposure prophylaxis: the VOICE-C qualitative study in Johannesburg, South Africa. PLoS One.

[26] Microbicide trials network statement on decision to discontinue use of tenofovir gel and oral tablets in VOICE, a major HIV prevention study in women (2011). http://www.mtnstopshiv.org/node/3909.

[27] Montgomery E, Stadler J, Hartmann M, Magee C, Magazi B, Mathebula F (2013). Male partner roles and influence on women's use of HIV pre-exposure prophylaxis in Johannesburg. AIDS Impact; 2013 Sep 29–Oct 3.

[28] Grelotti DJ, Closson EF, Smit JA, Mabude Z, Matthews LT, Safren SA (2014). Whoonga: potential recreational use of HIV antiretroviral medication in South Africa. AIDS Behav.

[29] Niehaus IA (2007). Death before dying: understanding AIDS stigma in the South African Lowveld. J South Afr Stud.

[30] Ashforth A, Nattrass N (2005). Ambiguities of ‘culture’ and the antiretroviral rollout in South Africa. Soc Dynam.

[31] Goffman E (1963). Stigma: notes on the management of spoiled identity.

[32] Nyblade L, Singh S, Ashburn K, Brady L, Olenja J (2011). “Once I begin to participate, people will run away from me”: understanding stigma as a barrier to HIV vaccine research participation in Kenya. Vaccine.

[33] Baeten JM, Grant R (2013). Use of antiretrovirals for HIV prevention: what do we know and what don't we know?. Curr HIV/AIDS Rep.

[34] Castro A, Farmer P (2005). Understanding and addressing AIDS-related stigma: from anthropological theory to clinical practice in Haiti. Am J Public Health.

[35] Chakrapani V, Newman PA, Singhal N, Jerajani J, Shunmugam M (2012). Willingness to participate in HIV vaccine trials among men who have sex with men in Chennai and Mumbai, India: a social ecological approach. PLoS One.

[36] Abdool Karim S (2011). Stigma impedes AIDS prevention. Nature.

[37] Schneider H, Fassin D (2002). Denial and defiance: a socio-political analysis of AIDS in South Africa. AIDS.

[38] Corneli A, Perry B, Agot K, Ahmed K, McKenna K, Malamatsho F FEM-PrEP: participant explanations for non-adherence.

# Sub article: A descriptive analysis of perceptions of HIV risk and worry about acquiring HIV among FEM-PrEP participants who seroconverted in Bondo, Kenya, and Pretoria, South Africa

**DOI:** 10.7448/IAS.17.3.19152

**Published:** 2014-09-08

**Authors:** Amy L Corneli, Kevin McKenna, Jennifer Headley, Khatija Ahmed, Jacob Odhiambo, Joseph Skhosana, Meng Wang, Kawango Agot, for the FEM-PrEP Study Group

**Affiliations:** 1 Social and Behavioral Health Sciences, FHI 360, Durham, NC, USA; 2 Setshaba Research Centre, Soshanguve, South Africa; 3 Impact Research and Development Organization, Kisumu, Kenya; 4 Quantitative Sciences, Durham, NC FHI 360, USA

**Keywords:** risk perceptions, HIV worry, FEM-PrEP, seroconversion, women, Africa

## Abstract

**Introduction:**

Risk perception is a core construct in many behaviour change theories in public health. Individuals who believe they are at risk of acquiring an illness may be more likely to engage in behaviours to reduce that risk; those who do not feel at risk may be unlikely to engage in risk reduction behaviours. Among participants who seroconverted in two FEM-PrEP sites – Bondo, Kenya, and Pretoria, South Africa – we explored perceived HIV risk and worry about acquiring HIV prior to HIV infection.

**Methods:**

FEM-PrEP was a phase III clinical trial of once-daily, oral emtricitabine and tenofovir disoproxil fumarate for HIV prevention among women in sub-Saharan Africa. We asked all participants about their perceived HIV risk in the next four weeks, prior to HIV testing, during a quantitative face-to-face interview at enrolment and at quarterly follow-up visits. Among participants who seroconverted, we calculated the frequencies of their responses from the visit conducted closest to, but before, HIV acquisition. Also among women who seroconverted, we conducted qualitative, semi-structured interviews (SSIs) at weeks 1, 4 and 8 after participants’ HIV diagnosis visit to retrospectively explore feelings of HIV worry. Applied thematic analysis was used to analyse the SSI data.

**Results:**

Among participants who seroconverted in Bondo and Pretoria, 52% reported in the quantitative interview that they had no chance of acquiring HIV in the next four weeks. We identified four processes of risk rationalization from the SSI narratives. In “protective behaviour,” participants described at least one risk reduction behaviour they used to reduce their HIV risk; these actions made them feel not vulnerable to HIV, and therefore they did not worry about acquiring the virus. In “protective reasoning,” participants considered their HIV risk but rationalized, based on certain events or beliefs, that they were not vulnerable and therefore did not worry about getting HIV. In “recognition of vulnerability,” participants described reasons for being worried about getting HIV but said no or limited action was taken to reduce their perceived vulnerability. Participants with “no rationalization or action” did not describe any HIV worry or did not engage in HIV risk reduction behaviours.

**Conclusions:**

Women who are at substantial risk of acquiring HIV may underestimate their actual risk. Yet, others who accurately understand their HIV risk may be unable to act on their concerns. Perceived HIV risk and risk rationalization are important concepts to explore in risk reduction counselling to increase the use of HIV prevention strategies among women at risk of HIV.

## Introduction

An individual's perception of risk for a particular illness is a core construct in many behaviour change theories in public health [1, 2]. Although perception of risk is one of several theoretical constructs that may influence behaviour change, the main premise is that individuals who believe they are at risk of acquiring an illness may be more likely to engage in behaviours to reduce that risk. Conversely, individuals who do not believe they are at risk may be unlikely to modify their risk behaviours. Perception of HIV risk has been studied extensively in the field of HIV prevention [3–13]. Yet, limited data are available on individuals’ perceptions of their HIV risk around the time of HIV infection.

In FEM-PrEP [14], we explored perceptions of HIV risk among participants at multiple times throughout the trial. We also retrospectively explored HIV worry among participants who seroconverted. We wanted to learn more about how women who were at substantial HIV risk understood and contextualized their HIV risk, as these beliefs may influence use of HIV risk reduction methods. In this article, we focus on HIV risk perceptions and worry about HIV acquisition among participants who seroconverted during the trial from two sites – Bondo, Kenya, and Pretoria, South Africa. We first report how participants who seroconverted perceived their HIV risk shortly before they became infected with HIV. We then describe the context surrounding perceived HIV risk among participants by describing reasons for worrying or not worrying about acquiring HIV prior to becoming infected.

## Methods

### Overview of the FEM-PrEP clinical trial

FEM-PrEP was a phase III, placebo-controlled clinical trial to assess the safety and effectiveness of once-daily, oral emtricitabine (FTC) and tenofovir disoproxil fumarate (TDF) for the prevention of HIV among women. The trial was conducted in Bondo, Kenya; in Bloemfontein and Pretoria, South Africa; and in Arusha, Tanzania. Details of the trial are described elsewhere [14]. Briefly, women considered to be at higher risk for HIV acquisition (i.e. those who either had vaginal sex at least once within the past two weeks or had sex with more than one sexual partner in the past month) were randomized to once-daily FTC-TDF or placebo. Participants were asked to take their assigned study product for 52 weeks and to attend clinic visits every four weeks for 60 weeks. Among other trial procedures, participants were tested for HIV and received risk reduction counselling, a free supply of condoms and treatment for any curable sexually transmitted infections at each visit. Sixty-eight participants (34 from Pretoria, 27 from Bondo and 7 from Bloemfontein) became infected with HIV on or before week 52. Participants who seroconverted were asked to attend clinic visits one week after their HIV diagnosis visit and every four weeks for 52 weeks.

Qualitative research on adherence, sexual behaviours and HIV risk perceptions was embedded within the clinical trial protocol at the Bondo, Pretoria and Arusha sites. The trial began in July 2009 and closed early in April 2011 because of lack of effectiveness [15]. At closure, the Bondo and Pretoria sites were fully enrolled, and most participants had completed or nearly completed their follow-up visits; the Arusha site had recently initiated; and the Bloemfontein site was still enrolling, and no participant there had completed all follow-up visits.

### Data collection

#### Quantitative interviews

We conducted a quantitative, face-to-face interview on perception of HIV risk with all participants at enrolment and at quarterly follow-up visits. We asked participants to report on the likelihood that they would become infected with HIV in the next four weeks. Response options were 1) no chance – no possibility of becoming infected with HIV; 2) a small chance – could happen but not likely; 3) a moderate chance – some possibility of becoming infected; and 4) a high chance – likely to become infected. The interview was conducted at the beginning of the study visit, prior to participants learning their HIV test results; the questions on risk perceptions were no longer asked once a participant had seroconverted. We also asked demographic, sexual behaviour and relationship questions at baseline through a quantitative face-to-face interview. All quantitative interviews were conducted at the study clinic and in the local language (Setswana in Pretoria, and Dholuo or Kiswahili in Bondo) or in English by trained, local, female staff interviewers.

#### Qualitative interviews

We conducted semi-structured interviews (SSIs) with participants who seroconverted at the Bondo and Pretoria sites to retrospectively explore, among other topics, HIV worry – a measure of perceived vulnerability [16] and a concept similar to HIV risk perception [17, 18]. All participants who seroconverted at these sites were invited to participate in SSIs at weeks 1, 4, and 8 after their HIV diagnosis visit. We chose these weeks based on previous experience in conducting interviews at similar time points with individuals diagnosed with acute HIV infection in Malawi [19]. In the SSIs at week 1, participants were asked to describe whether they worried (prior to their diagnosis) about becoming infected with HIV, the reasons why they did or did not worry and whether they took any actions because of their perceived vulnerability. Participants who missed their week 1 interview or did not discuss HIV worry during their week 1 interview were asked about HIV worry at the subsequent week 4 or week 8 interview. The SSIs were audio-recorded if the participant agreed; detailed notes were taken otherwise. Trained, local female interviewers, who were different from those who conducted the quantitative interviews, conducted the SSIs at the study clinic in the local language or in English.

### Data analysis

Among the 61 participants who seroconverted in Bondo and Pretoria, we calculated the frequencies of their responses on perceived HIV risk that were reported at the study visit that was conducted closest to, but before, HIV acquisition (based on the estimated window dates of HIV infection [14, 20]). Of these participants, 56 (32 from Pretoria and 24 from Bondo) participated in at least one SSI, for an inclusion rate of 94% for Pretoria and 89% for Bondo. No participant refused to participate in an SSI; missed interviews occurred when participants did not return for their follow-up study visits.

Applied thematic analysis was used to analyse the qualitative data [21]. Interviewers simultaneously transcribed and translated audio-recorded SSIs into word-for-word English transcripts (while maintaining the overall meaning of the participants’ narratives) following a transcription protocol [22]; 51 participants agreed for at least one of their interviews to be audio-recorded. For SSIs that were not audio-recorded, interviewers expanded their detailed notes immediately after the interview and categorized the discussion by content area.

To better understand the overall context of HIV worry or non-worry, we reviewed all direct responses to questions on HIV worry as well as other related behavioural data provided by each participant in the SSIs. Using NVivo 9 [23], two qualitative analysts applied a content code on “HIV worry/non-worry” to capture text from the SSIs in which participants spoke about whether or not they worried about HIV prior to their HIV diagnosis and to capture other related narratives. Inter-coder reliability (ICR) was assessed throughout the content-coding process; 22 transcripts were assessed across the week 1, 4 and 8 interviews. Independent coding occurred after the first three ICR sessions, at which point the analysts had consistently and repeatedly reached agreement on almost all code applications. The remaining four ICR sessions were interspersed throughout the coding period. At each ICR session, coding discrepancies were discussed and resolved, and the codebook and previously coded transcripts were revised if necessary.

When coding was completed, the content-coding reports were reviewed, and a matrix-based approach was used to display reasons that participants gave for worrying or not worrying about acquiring HIV. Using the matrix, we identified themes such as “having only one partner” and “trusting partner” that led participants to worry or not worry about acquiring HIV. The frequencies of each theme and representative illustrative quotes were then summarized in a data memo. Based on the similarities of the themes and perceived vulnerability, we grouped each theme into broad risk rationalization categories. Data from five participants (three from Pretoria and two from Bondo) were excluded because we were unable to discern any meaning about vulnerability from their narratives; thus, the total SSI sample size is 51 (29 from Pretoria and 22 from Bondo).

### Ethics

All associated ethics and regulatory committees approved the trial. All participants provided their informed consent to participate in the SSIs when they signed the clinical trial enrolment consent form; before each SSI, participants were verbally asked if they were still willing to be interviewed.

## Results

### Baseline characteristics among participants who seroconverted in Bondo and Pretoria

Twenty-eight (82%) of the participants in Pretoria were under 25 years of age, compared with 12 (44%) of the participants in Bondo (Table 1). Participants in Pretoria also had more years of schooling (mean: 10.7 years) than participants in Bondo (mean: 8.2 years). Occupations also varied among the sites. A high percentage of participants in Bondo were daily wage earners (63%, *n =* 17), such as through employment at a restaurant or bar or by selling fish; most participants in Pretoria (65%, *n =* 22) reported not having an occupation. Table 1 also describes sexual behaviours and beliefs about sexual partners reported during the quantitative questionnaire that was conducted at enrolment. In the SSIs, 41 (73%) of the participants (*n =* 16 in Bondo; *n =* 25 in Pretoria) described having only one sexual partner around the time of HIV infection.

**Table 1:** Baseline characteristics among participants who seroconverted in Bondo, Kenya, and Pretoria, South Africa

Variables^a^	Pretoria (*n=*34)	Bondo (*n=*27)
Age in years		
<25	28 (82)	12 (44)
≥25	6 (18)	15 (56)
Education		
Finished primary school	28 (82)	4 (15)
Education in years^b^	10.7 (11), 4–14	8.2 (8), 3–16
Married	1 (3)	16 (59)
Occupation		
None	22 (65)	5 (19)
Student	10 (29)	2 (7)
Daily wage job	2 (6)	17 (63)
Others^c^	0	3 (11)
**Sexual behaviours at enrolment** ^**d**^		
Type of partner		
Primary partner *only*	27 (79)	16 (59)
More than one sexual partner^e^	7 (21)	11 (41)
No. of vaginal sex partners in past 7 days^b^	1.0 (1), 0–2	1.0 (1), 0–2
No. of vaginal sex acts with primary partner in past 7 days^b,f^	3.4 (2), 0–12	2.9 (2), 0–8
No. of vaginal sex acts with other partner in past 7 days^b,g^	0.3 (0), 0–4	0.5 (0), 0–6
Had sex without condom with primary partner^f^	15/34 (44)	22/27 (82)
Had sex without condom with other partner^g^	0/6 (0)	3/11 (27)
Had anal sex with primary or other partner	0/33 (0)	1/27 (4)
Exchanged sex for money or goods^g^	3/6 (50)	5/11 (46)
**Beliefs about sexual partner at enrolment**		
Believed primary partner has HIV^f^		
No	22 (65)	14 (52)
Yes	1 (3)	0
Do not know	11 (32)	13 (48)
Believed primary partner had vaginal or anal sex with other sexual partners in the past 4 weeks^f^		
No	13 (38)	3 (11)
Might/yes	2 (6)	10 (37)
Do not know	19 (56)	14 (52)
Believed other sexual partner in the past 4 weeks had HIV^g^	(*n=*6)	(*n=*11)
No	2 (33)	0
Yes	0	0
Do not know	4 (67)	11 (100)

a *n* (%) reported unless specified

b mean (median), range

c all participants in other category in Bondo are farmers

d reported for the past 4 weeks unless otherwise noted

e primary partner plus at least one other partner or two or more other partners

f among participants who reported having a primary partner

g among participants who reported having other partners.

### HIV risk perceptions and worry

Fifty-two percent (*n =* 32) of participants reported in the quantitative interview, which was conducted at the visit closest to (but before) becoming HIV infected, that they had no chance of acquiring HIV in the next four weeks. Perception of HIV risk varied significantly between the two sites (*p <* 0.0001, using a chi-square test.) (Figure 1). Prior to becoming infected with HIV, the participants in Pretoria who seroconverted had generally perceived themselves to be at less risk than had the participants in Bondo who seroconverted; 24 (71%) of the participants in Pretoria compared with 8 (30%) of the participants in Bondo indicated that they had no chance of acquiring HIV in the next four weeks. Conversely, 11 (41%) of the participants in Bondo compared with one participant (3%) in Pretoria believed they had a high chance of acquiring HIV.

**Figure 1:** Perception of HIV risk reported at the visit conducted closest to, but before, HIV acquisition, by site.

The findings about HIV worry from the SSIs were similar to the findings about risk perceptions from the quantitative interviews. Most participants in Pretoria (*n =* 25) said that they did not worry about getting HIV prior to their HIV diagnosis, but many participants in Bondo (*n =* 10) said they did. Additionally, 24 of the participants (mostly from Pretoria) in the SSIs said they *never* thought they would get infected with HIV.

### Risk rationalizations

We identified and labelled four processes of risk rationalization from the thematic analysis of the 51 participant narratives in the SSIs: 1) protective behaviour, 2) protective reasoning, 3) recognition of vulnerability and 4) no rationalization or action. These processes of risk rationalization illuminate participants’ perceptions of their risk context and, for some, how risk management strategies, or a lack thereof, influenced their sense of vulnerability towards acquiring HIV.

#### Protective behaviour

In the SSIs, 14 participants (nine from Pretoria and five from Bondo) described that they engaged in well-known HIV risk reduction practices; these actions made them feel not vulnerable to HIV, and therefore they did not worry about acquiring the virus. SSI narratives that focused on protective behaviours were mostly by participants in Pretoria who reported in the quantitative interviews that they had no chance of acquiring HIV.

Condom use was one of two commonly mentioned protective behaviours in the SSIs that led many of these participants (*n=*7) to not worry about getting HIV. For example, a participant from Pretoria was confident in her reasoning for previously not worrying about getting HIV: “We used to use a condom when we had sex. Ja. So I was sure that whatever he may do, I know I protect myself.” Yet, later in the interview she described inconsistent condom use, which she believed led her to become infected: “We woke up at night and he wanted us to have sex. I never thought about a condom … [but] when we were using a condom, we were okay.”

Having only one sexual partner was the other protective behaviour frequently mentioned (*n=*9). A participant from Pretoria said: “I never thought I would get infected with HIV. My own behaviour did not give it a chance. I only had one partner, and I never slept around.” Another participant from Pretoria said: “I never got worried … I knew myself.” She elaborated on her reason for not previously worrying about HIV when describing her response to learning she had HIV: “I never thought I could be infected by this … [because] of the way I behaved myself … My condition was good. I behaved myself very well. I have one partner.”

Only one participant, who was from Bondo, mentioned that taking the study pill led her to not worry about acquiring HIV. In her SSIs, she described that the purpose of the two-arm study was to assess whether FTC-TDF could reduce the risk of HIV acquisition, but she hoped she was assigned FTC-TDF because she believed it would work for HIV prevention. Prior to seroconversion, she believed she was taking FTC-TDF because she had repeated HIV-negative test results. After seroconversion, however, she believed she was assigned the placebo because she acquired HIV.

#### Protective reasoning

Narratives in the SSIs suggested that 23 participants (16 from Pretoria and 7 from Bondo) actively reflected upon their HIV risk but rationalized, because of certain events or beliefs, that they were not vulnerable to acquiring HIV, and therefore they did not worry about the possibility of infection. In most cases, this rationalization led participants to think that continued risk reduction behaviours, such as using condoms, were unnecessary. The majority of these SSI narratives were from participants in Pretoria who reported no chance of acquiring HIV in their quantitative interviews.

Perceptions about HIV testing influenced SSI participants’ protective reasoning. Many participants (*n=*10) described that having multiple HIV-negative test results before or during the trial led them to not worry about acquiring HIV. A participant from Bondo commented: “I have never thought of that [worry about HIV]. Depending on the days that I have always come for my visits, I always tested negative. Even when I came I never thought I would test HIV positive.” Some participants (*n=*4) described that knowing their partner's previous HIV-negative status made them not worry. A participant from Pretoria explained:

I don't know what to say, but I did not expect that at this moment I would be HIV-positive … The person I was involved with got tested and the results came out negative. I was also getting tested and I was negative so I did not expect it. I don't know – maybe he hid it from me because you can be tested and there is still a window period.

Assuming to know a partner's HIV status based on one's own HIV status was also mentioned by some participants (*n=*3) in Pretoria: “I never thought that my boyfriend could be HIV infected. I knew that I was not infected with HIV, so I never thought that he might be sick.”

Trust was another concept that informed several participants’ protective reasoning (*n=*9). Trust related to HIV worry was multifaceted, was often interwoven with HIV testing and was described primarily by participants in Pretoria. Some participants (*n=*3) described trusting their partners because they assumed they were in monogamous relationships. A participant from Pretoria who had multiple non-concurrent relationships said: “I trusted my boyfriends … because I did not see them cheating on me.” Furthermore, two participants also believed their partners would not infect them – a participant from Bondo explained: “I also have one sexual partner, whom I trusted and knew cannot make me get there [be HIV positive]. And, I also would not like to put him there.”

One participant from Pretoria explained that trust was built over time through repeated, back-to-back HIV-negative test results: “Because most of the time I knew myself … and then I went [for HIV testing] after three months – isn't it that they say window period? They told me to come after three months. I went back; then that's when we trusted each other.” Later in the interview, the participant further described how this trust halted condom use with her partner:

It's that I am with one person. The first time we had sex we were using condoms. And then he told me that we have to stop using condoms; but before [we stop] we have to test. We tested many times and they were negative. So, that's when I started trusting him, and then I ended up being positive.

For some participants (*n=*3), trust was also formed based on assumptions they had about their partners’ sexual behaviours given their own sexual behaviours. For example, a participant from Pretoria described that she did not worry about getting HIV because she was faithful and believed her partner was as well: “I knew I was behaving well and I thought he was behaving well too.”

Narratives from three participants in Bondo included statements that demonstrated both protective behaviours and protective reasoning. These participants were grouped in both categories above. All other participants were counted in only one of the risk rationalization processes.

#### Recognition of vulnerability

Eleven participants (1 from Pretoria and 10 from Bondo) described in the SSIs specific reasons why they worried about getting HIV and suggested that they were unable to consistently engage in risk reduction behaviours to reduce their perceived vulnerability. Most of these participants perceived their risk to be moderate or high in the quantitative interviews.

Participants described two main reasons for their HIV worry: 1) knowing that their partners have other sexual partners, or being uncertain of their partners’ monogamy (*n=*7); and 2) not knowing their partners’ HIV status (*n=*6). A participant from Bondo explained: “That [HIV] is something I knew was there and I could get it at any time … because I know my status but I don't know his status. I don't know his sexual behaviour. I just know my sexual behaviour.”

All participants described infrequent or inconsistent condom use (*n=*7) – or never using condoms at all (*n=*4). A participant from Bondo explained that not using a condom within the overall context of uncertainty about her partner's fidelity led her to worry: “I had thought of that [worry about HIV] because my husband and I had got tested a long time ago and we do not use condoms, but I don't know how his movements are and if he has other sexual partners.” Many participants (*n=*6) described that their sexual partners disliked using condoms and they were therefore using them infrequently, if at all. Cultural traditions and community gender roles, including limited sexual decision-making power among women, were also described by a participant from Bondo as a reason why she worried about getting HIV:

It was worrying me based on how I was told that woman [her husband's other wife] was behaving … I had even told him, I told him clearly that ‘imagine you will get infected with a disease that you don't even know’. I used to sit down to talk softly with my husband, but he could not listen to my opinion because you know I am a woman and he is a man. So our thoughts could not rhyme. So I was really worried.

Earlier in the interview, the participant explained that because of Luo customs, she was unable to use a condom with her husband during certain rituals.

#### No rationalization or action

Narratives from six participants (three from Pretoria and three from Bondo) lacked any mention of prior rationalization of HIV risk or examples of actions to reduce the chance of HIV acquisition. Four participants described that they never thought they would become infected; two of the four described being in long term relationships.

## Discussion

During the FEM-PrEP clinical trial, we were able to assess participants’ perceptions of their HIV risk during the period shortly before they became infected with HIV. We were also able to gather rich qualitative data on participants’ rationales for having previously worried or not worried about acquiring HIV. In the quantitative interviews, more than half of the participants who seroconverted (52%) did not perceive that they had been at any risk for HIV around the time they became infected. The majority of these participants were from Pretoria. Similarly, most participants in the SSIs who described not worrying about acquiring HIV were from Pretoria. Generally, SSI narratives suggested that participants’ own sexual behaviours and beliefs influenced their protective behaviours and protective reasoning and led these participants to not worry about acquiring HIV. Together, these findings suggest that many women who are at substantial risk of acquiring HIV may not perceive their current sexual context to be risky and therefore may underestimate their actual risk.

Several factors within the participants’ broader environment may have influenced the protective behaviours and protective reasoning they described in the SSIs, potentially leading them to believe that their current sexual context was not risky. First, the dropping rates of HIV infection in some of the study countries around the time of trial initiation may have left participants feeling less at risk or vulnerable. For instance, HIV incidence in South Africa had declined by more than 25% between 2001 and 2009. Also, prevalence in Kenya had declined to 5% by 2006 from 14% in the mid-1990s [24]; however, in 2008–2009, the prevalence was still high at 13.9% in Nyanza Province, where Bondo is located [25]. Second, we provided risk reduction counselling and HIV testing at each study visit as part of trial procedures. Receiving frequent HIV-negative test results influenced many participants’ rationalizations that they were not vulnerable to HIV and potentially led them to believe or reaffirmed their beliefs that they were not engaging in risky sexual behaviours. Third, based on the theory of cognitive dissonance [26], participants may have believed they were at risk of HIV but rationalized that they were not at elevated risk to reduce or avoid the discomfort associated with feeling at risk. Similar findings, albeit with different rationalizations, were found in a study among female sex workers in Nigeria [27]. Fourth, participants may have believed they truly were not at risk because they were following two of the three prevention steps in the widely promoted ABCs of HIV prevention – B for “Be Faithful” and C for “Use Condoms” (albeit they used condoms inconsistently) – and either assumed their partners were also following such guidelines or did not consider their partners’ behaviour. Based on these data, we conducted follow-up interviews with participants in Bondo and Pretoria to explore the broader context that may influence perceptions of faithfulness and trust; these data are to be presented elsewhere.

In contrast to the participants who may have underestimated their HIV risk, many participants who seroconverted during the FEM-PrEP trial (48%) appeared to accurately understand their HIV risk but were unable to act on their concerns. Most of these participants were from Bondo, and their beliefs about their partners’ risky sexual behaviour, as described in the SSIs, strongly influenced their own higher perception of vulnerability. Concerns about spousal infidelity have also been shown to influence HIV worry among women, and men, in Malawi [28]. Gender-based power dynamics, as have frequently been documented in the literature, often impede a woman's ability to engage in risk reduction behaviours, particularly using condoms with her primary partner, in marriage, or within inherited relationships (which are common among widows in Bondo) [29–38].

Because we collected quantitative data on risk perceptions during participants’ quarterly visits, responses may have reflected participants’ perceptions for up to 12 weeks before they became infected with HIV. It is possible that perceptions of risk for some participants may have increased or decreased immediately prior to HIV infection given a change in their risk context. However, in the SSIs, most participants said that their sexual behaviour and that of their partners remained similar in the months prior to HIV infection. Furthermore, because of the likely emotional response to learning one's HIV status, recall bias or a bias related to self-protection may also have affected how some participants retrospectively reflected on their HIV worry prior to diagnosis.

Based on our findings, we recommend that future risk reduction interventions consider how to incorporate women's HIV risk perceptions, particularly their risk rationalizations, into the HIV prevention discourse [39, 40], as they are likely important precursors for adopting any risk reduction measures. This is especially true for women who are similar to the study participants in the Pretoria site, where many participants generally had low perceptions of HIV risk and low perceived vulnerability towards acquiring HIV, but where a high overall HIV incidence (6.0 per 100 person years in the placebo arm) was reported in the trial [14]. A significant challenge in doing so, however, will be aligning women's feelings about their risk with their actual level of risk [41]. For women who are not in a recognized serodiscordant relationship, exposure to HIV is often unknown. Moreover, many women may have little HIV risk or not be at risk of HIV at all (e.g. when partners are HIV negative, and in monogamous relationships between HIV-negative individuals). Therefore, in HIV risk reduction counselling and other HIV prevention programmes, an intricate balance is needed to simultaneously 1) encourage women to more appropriately evaluate the potential likelihood of becoming infected with HIV; 2) ensure that couple harmony is maintained [42] (e.g. not introduce unnecessary conflict within the relationship when HIV risk is actually low) and the relationship is preserved (e.g. among women who want to or who must remain with their partners because of social or financial consequences); and 3) prevent other adverse consequences, such as denial and defensive coping [27], of asking women who may be at actual HIV risk to reassess their potential risk.

For women in risk situations similar to those of many of the participants in Bondo, where perceived HIV risk, vulnerability towards HIV and HIV incidence (4.7 per 100 person years in the placebo arm [14]) were generally high, a woman-controlled HIV risk reduction method, such as pre-exposure prophylaxis (PrEP) and potentially microbicides and the vaginal ring (pending current trial outcomes [43–45]), may be more realistic. Moreover, interventions that focus on 1) empowering women and increasing economic independence while transforming gender norms, 2) encouraging safer sexual health practices within marriage and 3) incorporating male involvement into the promotion and use of women-centred approaches [34, 36, 37, 46–48] must continue and intensify.

## Conclusions

In moving forward with HIV prevention among women, whether in PrEP demonstration projects or in non-biomedical HIV prevention programmes, perceived HIV risk and risk rationalization are important concepts to explore. Understanding and responding to women's perceptions and rationalizations about risk could enhance the use of risk reduction methods, particularly in populations similar to those in the FEM-PrEP clinical trial.

## References

[1] Rosenstock I (1974). The health belief model and preventive health behavior. Health Educ Monogr.

[2] Rogers RW, Cacioppo JR, Perry RE (1983). Cognitive and physiological processes in fear appeals and attitude change: a revised theory of protection motivation. Social psychology: a sourcebook.

[3] Kowalewski MR, Henson KD, Longshore D (1997). Rethinking perceived risk and health behavior: a critical review of HIV prevention research. Health Educ Behav.

[4] Gerrard M, Gibbons FX, Bushman BJ (1996). Relation between perceived vulnerability to HIV and precautionary sexual behavior. Psychol Bull.

[5] Akwara PA, Madise NJ, Hinde A (2003). Perception of risk of HIV/AIDS and sexual behaviour in Kenya. J Biosoc Sci.

[6] Do M, Meekers D (2009). Multiple sex partners and perceived risk of HIV infection in Zambia: attitudinal determinants and gender differences. AIDS Care.

[7] Macintyre K, Rutenberg N, Brown L, Karim A (2004). Understanding perceptions of HIV risk among adolescents in KwaZulu-Natal. AIDS Behav.

[8] Maharaj P, Cleland J (2005). Risk perception and condom use among married or cohabiting couples in KwaZulu-Natal, South Africa. Int Fam Plan Perspect.

[9] Mbizvo EM, Msuya SE, Hussain A, Chirenje MZ, Stray-Pedersen B (2003). HIV prevalence in Zimbabwean women: 54–67% knowledge and perceived risk. Int J STD AIDS.

[10] Lindan C, Allen S, Carael M, Nsengumuremyi F, Van de Perre P, Serufilira A (1991). Knowledge, attitudes, and perceived risk of AIDS among urban Rwandan women: relationship to HIV infection and behavior change. AIDS.

[11] Stringer EM, Sinkala M, Kumwenda R, Chapman V, Mwale A, Vermund SH (2004). Personal risk perception, HIV knowledge and risk avoidance behavior, and their relationships to actual HIV serostatus in an urban African obstetric population. J Acquir Immune Defic Syndr.

[12] MacKellar DA, Valleroy LA, Secura GM, Behel S, Bingham T, Celentano DD (2007). Perceptions of lifetime risk and actual risk for acquiring HIV among young men who have sex with men. AIDS Behav.

[13] Tenkorang EY, Rajulton F, Maticka-Tyndale E (2009). Perceived risks of HIV/AIDS and first sexual intercourse among youth in Cape Town, South Africa. AIDS Behav.

[14] Van Damme L, Corneli A, Ahmed K, Agot K, Lombaard J, Kapiga S (2012). Preexposure prophylaxis for HIV infection among African women. N Engl J Med.

[15] FHI 360 (2011). FHI statement on the FEM-PrEP HIV prevention study [Internet]. http://www.fhi360.org/fhi-statement-fem-prep-hiv-prevention-study.

[16] Cummings GL, Battle RS, Barker JC, Krasnovsky FM (1999). Are African American women worried about getting AIDS? A qualitative analysis. AIDS Educ Prev.

[17] Brewer NT, Chapman GB, Gibbons FX, Gerrard M, McCaul KD, Weinstein ND (2007). Meta-analysis of the relationship between risk perception and health behavior: the example of vaccination. Health Psychol.

[18] Smith KP, Watkins SC (2005). Perceptions of risk and strategies for prevention: responses to HIV/AIDS in rural Malawi. Soc Sci Med.

[19] Pettifor A, MacPhail C, Corneli A, Sibeko J, Kamanga G, Rosenberg N (2011). Continued high risk sexual behavior following diagnosis with acute HIV infection in South Africa and Malawi: implications for prevention. AIDS Behav.

[20] Van Damme L The FEM-PrEP trial: TDF/FTC (Truvada^®^) as pre-exposure prophylaxis for HIV infection among African women.

[21] Guest G, MacQueen KM, Namey EE (2011). Applied thematic analysis.

[22] McLellan E, MacQueen KM, Niedig J (2003). Beyond the qualitative interview: data preparation and transcription. Field Methods.

[23] NVivo Qualitative Data Analysis Software (2010). QSR International Pty Ltd.

[24] UNAIDS (2010). Global report: UNAIDS report on the global AIDS epidemic, 2010: regional fact sheet: Sub-Saharan Africa [Internet].

[25] Kenya National Bureau of Statistics (KNBS), ICF Macro (2010). Kenya demographic and health survey 2008–09.

[26] Festinger L (1962). Cognitive dissonance. Sci Am.

[27] Ankomah A, Omoregie G, Akinyemi Z, Anyanti J, Ladipo O, Adebayo S (2011). HIV-related risk perception among female sex workers in Nigeria. HIV AIDS (Auckl).

[28] Smith KP (2003). Why are they worried? Concern about AIDS in rural Malawi. Demogr Res.

[29] Anglewicz P, Clark S (2013). The effect of marriage and HIV risks on condom use acceptability in rural Malawi. Soc Sci Med.

[30] Krishnan S, Dunbar MS, Minnis AM, Medlin CA, Gerdts CE, Padian NS (2008). Poverty, gender inequities, and women's risk of human immunodeficiency virus/AIDS. Ann N Y Acad Sci.

[31] Bandali S (2011). Norms and practices within marriage which shape gender roles, HIV/AIDS risk and risk reduction strategies in Cabo Delgado, Mozambique. AIDS Care.

[32] Chimbiri AM (2007). The condom is an ‘intruder’ in marriage: evidence from rural Malawi. Soc Sci Med.

[33] Pettifor AE, Measham DM, Rees HV, Padian NS (2004). Sexual power and HIV risk, South Africa. Emerg Infect Dis.

[34] Jewkes RK, Dunkle K, Nduna M, Shai N (2010). Intimate partner violence, relationship power inequity, and incidence of HIV infection in young women in South Africa: a cohort study. Lancet.

[35] Shai NJ, Jewkes R, Nduna M, Dunkle K (2012). Masculinities and condom use patterns among young rural South Africa men: a cross-sectional baseline survey. BMC Public Health.

[36] Greig A, Peacock D, Jewkes R, Msimang S (2008). Gender and AIDS: time to act. AIDS.

[37] Small E, Nikolova SP, Narendorf SC (2013). Synthesizing gender based HIV interventions in Sub-Sahara Africa: a systematic review of the evidence. AIDS Behav.

[38] Perry B, Oluoch L, Agot K, Taylor J, Onyango J, Ouma L (2014). Widow cleansing and inheritance among the Luo in Kenya: the need for additional women-centred HIV prevention options. J Int AIDS Soc.

[39] Klein H, Elifson KW, Sterk CE (2003). “At risk” women who think that they have no chance of getting HIV: self-assessed perceived risks. Women Health.

[40] Buunk BP, Dijkstra P (2001). Rationalizations and defensive attributions for high-risk sex among heterosexuals. Patient Educ Couns.

[41] Hankins C (1998). Changes in patterns of risk. AIDS Care.

[42] Tolley EE, Eng E, Kohli R, Bentley ME, Mehendale S, Bunce A (2006). Examining the context of microbicide acceptability among married women and men in India. Cult Health Sex.

[43] FACTS Consortium FACTS 001 study design [Internet].

[44] Baeten J, Palanee T (2011). MTN-020: a multi-center, randomized, double-blind, placebo-controlled phase 3 safety and effectiveness trial of a vaginal matrix ring containing dapivirine for the prevention of HIV-1 infection in women [Inernet].

[45] International Partnership for Microbicides (IPM) (2011). Phase III sister studies of a microbicide ring to prevent HIV: the ring study & ASPIRE [Internet].

[46] Dunkle KL, Jewkes R (2007). Effective HIV prevention requires gender-transformative work with men. Sex Transm Infect.

[47] Greig FE, Koopman C (2003). Multilevel analysis of women's empowerment and HIV prevention: quantitative survey results from a preliminary study in Botswana. AIDS Behav.

[48] Harrison A, Short SE, Tuoane-Nkhasi M (2014). Re-focusing the gender lens: caregiving women, family roles and HIV/AIDS vulnerability in Lesotho. AIDS Behav.

# Sub article: ARV-based HIV prevention for women – where we are in 2014

**DOI:** 10.7448/IAS.17.3.19154

**Published:** 2014-09-08

**Authors:** Timothy D Mastro, Nirupama Sista, Quarraisha Abdool-Karim

**Affiliations:** 1 FHI 360, Durham, North Carolina, USA; 2 CAPRISA, Durban, South Africa

**Keywords:** HIV, AIDS, antiretroviral agents, women, HIV prevention, HIV treatment, PrEP

## Abstract

Women continue to be at special risk for HIV acquisition due to a complex mix of biological, behavioural, structural, cultural and social factors, with unacceptable rates of new infection. Scientific advances over the past decade have highlighted the use of antiretroviral (ARV) drugs as pre-exposure prophylaxis (PrEP) to prevent HIV acquisition (sexually, parenterally and vertically) and ARV treatment (ART) for HIV-positive patients to prevent onward transmission (treatment as prevention – TasP). This paper reviews the evidence base for PrEP and TasP, describes new products in development and the need to translate research findings into programmes with impact at the population level.

## Introduction

In the fourth decade of the HIV epidemic, the control of HIV transmission remains a global challenge – in 2012, there were an estimated 2.3 million new HIV infections globally [1]. Women continue to be at special risk for HIV acquisition due to a complex mix of biological, behavioural, structural, cultural and social factors [1]. Young women bear a disproportionate burden of HIV infection, notably in sub-Saharan Africa, home to about 70% of new infections [1]. Over the past decade, numerous prospective studies of African women in communities with high HIV infection risk have documented annual HIV incidence rates of 4 to 9%, despite the use of the best available HIV prevention methods [2–4]. A better understanding of the factors contributing to this high HIV risk is needed to guide the use of available HIV prevention tools and to design improved modalities.

Scientific advances in recent years have highlighted the use of antiretroviral (ARV) drugs as pre-exposure prophylaxis (PrEP) to prevent HIV acquisition (sexually, parenterally and vertically) and ARV treatment (ART) for HIV-positive patients to prevent onward transmission (treatment as prevention – TasP). Clinical trials have added greatly to our body of knowledge and several positive findings have raised hopes that effective use of ARVs for prevention, along with other prevention tools, can lead to control of the epidemic. However, programmatic implementation and scale-up are needed to translate these encouraging findings into population level impact. This review provides a synopsis of the scientific evidence for the use of ARVs for prevention, lessons learned from the conduct of these trials, the current state of implementation of ARV-based prevention interventions and an overview of the next generation of ARV-based products for HIV prevention.

## The evidence

### Pre-exposure prophylaxis

Research efforts over the past decade have yielded extensive evidence on ARVs for HIV prevention in women (Table 1). In 2010, the CAPRISA 004 clinical trial of 1% tenofovir vaginal gel demonstrated a 39% level of protection for women in KwaZulu-Natal, South Africa, the first study to show that a vaginal microbicide and an ARV-based product could prevent HIV infection [2]. Since then, we have seen a growing body of evidence of the prevention success of oral tenofovir-based PrEP, either tenofovir disoproxil fumarate (TDF) alone or in combination with emtricitabine (FTC) [5–7]. Daily oral PrEP provided protection for women in serodiscordant partnerships in Kenya and Uganda in the Partners PrEP study (efficacy of 75% for FTC/TDF and 67% for TDF; similar for women and men) [6] and for women in Botswana in the Centers for Disease Control and Prevention (CDC) TDF-2 study (62% efficacy for FTC/TDF among women and men) [7]. Clinical trials also showed that tenofovir-based oral PrEP can be effective in decreasing HIV incidence among men who have sex with men (MSM) [5], and in people (80% men) who inject drugs in Bangkok, Thailand [8].

**Table 1:** Effectiveness studies for ARV-based HIV prevention^a^

Study name	Location	Population	Product	Efficacy (CI)	Adherence estimates^c^	Notes	Adherence intervention
CAPRISA 004 [2]	S. Africa	889 women	Peri-coital vaginal TFV gel^b^	39% (6–60%)	51%	Efficacy was 54% among highly adherent (used gel in >80% of sex acts)	Monthly adherence counselling, applicator count and self-reported coital frequency
iPrEx [5]	North and South America, South Africa, Thailand	2499 men or transgender women who have sex with men	Once-daily oral FTC/TDF b	44% (15–63%)	51%	For first 8 weeks, self-reported pill use was slightly lower in the FTC/TDF arm than in placebo. Afterwards, they were similar, each with a mean of 95%. Rate of pill use, according to pill count, was also similar after week 8; the median ranged from 89–95%. Dispensation dates and quantities of pills suggest pill use decreased from a rate of 99% in the first year to 91% at study conclusion	Monthly adherence counselling, medical history, pill counts
Partners PrEP Study [6]	Kenya, Uganda	4747 heterosexual serodiscordant couples	Once-daily oral FTC/TDF Once-daily oral TDF b	75% (55–87%) 67% (44–81%)	81% 83%	97% of dispensed pills were taken. Study drugs were in use in an estimated 92.1% of total follow-up time	Monthly adherence counselling, sexual behaviour assessment and pill counts
CDC 4940 (TDF2) [7]	Botswana	1200 young men and women	Once-daily oral FTC/TDF	62% (22–84%)	81%	Drug efficacy was highest among highly adherent (those who had taken medication in the past 30 days). Similar rates were found in the intervention and control groups. (Pill counts were 84.1% in FTC/TDF and 83.7% in placebo. Self-reported use was 94.4% and 94.1%, respectively.)	Monthly adherence counselling, self-reported coital frequency and condom use
FEM-PrEP [3]	Kenya, S. Africa, Tanzania	1950 women	Once-daily oral FTC/TDF	6% (−52–41%)	24%	Self-reported data, at discontinuation, indicated 95% of participants had usually or always taken assigned drug. Pill count suggested 88% adherence, yet blood drug level was much lower	Monthly, adherence counselling, self-report, blood samples and pill counts
Bangkok Tenofovir Study [8]	Thailand	2413 men and women who inject drugs	Once-daily oral TDF	49% (10–72%)	66%	Participants chose either 1) daily DOT or 2) monthly visits with study drug diary accounts. They could also switch between these regimens at monthly visits	Monthly adherence counselling, DOT option
VOICE (MTN 003) [4]	S. Africa, Uganda, Zimbabwe	5029 women	Once-daily oral FTC/TDF Once-daily oral TDF Daily vaginal TFV gel	−4% (−49–27%) −49% (−129–3%) 15% (−21–40%)	29% 28% 22%	Adherence estimates are based on a subset of 773 participants. Despite an estimate of 90% adherence (based on self-reported data and pill counts), approximately 30% of participants never had the drug detected	Monthly adherence counselling, self-report and pill counts
HPTN 052 [9]	Malawi, Zimbabwe, Botswana, Kenya, S. Africa, Brazil, Thailand, US, India	1763 heterosexual serodiscordant couples	Early ART (CD4 count of 350–550 cells/mm) vs. delayed ART (CD4 count below 250 cells/mm or development of an AIDS-defining illness)	96% (73–99%)	79% of participants who were in early ART and 74% who were in delayed ART	Adherence to at least 95% study regimen was measured by pill counts	Monthly adherence counselling, self-report and pill counts

a For prevention of sexually transmitted infection

b 1% vaginal tenofovir gel (TFV), oral tenofovir (TDF), oral emtricitabine/tenofovir (FTC/TDF)

c adherence estimates for all but HPTN 052 are based on measuring drug levels from participant samples; estimates for all but the Bangkok Tenofovir Study are from reference [10].

With the exception of the CAPRISA 004 trial that used a coitally linked dosing strategy of tenofovir gel, trials have used a daily dosing strategy. Data from completed PrEP (topical and systemic) trials demonstrate a protective effect of PrEP that ranges from 39 to 75% when adherence is sufficient. However, while ARVs prevent HIV transmission, they have to be used to be effective [10]. In studies that have demonstrated efficacy, there is a clear dose relationship between increased product use and increased levels of protection [11, 12]. The modest effectiveness of 39% in CAPRISA 004 was increased to 54% in those with high levels of adherence [2] and to 74% in women in women with drug levels higher than 1000 ng/ml [13] supporting the importance of both adherence and biological factors impacting efficacy.

In the two trials that did not demonstrate PrEP efficacy, FEM-PrEP and VOICE, while self-reported adherence levels were high, measurement of drug levels indicated low levels of product use [3, 4, 14]. There are likely many factors that impact adherence and these need to be better elucidated to inform future trials and programmatic use. Interviews of women in the FEM-PrEP trial of daily, oral FTC/TDF PrEP indicated that low adherence was linked to perceptions of low risk for HIV acquisition, use of an investigational drug, fear of and/or experience of adverse effects, possible assignment to the placebo, difficulties with and dislike of taking pills and influence of other people [15].

In contrast to treatment trials that have proxy markers of HIV viral levels and CD4+ cell counts for monitoring therapeutic success, HIV prevention trials do not have a correlate of risk or protection other than HIV infection, making monitoring of the use of microbicides or PrEP products challenging. Formulation impacts drug concentrations in the genital tract; topical tenofovir gel used vaginally yields 1000-fold higher concentrations in vaginal fluids compared to oral formulations [13]. While measuring drug levels is an important advance in PrEP studies, there is no benchmark for what levels need to be achieved and sustained to prevent HIV infection. In the ART of HIV-positive persons, maintaining steady states of drug levels is key to success; for uninfected individuals the levels needed are less clearly defined. This is an important opportunity and gap that will need to be addressed as the product pipeline, dosing strategies and formulations for testing expand.

Other concerns raised about the use of PrEP include drug resistance and compromising first-line drug regimens. Thus far, acquired ARV drug resistance has not been a substantial problem in those who have acquired HIV infection while on PrEP. However, inadvertently initiating FTC/TDF PrEP in people who are already HIV positive, especially those with acute infection, has resulted in FTC resistance [5]. On-going follow-up of persons who became HIV positive on PrEP will generate empiric evidence on disease progression and treatment outcomes with TDF/FTC containing first-line drug regimens. To date the safety profile of PrEP regimens has been good, with no major safety concerns identified [3, 5, 16, 17]. PrEP has not interfered with the effectiveness of hormonal contraceptives [3, 18].

In the United States, in 2012, based on available clinical trial data, the Food and Drug Administration (FDA) approved oral FTC/TDF for PrEP for people at high risk of sexual acquisition of HIV. The US CDC subsequently issued PrEP guidance for MSM, heterosexual men and women, and following the trial among people who inject drugs in Thailand [8], added an indication for people who inject drugs. In 2014, WHO issued PrEP guidance as part of consolidated guidelines on HIV prevention, diagnosis, treatment and care for key populations (including female sex workers). Generalized guidelines for women and country-specific regulatory filings are eagerly awaited.

## PrEP offers new hope for women

ARV-based PrEP (systemic and topical) offers a promising female-controlled method of prevention. The outcomes of the FEM-PrEP and VOICE trials highlight the need to know more about individual behaviour, optimal formulations and dosing strategies for acceptability. The central importance of adherence in PrEP success has prompted examination of its determinants [19, 20]. A systematic review of 24 phase II and phase III microbicide trials has highlighted that major reasons for non-adherence include high on-trial pregnancy rates (resulting in withdrawal of study drug), low trial retention rates, low participant perception of risk, migratory partners and trial fatigue [21]. Individual participation characteristics, including young age, may also influence adherence, as well as study product characteristics [21]. Findings from the FEM-PrEP trial suggest that some women enrolled and remained in the study for benefits other than the HIV prevention that might be offered by the study drug, including clinical care; reimbursement for study visits was reported to be a minor contributor [22]. When assessing factors related to adherence in a placebo-controlled clinical trial of a product of unproven efficacy, it is important to remember that different forces will be encountered in the programmatic use of a product proven to be effective. It is critically important to continue to characterize such factors, especially as they pertain to target at-risk populations for PrEP, and to inform the development of new technologies that rely less on adherence.

While PrEP studies to date have not identified serious safety problems, both the FEM-PrEP and VOICE trials reported substantial rates of adverse effects (including nausea and headache) suggesting that these effects may have contributed to women stopping the drugs after initially using them [3, 4]. Very low levels of adverse effects will be important to maximize PrEP uptake and adherence, as users will be otherwise healthy individuals.

Notwithstanding the need to weigh the risks and costs of PrEP and TasP and identify target populations who are likely to benefit most from PrEP, modelling exercises offer compelling estimates on its preventive benefits at a population level. Effective use of PrEP among those at highest behavioural risk could avert 3.2 million new HIV infections in sub-Saharan Africa over the next 10 years [23]; and, in South Africa, broad use of tenofovir gel could avert up to 2 million new infections and 1 million deaths over the next two decades [24]. Oral and topical PrEP can be relatively cost-effective interventions [25, 26]. In light of the continued and urgent need for HIV prevention interventions that work in women and the promise of PrEP for altering epidemic trajectories, a more concerted effort on user and provider needs and preparedness is needed.

In terms of next steps with tenofovir gel, the FACTS 001 trial, designed to confirm the results of the CAPRISA 004 trial, is underway at seven sites in South Africa and is scheduled for completion in 2015. A successful outcome could lead to licensure of tenofovir gel. The CAPRISA 008 follow-on study is simultaneously assessing the feasibility of integrating microbicide provision into family planning services and is currently underway. Draft normative guidance has been prepared by WHO, and the South African government has initiated plans for local product manufacture for public sector distribution. Discussions with the US FDA have provided guidelines of requirements for licensure.

## Next-generation products and trials

We are early in the era of ARVs for HIV prevention and we can expect that additional products and formulations in the research and development pipeline will advance to clinical trials and, hopefully, programmatic use. Alternative new drugs, regimens and formulations for PrEP are needed in order to provide choices to men and women, similar to available choices of contraceptive methods. New drugs will provide an alternative to FTC/TDF, which is efficacious and generally safe but is occasionally associated with increased creatinine levels (indicating renal impairment) and decreased bone density and is associated with side effects (e.g., nausea, headache) that may impact adherence. Furthermore, FTC/TDF is rapidly becoming part of first-line ART for many countries. Orally administered TDF (yielding TFV in plasma) does not concentrate in the cervix or vagina after a single dose and this may reduce “forgiveness” in the protection of women if doses are missed [12, 27], and the emergence of ARV resistance remains a concern. Therefore, PrEP agents that have a higher threshold for resistance and/or in which the emergence of resistance will not impact efficacy of first-line regimens are needed. Moreover, since daily PrEP, in either oral or gel formulation, may be a difficult adherence goal for some healthy individuals, finding alternative dosing regimens is a priority [28]. Therefore, development of long-acting injectable products, vaginal rings and patches is an important undertaking. Such products have the potential to prevent HIV acquisition without relying on adherence to a daily oral or gel insertion regimen.

The next generation of topical microbicides being evaluated includes products that do not require use daily or are linked to sexual events and monthly formulations. Such products could be more convenient to women and could provide long-term protection from HIV infection during unanticipated and anticipated sexual exposures. A vaginal ring that is replaced every four weeks and contains dapivirine (DAP, a non-nucleoside reverse transcriptase inhibitor [NNRTI]) in silicone elastomer is being evaluated in phase III studies (MTN 020 “ASPIRE” and IPM 027 “Ring” trials). The results of these trials are anticipated in 2015. Other rings including those that contain maraviroc (MVA, a CCR5 receptor antagonist), MIV 150 (an NNRTI) and a combination of DAP and MVA are being evaluated in phase I and phase II [29, 30]. Preliminary results indicate that the vaginal rings containing DAP, MVA or a combination of the two drugs are safe and tolerable and DAP, but not MVA, was quantifiable in vaginal tissue [29]. A film formulation of DAP was demonstrated to be safe with measureable levels of drug in plasma and tissue [31]. Other products, including a ring containing a combination of DAP and darunavir and a vaginal tablet containing TFV and FTC, are being evaluated for efficacy in preclinical studies [30].

An advantage of these long-term dosing formulations, in addition to the lack of dependence on daily adherence, is the ability to combine agents for other indications such as contraceptives or drugs for treatment of other sexually transmitted infections. Preclinical studies are underway to evaluate combinations of the contraceptive hormone levonorgestrel with either DAP or TFV [32]. Injectable agents are already being used by women for contraception. In the future, an effective PrEP agent has the potential to be combined with an effective contraceptive for prevention of both HIV infection and pregnancy. Such multi-purpose technologies formulated either as an injectable formulation or in a vaginal ring would be a valuable tool for HIV prevention in women in resource-limited settings, including sub-Saharan Africa where the majority of the world's HIV-positive women reside.

Two injectable ARV agents currently in development are TMC278LA, an injectable formulation of rilpivirine (an NNRTI), and GSK1265744, an integrase inhibitor [33, 34]. TMC278LA is a novel poloxamer 338–containing formulation of TMC278. TMC278LA is long-acting and well-suited for delivery via intramuscular injection and is currently being considered for both ART and PrEP. Studies have demonstrated the safety of oral rilpivirine, which is approved by the US FDA for treatment as well as the safety of single doses of TMC278LA. The long-acting injectable formulation is also being developed for treatment of HIV infection. Initial studies have demonstrated the safety of single doses of TMC278LA in men and women [35]. The safety of long-term dosing of TMC278LA for PrEP will be evaluated in HIV-negative women in the United States, South Africa and Zimbabwe in the HPTN 076 study (www.hptn.org).

Another injectable agent, GSK1265744, is an investigational HIV-1 integrase strand transfer inhibitor that possesses attributes favourable for both HIV treatment and PrEP indications. In macaques, single, monthly injections of GSK744 LA that replicate the human dose were fully protective against repeated vaginal SHIV exposures [36]. GSK744 LA also prevented rectal SHIV transmission in macaques [37]. These data support the advancement of GSK744 LA as a PrEP candidate for women. It is also currently in phase II clinical trials evaluating the efficacy and safety for treatment of HIV infection [33]. The safety of GSK744 LA as a PrEP agent will be evaluated in men and women at sites in the United States, Brazil and sub-Saharan Africa in the HPTN 077 study (www.hptn.org). Both TMC278 LA and GSK744 LA have a pharmacokinetic profile that allows monthly to trimonthly parenteral dosing using a nanosuspension formulation. Another injectable agent, an investigational monoclonal antibody, Ibalizumab binds to the primary receptor for HIV, CD4, and inhibits viral entry [38, 39]. It is being evaluated for the treatment of HIV infection in combination with an optimized background regimen, and it may also show promise in its utility as a PrEP agent [34].

## Treatment as prevention

The HPTN 052 clinical trial of early (CD4 cell counts of 350–500 cells/µL) ART compared to delayed treatment for HIV-positive persons with HIV-negative heterosexual partners demonstrated a 96% reduction in transmission [9]. Protection was similar for men and women; 50% of the HIV-positive partners were women [9]. This is the most compelling evidence for the effectiveness of ARVs for preventing HIV infection [9]. Modelling data demonstrate the enormous prevention benefits from implementing these findings especially in settings with a high HIV disease burden [40–43]. Access to HIV testing services and linkage to care and treatment services will be needed to reduce individual viral loads and onward transmission, but there is still uncertainty about the coverage, uptake and compliance rates that will be needed to achieve an AIDS-free generation alone and in combination with other prevention interventions.

In 2013, WHO issued new guidelines for ART that increased eligibility to HIV-positive persons with CD4 cell counts less than 500 cells/µL, all pregnant and breastfeeding women, persons with HIV-negative partners, persons with tuberculosis and hepatitis B virus infection and children less than five years of age [44]. These changes greatly increase the number of people globally who “need” ART and will strain health systems and resources to achieve full or even near-full coverage. Women should benefit from the hoped-for population-level effect on HIV transmission. However, achieving high levels of HIV testing and identifying persons with acute and early HIV infection remains challenging. On-going studies of combination HIV prevention strategies that include early ART for all HIV-positive persons are intended to measure the effect of early ART on population-level HIV incidence (TasP, POP-ART, Botswana). Even with high levels of ART coverage, it is likely that there will continue to be a pressing need for women-controlled prevention methods.

A number of trials and demonstration projects are currently underway to assess the feasibility of implementation and the impact on HIV transmission of TasP at a population level (e.g., HPTN 065 in Washington DC and the Bronx, New York; ANRS/Africa Centre in KwaZulu-Natal, South Africa; POP-ART/HPTN 071 in Zambia and South Africa; and MSF in Malawi, Swaziland and South Africa) [30]. The translation of these trial findings will be influenced by the ability to deliver TasP in settings where AIDS-related stigma and discrimination are prominent, health care delivery systems are weak and the ability to increase coverage of treatment for those who need treatment for individual benefits are limited. Additional concerns include treatment monitoring costs and the development of drug resistance due to poor adherence. Modelling exercises suggest that high levels of ART coverage (>80%) will be needed to substantially decrease population level HIV incidence [45]. However, ecological, observational level data from Hlabisa, KwaZulu-Natal, South Africa, that indicate that a modest 35% coverage of ART reduced HIV incidence by 34% are encouraging [46].

An additional concern about TasP in settings where young women bear a disproportionate burden of HIV infection as a result of age-disparate relationships with male partners who have been reluctant to practice safer sex is the male partners’ level of willingness to initiate early ART for the prevention of infection to their sexual partner. Some of these concerns were raised in considering access to ART in sub-Saharan Africa in the early 2000s and to date have not materialized. Revised HIV treatment guidelines have increased the number of people eligible for ART. With regard to HIV-TB co-infection, in some parts of Africa, HIV-TB co-infected patients account for about 70% of patients using TB services. With the expansion of ART eligibility to these patients, perhaps the issue of when to start ART will become a less controversial issue. With increased implementation and expanded coverage of HIV testing and linkage to care and treatment, those with acute HIV infection will contribute a larger proportion of all new infections. Strategies including laboratory-based assays for identifying these individuals quickly and intervening early will be central to maintaining reductions in onward transmission of HIV and gains made from TasP [20].

## Conclusions

There remains an urgent need to improve and scale-up HIV prevention efforts for women, especially in southern Africa. We have seen that the most effective HIV prevention efforts have employed a combination approach, customized for the specific settings and populations. Providing women with a variety of prevention options will likely lead to the most effective prevention for the most women in the widest range of settings. Such a strategy has been effective in addressing women's needs for contraception. Over the next decade, following new WHO guidelines, we will see an increase in the number of people starting ART at higher CD4 cell counts. This will lead to decreased HIV transmission; however, there will continue to be a pressing need for women-controlled prevention methods as not all male partners will be virally suppressed. The sobering observations of the challenges in many settings of achieving high levels of coverage in the various steps of the HIV prevention and treatment cascade will require new, more effective methods for HIV-negative women. Moreover, there continues to be a pressing need to increase levels of HIV testing to identify HIV-positive persons, including those with acute infection.

On-going and planned studies of new ARV-based prevention methods hold the promise of offering women highly effective prevention that can substantially limit HIV transmission. Studies to date have not identified sex differences in the levels of HIV protection provided by ARV-based methods; the overriding factor for both women and men is the level of adherence to products. It will be important to enhance our understanding of women's perceptions of risk and the complex behavioural dynamics that influence adherence to complement the development of new biologic products. On-going and planned PrEP demonstration studies will add greatly to our understanding of how ARV-based products will add to our HIV prevention tool kit (avac.org) [47]. The technical advances remain an important first step but are no magic bullets. We need to enhance our understanding of the bio-behavioural nexus, and demonstration projects will help identify appropriate target populations, acceptability of products, demand creation strategies and product use in non-trial settings.

## References

[1] Joint United Nations Programme on HIV/AIDS (UNAIDS) (2013). Global Report: UNAIDS report on the global AIDS epidemic 2013.

[2] Abdool Karim Q, Abdool Karim SS, Frohlich JA, Grobler AC, Baxter C, Mansoor LE (2010). Effectiveness and safety of tenofovir gel, an antiretroviral microbicide, for the prevention of HIV infection in women. Science.

[3] Van Damme L, Corneli A, Ahmed K, Agot K, Lombaard J, Kapiga S (2012). Preexposure prophylaxis for HIV infection among African women. N Engl J Med.

[4] Marrazzo J, Ramjee G, Nair G, Palanee T, Mkhize B, Nakabilito C Pre-exposure prophylaxis for HIV in women: daily oral tenofovir, oral tenofovir/emtriciabine, or vaginal tenofovir gel in the VOICE study (MTN 003).

[5] Grant RM, Lama JR, Anderson PL, McMahan V, Liu AY, Vargas L (2010). Preexposure chemoprophylaxis for HIV prevention in men who have sex with men. N Engl J Med.

[6] Baeten JM, Donnell D, Ndase P, Mugo NR, Campbell JD, Wangisi J (2012). Antiretroviral prophylaxis for HIV prevention in heterosexual men and women. N Engl J Med.

[7] Thigpen MC, Kebaabetswe PM, Paxton LA, Smith DK, Rose CE, Segolodi TM (2012). Antiretroviral preexposure prophylaxis for heterosexual HIV transmission in Botswana. N Engl J Med.

[8] Choopanya K, Martin M, Suntharasamai P, Sangkum U, Mock PA, Leethochawalit M (2013). Antiretroviral prophylaxis for HIV infection in injecting drug users in Bangkok, Thailand (the Bangkok Tenofovir Study): a randomised, double-blind, placebo-controlled phase 3 trial. Lancet.

[9] Cohen MS, Chen YQ, McCauley M, Gamble T, Hosseinipour MC, Kumarasamy N (2011). Prevention of HIV-1 infection with early antiretroviral therapy. N Engl J Med.

[10] Grant D, Marshall K (2013). Next steps for PrEP: getting a proven prevention option to the people who need it.

[11] Hendrix CW (2013). Exploring concentration response in HIV pre-exposure prophylaxis to optimize clinical care and trial design. Cell.

[12] Donnell D, Baeten JM, Bumpus NN, Brantley J, Bangsberg DR, Haberer JE (2014). HIV protective efficacy and correlates of tenofovir blood concentrations in a clinical trial of PrEP for HIV prevention. J Acquir Immune Defic Syndr.

[13] Abdool Karim SS, Kashuba AD, Werner L, Abdool Karim QA (2011). Drug concentrations after topical and oral antiretroviral pre-exposure prophylaxis: implications for HIV prevention in women. Lancet.

[14] van der Straten A, Brown E, Marrazzo J, Chirenje Z, Gomez K, Piper J Divergent adherence estimates with pharmacokinetic and behavioral measures in VOICE (MTN003).

[15] Corneli A, Perry B, Agot K, Ahmed K, McKenna K, Malamatsho F FEM-PrEP: participant explanations for non-adherence.

[16] Baeten JM, Celum C (2013). Antiretroviral preexposure prophylaxis for HIV prevention. N Engl J Med.

[17] Solomon MM, Lama JR, Glidden DV, Mulligan K, McMahan V, Liu AY (2014). Changes in renal function associated with oral emtricitabine/tenofovir disoproxil fumarate use for HIV pre-exposure prophylaxis. AIDS.

[18] Murnane PM, Celum C, Mugo N, Campbell JD, Donnell D, Bukusi E (2013). Efficacy of preexposure prophylaxis for HIV-1 prevention among high-risk heterosexuals: subgroup analyses from a randomized trial. AIDS.

[19] Haberer JE, Baeten JM, Campbell J, Wangisi J, Katabira E, Ronald A (2013). Adherence to antiretroviral prophylaxis for HIV prevention: a substudy cohort within a clinical trial of serodiscordant couples in East Africa. PLoS Med.

[20] Corneli A, Deese J, Wang M, Taylor D, Ahmed K, Agot K (2014). FEM-PrEP: adherence patterns and factors associated with adherence to a daily, oral study product for pre-exposure prophylaxis. J Acquir Immune Defic Syndr.

[21] Gengiah TN, Moosa A, Naidoo A, Mansoor LE (2014). Adherence challenges with drugs for pre-exposure prophylaxis to prevent HIV infection. Int J Clin Pharm.

[22] Wong C, Parker C, Ahmed K, Agot K, Skhosana J, Odhiambo J Participant motivation for enrolling and continuing in the FEM-PrEP HIV prevention clinical trial.

[23] Abbas UL, Anderson RM, Mellors JW (2007). Potential impact of antiretroviral chemoprophylaxis on HIV-1 transmission in resource-limited settings. PLoS One.

[24] Williams BG, Abdool Karim SS, Karim QA, Gouws E (2011). Epidemiological impact of tenofovir gel on the HIV epidemic in South Africa. J Acquir Immune Defic Syndr.

[25] Pretorius C, Stover J, Bollinger L, Bacaer N, Williams B (2010). Evaluating the cost-effectiveness of pre-exposure prophylaxis (PrEP) and its impact on HIV-1 transmission in South Africa. PLoS One.

[26] Walensky RP, Park JE, Wood R, Freedberg KA, Scott CA, Bekker LG (2012). The cost-effectiveness of pre-exposure prophylaxis for HIV infection in South African women. Clin Infect Dis.

[27] Kashuba AD, Patterson KB, Dumond JB, Cohen MS (2012). Pre-exposure prophylaxis for HIV prevention: how to predict success. Lancet.

[28] Kibengo FM, Ruzagira E, Katende D, Bwanika AN, Bahemuka U, Haberer JE (2013). Safety, adherence and acceptability of intermittent tenofovir/emtricitabine as HIV pre-exposure prophylaxis (PrEP) among HIV-uninfected Ugandan volunteers living in HIV-serodiscordant relationships: a randomized, clinical trial. PLoS One.

[29] Chen B, Panther L, Hoesley C, Hendrix C, van der Straten A, Husnik M Safety and pharmacokinetics/pharmacodynamics of dapivirine and maraviroc vaginal rings.

[30] AVAC Report 2013: research & reality (2013).

[31] Bunge K, Dezzutti C, Macio I, Hendrix C, Lisa C, Rohan L FAME-02: a phase I trial to assess safety, PK, and PD of gel and film formulations of dapivirine.

[32] Friend DR, Clark JT, Kiser PF, Clark MR (2013). Multipurpose prevention technologies: products in development. Antiviral Res.

[33] Spreen WR, Margolis DA, Pottage JC (2013). Long-acting injectable antiretrovirals for HIV treatment and prevention. Curr Opin HIV AIDS.

[34] Boffito M, Jackson A, Owen A, Becker S (2014). New approaches to antiretroviral drug delivery: challenges and opportunities associated with the use of long-acting injectable agents. Drugs.

[35] Jackson A, Else LJ, Higgs C, Karolia Z, Khoo S, Back D Rilpivirine with darunavir/ritonavir: pharmacokinetics & safety in HIV therapy-naïve patients.

[36] Radzio J, Spreen W, Yueh Y, Mitchell J, Jenkins L, Garcia-Lerma J Monthly GSK744 long-acting injections protect macaques against repeated vaginal SHIV exposures.

[37] Andrews C, Spreen W, Yueh Y, Gettie A, Russell-Lodrigue K, Mohri H Correlating GSK1265744 plasma levels to prevention of rectal SHIV transmission in macaques.

[38] Pace CS, Song R, Ochsenbauer C, Andrews CD, Franco D, Yu J (2013). Bispecific antibodies directed to CD4 domain 2 and HIV envelope exhibit exceptional breadth and picomolar potency against HIV-1. Proc Natl Acad Sci USA.

[39] Pace CS, Fordyce MW, Franco D, Kao CY, Seaman MS, Ho DD (2013). Anti-CD4 monoclonal antibody ibalizumab exhibits breadth and potency against HIV-1, with natural resistance mediated by the loss of a V5 glycan in envelope. J Acquir Immune Defic Syndr.

[40] Hallett TB, Baeten JM, Heffron R, Barnabas R, de Bruyn G, Cremin I (2011). Optimal uses of antiretrovirals for prevention in HIV-1 serodiscordant heterosexual couples in South Africa: a modelling study. PLoS Med.

[41] El-Sadr WM, Coburn BJ, Blower S (2011). Modeling the impact on the HIV epidemic of treating discordant couples with antiretrovirals to prevent transmission. AIDS.

[42] Delva W, Eaton JW, Meng F, Fraser C, White RG, Vickerman P (2012). HIV treatment as prevention: optimising the impact of expanded HIV treatment programmes. PLoS Med.

[43] The HIV Modelling Consortium Treatment as Prevention Editorial Writing Group (2012). HIV treatment as prevention: models, data, and questions – towards evidence-based decision-making. PLoS Med.

[44] World Health Organization (WHO) (2013). Consolidated guidelines on the use of antiretroviral drugs for treating and preventing HIV infection: recommendations for a public health approach.

[45] Phillips A MSM in the UK: prevention effects of ART in perspective.

[46] Tanser F, Barnighausen T, Grapsa E, Zaidi J, Newell ML (2013). High coverage of ART associated with decline in risk of HIV acquisition in rural KwaZulu-Natal, South Africa. Science.

[47] Heffron R, Celum C, Mugo N, Katabira E, Bukusi E, Tumwesigye E High initiation of PrEP and ART in a demonstration project among African HIV-discordant couples.

# Sub article: Engaging male partners in women's microbicide use: evidence from clinical trials and implications for future research and microbicide introduction

**DOI:** 10.7448/IAS.17.3.19159

**Published:** 2014-09-08

**Authors:** Michele Lanham, Rose Wilcher, Elizabeth T Montgomery, Robert Pool, Sidney Schuler, Rachel Lenzi, Barbara Friedland

**Affiliations:** 1 Social and Behavioral Health Sciences, FHI 360 Durham, NC, USA; 2 Research Utilization, FHI 360 Durham, NC, USA; 3 Women's Global Health Imperative, RTI International, San Francisco Project Office San Francisco, CA, USA; 4 Centre for Social Science and Global Health, University of Amsterdam, Amsterdam, The Netherlands; 5 HIV and AIDS Program, Population Council New York, NY, USA

**Keywords:** Microbicides, HIV, qualitative research, partner communication, gender relations

## Abstract

**Introduction:**

Constructively engaging male partners in women-centred health programs such as family planning and prevention of mother-to-child HIV transmission has resulted in both improved health outcomes and stronger relationships. Concerted efforts to engage men in microbicide use could make it easier for women to access and use microbicides in the future. This paper synthesizes findings from studies that investigated men's role in their partners’ microbicide use during clinical trials to inform recommendations for male engagement in women's microbicide use.

**Methods:**

We conducted primary and secondary analyses of data from six qualitative studies implemented in conjunction with microbicide clinical trials in South Africa, Kenya, and Tanzania. The analyses included data from 535 interviews and 107 focus groups with trial participants, male partners, and community members to answer research questions on partner communication about microbicides, men's role in women's microbicide use, and potential strategies for engaging men in future microbicide introduction. We synthesized the findings across the studies and developed recommendations.

**Results:**

The majority of women in steady partnerships wanted agreement from their partners to use microbicides. Women used various strategies to obtain their agreement, including using the product for a while before telling their partners, giving men information gradually, and continuing to bring up microbicides until resistant partners acquiesced. Among men who were aware their partners were participating in a trial and using microbicides, involvement ranged from opposition to agreement/non-interference to active support. Both men and women expressed a desire for men to have access to information about microbicides and to be able to talk with a healthcare provider about microbicides.

**Conclusions:**

We recommend counselling women on whether and how to involve their partners including strategies for gaining partner approval; providing couples’ counselling on microbicides so men have the opportunity to talk with providers; and targeting men with community education and mass media to increase their awareness and acceptance of microbicides. These strategies should be tested in microbicide trials, open-label studies, and demonstration projects to identify effective male engagement approaches to include in eventual microbicide introduction. Efforts to engage men must take care not to diminish women's agency to decide whether to use the product and inform their partners.

## Introduction

Microbicides were originally conceived of as a female-controlled HIV prevention method [1]. Subsequent research has found that although many women appreciate a product they can use without a partner's knowledge [2–7], microbicide trial participants typically talked with their steady partners about the product [3, 6, 8–12]. Moreover, male partners’ awareness and acceptance of product use influenced trial participants’ product acceptance and self-reported adherence [7, 13–16]. Male partner influence seems to differ by partner type, however. In several trials, women reported better adherence and greater ease using products discretely with casual, transactional, and non-cohabitating partners compared to steady partners [11, 16–18]. Involving male partners in microbicide use can also affect relationship quality; some trial participants reported that involving their partners improved partner communication and increased shared responsibility for HIV protection [13, 16, 19–21].

After more than two decades of testing microbicides in clinical trials, the CAPRISA 004 trial of 1% tenofovir gel provided proof of concept that microbicides can reduce women's HIV risk [22]. Ongoing clinical trials, including the FACTS 001 trial of 1% tenofovir gel and the MTN-020 and IPM 027 trials of the dapivirine ring, could provide sufficient evidence to support product licensure and introduction. Male engagement in family planning and prevention of mother-to-child HIV transmission (PMTCT) has been shown to improve acceptability, uptake, adherence, and health outcomes [23–27]. Considering whether and how to engage male partners at the outset of microbicide introduction could help maximize the potential for HIV prevention.

This paper synthesizes findings from microbicide studies that investigated men's role in their partners’ microbicide use during clinical trials in sub-Saharan Africa. Clinical trials are a unique environment, so men's roles may differ in the context of “real world” use. Nevertheless, the data provide a starting point for identifying effective ways to engage male partners in future research and eventual microbicide introduction.

## Methods

In January 2013, FHI 360 convened a meeting of microbicides experts and male engagement experts to develop the following research questions to inform recommendations for engaging male partners in microbicide introduction:

1. What existing gender norms could facilitate or hinder constructive male engagement in microbicide use?
2. What did women in the trials tell their male partners about the trial and/or the product, when did they tell them, and why?
3. How have men been involved in women's use of microbicides?
4. What information do men want about microbicides?
5. What are the best ways to engage men in women's microbicide use?
6. What are men's barriers to accompanying their partners to the clinic for microbicide services?

To answer the research questions, FHI 360 and the Kenya Medical Research Institute collected qualitative data in Kisumu, Kenya, where three microbicide trials had been conducted. FHI 360 also partnered with social scientists who had previously collected qualitative data about male engagement during microbicide trials.

This paper includes data from six qualitative studies (described in detail in Table 1). The trials all tested microbicide gel formulations, except IPM 015, which tested a microbicide ring. The VOICE trial tested oral pre-exposure prophylaxis as well as daily 1% tenofovir gel, and the mock clinical trial tested a proxy gel and proxy oral pill. All studies received approval from local ethics committees, and informed consent was obtained from each study participant.

**Table 1:** Qualitative data included in the analysis

Clinical trial	Qualitative data included in analysis
Trial: MDP301 [28] Phase: III Product: PRO2000 vaginal gel Length of product use: 12 months (24 in Uganda) Years: 2005–2009 Sites: 13 sites in South Africa, Tanzania, Uganda, and Zambia Participants: 9,385 sexually active women, aged 18 years or older (≥16 years in Tanzania and Uganda)	Study name: MDP301 [10, 29–31] Main focus: Social science sub-study to assess the accuracy of behavioural and adherence data, acceptability of the gel and the trial procedures, and understanding of the trial and the consent procedure Years: 2005–2009 Sites: 3 sites in Johannesburg, Durban, rural KwaZulu-Natal, South Africa Ethics approvals: Witwatersrand University Human Research Ethics Committee; University of KwaZulu-Natal Biomedical Research Ethics Committee; The Medicines Control Council of South Africa Participants: – 154 individual in-depth interviews (IDIs) with 90 men and women (45 couples). These women shared information about the trial and involved their partners from early on – 60 IDIs with 30 women who did not immediately inform their partners – 31 focus group discussions (FGDs) with trial participants, 18 FGDs with women in the community and 18 FGDs with men in the community Methods for secondary analysis: NVivo software was used to re-analyse IDIs and FGDs, through additional coding and extraction of themes relevant to male engagement

Trial: Carraguard Phase 3 Trial [32] Phase: III Product: Carraguard vaginal gel Length of product use: 9–24 months Years: 2004–2007 Sites: 3 sites in South Africa (Gugulethu, Soshanguve and Isipingo) Participants: 6,202 women, aged 16 and older	Study name: Evaluation of the Informed Consent Process in the Phase 3 Study of the Efficacy and Safety of the Microbicide Carraguard^®^ in Preventing HIV Seroconversion in Women [33–37] Main focus: Informed consent Years: 2006–2007 Sites: Gugulethu and Soshanguve, South Africa Ethics approvals: Population Council Institutional Review Board; Ethics Committee of the University of Cape Town; the Research Ethics and Publication Committee of the University of Limpopo, Medunsa Campus Participants: – 103 IDIs with trial participants – 5 FGDs with trial participants (*n*=29) – 1 mixed gender FGD (*n*=3 trial participants, 2 male partners) – 2 FGDs with male partners (*n*=8)
	Study name: Microbicides Acceptability: A Qualitative Study to Explore Social and Cultural Norms, Interpersonal Relations and Product Attributes [38, 39] Main focus: Sexual norms and gender roles affecting microbicide acceptability Years: 2006–2007 Sites: all three sites Ethics approvals: Population Council Institutional Review Board; Ethics Committee of the University of Cape Town; the Research Ethics and Publication Committee of the University of Limpopo, Medunsa Campus; the Biomedical Research Ethics Committee, University of Kwa-Zulu Natal Participants: – 62 IDIs with trial participants – 14 FGDs with trial participants (*n*=97) – 2 FGDs with male partners (*n*=13) – 3 IDIs with male partners Methods for secondary analysis: Thematic analysis of the previously coded data was conducted for codes most pertinent to the research questions

Trial: MTN-003 “VOICE” [40] Phase: IIb Product: Vaginal 1% tenofovir gel, oral tenofovir, oral tenofovir/emtricitabine	Study name: MTN-003C “VOICE-C” [41] Main focus: Household and community-level factors associated with study product adherence in VOICE Years: 2010–2012
Length of product use: Up to 36 months Years: 2008–2012 Sites: South Africa (3 sites), Zimbabwe, and Uganda Participants: 5029 women, half were aged 18–24	Site: Johannesburg, South Africa Ethics approvals: Office of Research Protection Institutional Review Board, RTI International; Human Research Ethics Committee of the University of the Witwatersrand Participants: – 22 male partners in 14 IDIs and 2 FGDs (*n*=8) – 102 randomly-selected female VOICE participants in IDIs (*n*=41), ethnographic interviews (*n*=21) and 7 FGDs (*n*=40) Methods for secondary analysis: Thematic analysis of coded transcripts from all IDI, EI and FGD data from male partners and female study participants described above

Trial: IPM 014A Phase: I/II Product: Dapivirine vaginal gel 4759, 0.05% 2.5G Length of product use: 6 weeks Years: 2009 Sites: Kenya, Malawi, Rwanda and South Africa Participants: Approximately 320 women, aged 18–40 Trial: IPM 015 Phase: I/II Product: Dapivirine vaginal ring Length of product use: 12 weeks Years: 2010 Sites: Kenya, Malawi, Rwanda, South Africa and Tanzania Participants: Approximately 280 women, aged 18–40	Study name: Male Engagement in Microbicides Main focus: Identifying strategies for engaging men in future trials, open-label studies, demonstration projects, and microbicide introduction so they will support their female partners in using microbicide products for HIV prevention or, at least, to minimize men's interference in women's microbicide use Years: 2013 Site: Kisumu, Kenya Ethics approvals: Kenya Medical Research Institute Ethical Review Committee; FHI 360's Protection of Human Subjects Committee Participants: – 30 IDIs with former female trial participants – 25 IDIs with women who were not trial participants – 14 IDIs with men who were partners of trial participants at the time of the trial – 29 IDIs with men who were not partners of trial participants Methods for analysis: Thematic analysis of IDIs using NVivo 9 software. The saliency of themes was assessed via frequencies, degrees of emphasis and elaboration, co-occurrences, and contrasts of themes across interviews, and compared between participant groups
Trial: MTN-004/ VivaGel [42] Phase: I Products: VivaGel^®^ (SPL7013 gel) vaginal gel Length of product use: 14 days Years: 2006–2007 Sites: United States, Kenya Participants: 54 sexually active women, aged 18 to 24	

Trial: Adolescents and Microbicide Clinical Trials: Assessing the Opportunities and Challenges of Participation [43–46] Phase: Mock clinical trial Product: Proxy vaginal gel (Pre-Seed lubricant) and proxy oral pill (Vitacap multivitamin) Length of product use: optional 0–2 months Years: 2011–2013 Sites: Tanzania Participants: 135 sexually active adolescents and young women, aged 15–21	Study name: Adolescents and Microbicide Clinical Trials: Assessing the Opportunities and Challenges of Participation [45, 47–49] Main focus: Socio-cultural factors that hinder young women's participation in topical or oral microbicide trials Years: 2010–2011 Site: Dar es Salaam, Tanzania Ethics approvals: Muhimbili University of Health and Allied Sciences; National Institute for Medical Research (Tanzania); FHI 360's Protection of Human Subjects Committee Participants: – 3 FGDs with mothers of adolescents in the community (*n*=25) – 2 FGDs with fathers of adolescents in the community (*n*=18) – 2 FGDs with unmarried male partners of adolescents in the community (*n*=14) Methods for secondary analysis: Thematic analysis of coded excerpts from all FGD data from male partners, fathers, and mothers of adolescent girls

Social scientists from each study conducted primary or secondary analyses of their data and summarized the findings to answer the research questions, and FHI 360 staff synthesized the findings across the six studies. In November 2013, the social scientists and additional experts met to discuss each study's results and identify central themes and programmatic implications of the data.

## Results

### Gender norms

Prevailing gender norms about sexuality and complex relationship dynamics affected how women addressed their trial participation and microbicide use with male partners. Couples were more likely to discuss HIV risk, get tested, and use condoms at the beginning of a relationship. Such discussions were considered less acceptable later in a relationship. For some men, a partner's use of microbicides was a sign that she suspected he was unfaithful or that she had outside partners. Women found ways to work within the existing patriarchal gender relations, such as negotiating microbicide use without openly challenging male authority or voicing suspicions of infidelity. In some cases men and women implied that men needed a public appearance of authority, but were more willing to be flexible within the privacy of their relationships.

### Partner communication

Women and men agreed that, ideally, women would discuss microbicide use with their steady partners. The majority of women wanted agreement from their steady partners to use microbicides, though many women and some men thought it was ultimately the woman's decision. Trial participants typically decided whether to discuss microbicide use with their partners based on: 1) the nature of their relationship, 2) the partner's temperament, and 3) evaluation of how the partner might react if told in advance versus finding out the product had been used without his knowledge. Though microbicide use and trial participation are distinct topics, women usually did not distinguish between the two when communicating with partners.

Women gave two main reasons for not discussing microbicide use with partners. They either feared a negative reaction, ranging from mere objection to violence, or thought their partners did not need to know, particularly in casual relationships. Women who feared a negative reaction often thought the benefit of using microbicides was worth the risk of their partners later discovering their covert use. Contrary to the widely reported belief that discreet use would cause problems, some men who were unaware of their partner's microbicide use were not upset when they found out about it.

Trial participants who decided to discuss microbicides with their partners did so for emotional, logistical, and strategic reasons. Many women said they wanted to promote an open, trusting relationship or prevent a disagreement or breakup. Others wanted to gain the partner's support in case they later experienced side effects or other problems. Some felt it was “the right thing to do,” because their partners would also be exposed to an experimental product, and others thought it would be difficult to explain the sudden need to use condoms, frequent visits to the clinic, and (for gel users) change in lubrication during sex. They also said it would be challenging to hide the applicators and insert the gel. Women reported a stronger sense of desire or obligation to discuss microbicide use in steady relationships than casual relationships. They also said that the logistics of trial participation and product use were more difficult to keep secret from a steady partner.

The vast majority of women in MDP301 and many women in the other studies told their partners about their microbicide use early during trial participation. The follow-up study in Kenya was an exception; one-third of the women interviewed said they had not told their partners. This may have been because they used the product for a shorter period of time compared to women in the other studies, making it less necessary to inform their partners.

When male partners were initially resistant to using microbicides, women used a number of strategies to obtain their agreement. Some women used the product for a while before telling their partners about it. If a partner objected, the woman reminded him that he had not noticed a difference, or in some cases that he had noticed but was enjoying their sexual experience. This was particularly the case with microbicide gels; many couples found the lubrication from the gel increased pleasure or at least decreased pain. Other women gave their partners incomplete information (e.g. not mentioning that the microbicide contained an antiretroviral drug) or misinformation (e.g. saying it was a family planning method). Other strategies included giving men information gradually or continuing to bring up microbicides until resistant partners acquiesced. Some women made their partners feel that they were making the decision, playing into the gender norm that men dominate decision-making. When partners did not agree on microbicide use, some women succeeded in using without their knowledge. See Table 2 for illustrative quotes about partner communication.

**Table 2:** Select themes and illustrative quotes

Theme	Illustrative quotes
**Reasons women discussed microbicides with their partners**	
To promote an open, trusting relationship or to prevent a disagreement or breakup	*If we did not have an agreement before I started participating in the study, […] maybe we would have fought and I would have decided to quit the study. So I tell him about a lot of things before they happen because I don't like keeping secrets*. (VOICE participant, Johannesburg, South Africa)
To gain their partner's support in case they later experienced problems	*I thought I should tell them [partner and a friend] because maybe if I tell them […] and there are problems while I am participating at least they will know that I am in the trial. […] They can give me support*. (Carraguard participant, Soshanguve, South Africa)
Would be difficult to hide trial participation and microbicide use	*I had to because it was going to be difficult to do anything without telling him. He was going to be surprised if I start applying the gel and he does not know about it, it was not going to be possible*. (Carraguard participant, Soshanguve, South Africa)

**Reasons women did not discuss microbicides with their partners**	
Feared a negative reaction	*My partner is a difficult guy to deal with …. He's noisy, violent, and anything you tell him he disapproves; even if I left the house for a few minutes he would think that I've gone to see other men …. He would have beaten me up or chased me away …. I think it should be a secret*. (IPM 014A participant, Kisumu, Kenya)
Thought their partners did not need to know	*It just did not come to my mind to tell him because I think it is too early in our relationship. I will tell him later*. (VOICE participant, Johannesburg, South Africa)

**Strategies used with reluctant partners**	
Used the product for a while and then told partner about it	*I told him about it and he refused to allow me to use the gel. […] I said to him, ‘it's fine’ … [but] I used the gel without telling him and we had sex all the time. […] He did not feel it. And after two months using it in secret I talked to him again about it. But I did not tell him I was using it in secret. Then he said that if I used the gel he would not have sex with me. I asked him why. He said that he did not know if sex was going to be the same or not. Then I asked him how he felt about our sex and he said that it was okay. I then told him I had been using the gel, and he said that I could continue to use it*. (MDP301 participant, South Africa)
Gave partners incomplete information or misinformation	*I just told him that I was participating in a study*. (IPM 015 participant, Kisumu, Kenya) *I just told him we were studying something on family planning*. (IPM 015 participant, Kisumu, Kenya)
Gave partner information gradually or continuing to bring up microbicides with their resistant partner until they acquiesced	*I told my husband that at Empilisweni [study clinic] it is said there is a gel that is being used that helps prevent HIV; my husband refused that I come here …. When I came home I told him that I had been here and I watched a video and I'm going to come again, and he wasn't agreeable, resisting, and I came again …. and then on another day he gave me permission*. (Carraguard participant, Gugulethu, South Africa)

**Continuum of male engagement in women's microbicide use**	
Opposition	*Personally if you ask me I will not allow her because of one reason, I think she can be like a slave; every end of month she will be attending clinic. Also, she will not be allowed to carry pregnancy; it means we will postpone getting children because of that study*. (Male partner of adolescent woman, Dar es Salaam, Tanzania)
Agreement/non-interference	*… he had the option of stopping me from using the product. He will tell me not to participate in anything that he doesn't like. I went to the clinic and he knew about it. He never stopped me. That means he supported my decision*. (IPM 015 participant, Kisumu, Kenya) *When she comes here she tells me that she is coming here [study clinic], I do not have a problem and I allow her to come here*. (Male partner of VOICE participant, Johannesburg, South Africa) *I have told her one thing ‘if you are happy and it is treating you well [not reacting negatively to you], I do not have a problem, and I will not follow you or accompany you when you go to the study because to a certain extent [if I do that] you may think that I do not trust you.’* (Male partner of VOICE participant, Johannesburg, South Africa)
Active support	*[My partner] was very receptive. He even accompanied me to the clinic when he was off duty. Sometimes he even ask me before we have sex that ‘are you not applying the gel, please don't forget it.’* (Carraguard participant, Soshanguve, South Africa) *I used to give her money for clinic visits and took care of our children when she went to the clinic*. (IPM 014A participant, Kisumu, Kenya)

### Men's roles

Among men who knew about their partner's participation in a trial and use of microbicides, involvement ranged from opposition to agreement/non-interference to active support (See Figure 1, Table 2). Most men fell in the “agreement/non-interference” part of the continuum. This may not be representative of all trial participants’ experiences, because women who encountered opposition from their partners may have discontinued trial participation or elected not to participate in interviews.

**Figure 1:** Continuum of male partner involvement in microbicide use.

#### Opposition

Some men were opposed to their partners participating in the trial and/or using microbicides because they had concerns about product safety, potential partner infidelity, and the researchers’ intent. Some men were resistant because they lacked knowledge about the product or trial, and some did not want their partners participating in something beyond their control or understanding. Their opposition ranged from voicing unease or uncertainty to outright refusal.

#### Agreement/non-interference

Some men gave their partners permission to use microbicides. In other cases, male partners’ “agreement” was tacit; they did not give express permission, but knew their partners were using microbicides and did not interfere.

#### Active support

Some men provided their partners with instrumental or emotional support to participate in a trial and use microbicides. They reminded their partners to use the gel or in some cases inserted it for them as part of foreplay. They helped their partners attend clinic visits by reminding them of their appointments, occasionally accompanying them, or giving them transport money. The studies did not quantify the proportion of men who provided these more active forms of support, though it seems they were in the minority. A larger number of men were reported to have adapted their sexual practices to meet the trial requirements of regular condom and microbicide use.

Men's involvement often varied over time. For example, some men were opposed to microbicide use at first, but after their partners convinced them to try it, they liked it and agreed to the product's use. Other men were generally supportive of microbicide use but occasionally suggested sex without the gel.

### Other considerations for engaging male partners

Periodic HIV counselling and testing (HCT) is required during trial participation and will be part of the service delivery package for any antiretroviral-based microbicide introduced in the public sector. Trial participation gave women a reason to discuss HIV testing and results with their partners. Indeed, men equated clinical trials with access to HIV testing and results, which elicited two common responses. A number of men were pleased their partners were getting tested regularly and being found to be HIV negative. Particularly in the studies in South Africa, men were generally unwilling to get tested themselves and used their partner's HIV-negative status as a proxy for their own status. Men in the study in Kenya were generally more open to HIV testing. The second common reaction was fear. Some men did not want to go to the trial clinic with their partners because they feared being forced to get tested.

Men and women expressed a desire for men to have access to information about microbicides. Men wanted to know about the safety and side effects of the product, whether microbicides prevent pregnancy, whether microbicides protect men from HIV, and whether they affect fertility or the sexual experience. Some also had questions (and suspicions) about the clinical trial process, including why it was being tested by “whites” or “foreigners” on African women.

Some women and men said it would be helpful if male partners could talk directly with a healthcare provider about microbicides, because providers carry authority and could legitimize the product and the research. Some felt their own ability to convey information in the most accurate and persuasive way was limited and wanted assistance from providers.

Several of the studies experienced challenges recruiting men for interviews and focus groups. Men were often unavailable because they were working. Others were hesitant to go to the study clinic because they were afraid it would involve HIV testing or thought “the clinic was meant for women only.” Some studies had more success recruiting men if they conducted interviews and focus groups outside of the clinic.

## Discussion

Findings from these six qualitative studies confirm that male partners play an important role in women's microbicide use and provide insights for engaging men in future microbicide studies and product introduction. Promoting women's agency to decide whether to use microbicides and inform their partners is of paramount importance [50]. Not only is each woman best equipped to understand whether it is safe and/or will be productive to discuss microbicide use with her partner, she is also likely to be most skilled in introducing the subject and ultimately gaining his support. Enabling women to make decisions about microbicide use and providing the skills and enabling environment to act on them will help ensure that microbicides, if effective, are the game-changer for women's HIV prevention they were intended to be.

We recommend the primary goal of male engagement in women's microbicide use be to promote women's ability to access and use the product. To accomplish the primary goal, we recommend 1) providing support to women through counselling to decide whether and how to involve their partners and 2) sharing basic information about microbicides with men through couples’ counselling, community education, and mass media to increase their awareness and acceptance of the product (see Table 3).

**Table 3:** Recommended strategies for engaging male partners

Goal	Objectives	Strategies	Rationale	Considerations
Increase women's ability to access and use microbicides	Support women to decide whether/how to talk with partner about microbicides	Counselling for women	Context of trial participation provided women supportive environment for communicating with partner; women will need support in real world introduction	Include: – Women's right to decide whether to talk with her partner – Strategies for communicating with partner to gain his approval – Strategies for using microbicides without partner's knowledge – Written materials women can share with partner
	Increase men's awareness and acceptance of microbicides to create an enabling environment for women to discuss and negotiate microbicide use	Couples counselling on microbicides	Women and men expressed a desire for men to be able to talk with health providers	Men hesitant to attend clinics because: – Feared HIV testing – Some clinics viewed as women's spaces – Scheduling conflicts To address these barriers: – Consider offering microbicides at clinics with services for men and women – Providers offer to call partners or visit homes
		Community education	May be more effective than reaching men through clinics	– Provide general information on benefits, safety – Target spaces where men congregate
		Mass media	May be more effective than reaching men through clinics	– Feature health providers, who are viewed as reliable sources of information – Promote benefits, safety – Show steady couples using microbicides

Some of these male engagement strategies – including counselling women, inviting male partners to the study clinic, offering couples counselling and informational materials, and doing community outreach to men – are currently being implemented at some microbicide trial sites to improve participant recruitment, retention, and adherence. However, the positive and negative effects of these approaches have not been rigorously evaluated. Consistent product use, which is crucial to effectiveness, has been lower than anticipated in trials [22, 32, 51], and may be improved through strategies to increase or improve positive male engagement. The male engagement strategies we recommend should be tested as part of a multi-pronged adherence support program. Their effect on partner communication, relationship quality and intimate partner violence should also be measured.

### Limitations

There are several limitations to our synthesis. Women participating in clinical trials may differ from women in the general population; they may be more empowered or more motivated to prevent HIV or may have partners who are more open to their participation, among other differences. Moreover, some of the qualitative studies experienced difficulty recruiting men; those who agreed were likely more supportive partners and therefore not representative of all male partners of trial participants. It is likely that some women did not participate in microbicide trials because of partner opposition, so such couples would not be represented in the studies. Clinical trials also provide a supportive context for women to discuss microbicides with their partners. Specifically, trial requirements give women leverage to negotiate condom and microbicide use with partners. In addition, women receive intensive counselling and support from trial staff. These conditions may be difficult to replicate in real world microbicide introduction, where providers have less time and fewer resources. Partner engagement may differ outside of clinical trials; women may be more or less likely to talk with their partners when they are using a product with known effectiveness and when condom use is recommended but not required.

Most of the studies focused on microbicide gels. Partner interaction may differ somewhat for other microbicide formulations, such as rings, films, suppositories, and injectables. For example, women using a gel may be more likely to tell their partners about their microbicide use compared to women using a ring, because they anticipate the increased lubrication caused by the gel will be noticed by partners or that it will be difficult to insert gel regularly without a partner's knowledge. Also, women may find it easier to talk with partners about use of a multipurpose microbicide product that protects against both HIV and pregnancy, because contraceptive use may be more acceptable than using HIV prevention methods in steady relationships.

Although our findings are not necessarily generalizable to microbicide use outside of the trial context or to different microbicide formulations, this is the first study to synthesize data on partner interactions in multiple clinical trials, and our recommendations provide a starting point for future male engagement efforts in microbicide research and programs.

## Conclusions

Microbicides by themselves will not alter the underlying gender norms that put women at risk of HIV. If proven effective, they may give women some level of increased control over HIV prevention, but many women – particularly those in steady relationships – may find it easier to access and adhere to a microbicide product if their male partners are supportive of its use. The strategies we recommend – based on data from multiple sites in three countries – could increase men's awareness and acceptance of microbicides, thereby potentially enhancing women's ability to access and use microbicides. While the strategies will need to be tailored to individual communities and specific microbicide formulations, they must consistently preserve women's agency to decide whether to use the product and inform their partners. They should be tested in microbicide trials, open-label studies, and demonstration projects to determine their impact on women's microbicide use and gender relations and identify effective male engagement strategies to include in eventual microbicide introduction.

## References

[1] Stein ZA (1990). HIV prevention: the need for methods women can use. Am J Public Health.

[2] Whitehead SJ, McLean C, Chaikummao S, Braunstein S, Utaivoravit W, van de Wijgert JH (2011). Acceptability of Carraguard vaginal microbicide gel among HIV-infected women in Chiang Rai, Thailand. PLoS One.

[3] Pool R, Whitworth JA, Green G, Mbonye AK, Harrison S, Wilkinson J (2000). An acceptability study of female-controlled methods of protection against HIV and STDs in south-western Uganda. Int J STD & AIDS.

[4] Hoffman S, Morrow KM, Mantell JE, Rosen RK, Carballo-Dieguez A, Gai F (2010). Covert use, vaginal lubrication, and sexual pleasure: a qualitative study of urban U.S. Women in a vaginal microbicide clinical trial. Arch Sex Behav.

[5] Rosen RK, Morrow KM, Carballo-Dieguez A, Mantell JE, Hoffman S, Gai F (2008). Acceptability of tenofovir gel as a vaginal microbicide among women in a phase I trial: a mixed-methods study. J Women's Health.

[6] van der Straten A, Montgomery ET, Cheng H, Wegner L, Masenga G, von Mollendorf C (2012). High acceptability of a vaginal ring intended as a microbicide delivery method for HIV prevention in African women. AIDS Behav.

[7] Montgomery ET, Cheng H, van der Straten A, Chidanyika AC, Lince N, Blanchard K (2010). Acceptability and use of the diaphragm and Replens lubricant gel for HIV prevention in Southern Africa. AIDS Behav.

[8] Gafos M, Mzimela M, Sukazi S, Pool R, Montgomery C, Elford J (2010). Intravaginal insertion in KwaZulu-Natal: sexual practices and preferences in the context of microbicide gel use. Cult Health Sex.

[9] Green G, Pool R, Harrison S, Hart GJ, Wilkinson J, Nyanzi S (2001). Female control of sexuality: illusion or reality? Use of vaginal products in south west Uganda. Soc Sci Med.

[10] Montgomery CM, Gafos M, Lees S, Morar NS, Mweemba O, Ssali A (2010). Re-framing microbicide acceptability: findings from the MDP301 trial. Cult Health Sex.

[11] Sahin-Hodoglugil NN, van der Straten A, Cheng H, Montgomery ET, Kacanek D, Mtetwa S (2009). Degrees of disclosure: a study of women's covert use of the diaphragm in an HIV prevention trial in sub-Saharan Africa. Soc Sci Med.

[12] Woodsong C (2004). Covert use of topical microbicides: implications for acceptability and use. Int Fam Plan Perspect.

[13] Montgomery ET, van der Straten A, Chidanyika A, Chipato T, Jaffar S, Padian N (2011). The importance of male partner involvement for women's acceptability and adherence to female-initiated HIV prevention methods in Zimbabwe. AIDS Behav.

[14] Salter ML, Go VF, Celentano DD, Diener-West M, Nkhoma CM, Kumwenda N (2008). The role of men in women's acceptance of an intravaginal gel in a randomized clinical trial in Blantyre, Malawi: a qualitative and quantitative analysis. AIDS Care.

[15] Mngadi KT, Maarschalk S, Grobler AC, Mansoor LE, Frohlich JA, Madlala B (2014). Disclosure of microbicide gel use to sexual partners: influence on adherence in the CAPRISA 004 trial. AIDS Behav.

[16] Greene E, Batona G, Hallad J, Johnson S, Neema S, Tolley EE (2010). Acceptability and adherence of a candidate microbicide gel among high-risk women in Africa and India. Cult Health Sex.

[17] Muchomba FM, Gearing RE, Simoni JM, El-Bassel N (2012). State of the science of adherence in pre-exposure prophylaxis and microbicide trials. J Acquir Immune Defic Syndr.

[18] Woodsong C, MacQueen K, Amico KR, Friedland B, Gafos M, Mansoor L (2013). Microbicide clinical trial adherence: insights for introduction. J Int AIDS Soc.

[19] Woodsong C, Macqueen K, Namey E, Sahay S, Morar N, Mlingo M (2006). Women's autonomy and informed consent in microbicides clinical trials. J Empir Res Hum Res Ethics.

[20] Pistorius AG, van de Wijgert JH, Sebola M, Friedland B, Nagel E, Bokaba C (2004). Microbicide trials for preventing HIV/AIDS in South Africa: phase II trial participants’ experiences and psychological needs. SAHARA J.

[21] Marlow HM, Tolley EE, Kohli R, Mehendale S (2010). Sexual communication among married couples in the context of a microbicide clinical trial and acceptability study in Pune, India. Cult Health Sex.

[22] Abdool Karim Q, Abdool Karim SS, Frohlich JA, Grobler AC, Baxter C, Mansoor LE (2010). Effectiveness and safety of tenofovir gel, an antiretroviral microbicide, for the prevention of HIV infection in women. Science.

[23] Barker G, Ricardo C, Nascimento M (2007). Engaging men and boys in changing gender-based inequity in health: evidence from programme interventions.

[24] Shattuck D, Kerner B, Gilles K, Hartmann M, Ng'ombe T, Guest G (2011). Encouraging contraceptive uptake by motivating men to communicate about family planning: the Malawi Male Motivator project. Am J Public Health.

[25] Becker S (1996). Couples and reproductive health: a review of couple studies. Stud Fam Plan.

[26] World Health Organization (2012). Male involvement in the prevention of mother-to-child transmission of HIV.

[27] Aluisio A, Richardson BA, Bosire R, John-Stewart G, Mbori-Ngacha D, Farquhar C (2011). Male antenatal attendance and HIV testing are associated with decreased infant HIV infection and increased HIV-free survival. J Acquir Immune Defic Syndr.

[28] McCormack S, Ramjee G, Kamali A, Rees H, Crook AM, Gafos M (2010). PRO2000 vaginal gel for prevention of HIV-1 infection (Microbicides Development Programme 301): a phase 3, randomised, double-blind, parallel-group trial. Lancet.

[29] Montgomery CM, Pool R (2011). Critically engaging: integrating the social and the biomedical in international microbicides research. J Int AIDS Soc.

[30] Montgomery CM, Watts C, Pool R (2012). HIV and dyadic intervention: an interdependence and communal coping analysis. PLoS One.

[31] Pool R, Montgomery CM, Morar NS, Mweemba O, Ssali A, Gafos M (2010). A mixed methods and triangulation model for increasing the accuracy of adherence and sexual behaviour data: the Microbicides Development Programme. PLoS One.

[32] Skoler-Karpoff S, Ramjee G, Ahmed K, Altini L, Plagianos MG, Friedland B (2008). Efficacy of Carraguard for prevention of HIV infection in women in South Africa: a randomised, double-blind, placebo-controlled trial. Lancet.

[33] Abbott S, Friedland B, Katzen L Health concerns and HIV risk perception as motives to join a clinical trial.

[34] Abbott S, Friedland B, Katzen L, de Kock A, Madiba S, Cishe S Decision making and the influences of others in the informed consent process: a qualitative investigation in the Phase 3 study of Carraguard^®^.

[35] Abbott S, Friedland B, Katzen L, Madiba S, de Kock A, Cishe S What motivates women to enroll in microbicide clinical trials: results from the Phase 3 Carraguard^®^ Trial.

[36] Katzen L, Friedland B, Abbott S, de Kock A, Madiba S, Cishe S Qualitative evaluation of the informed consent process in the Phase 3 Carraguard^®^ Efficacy Trial: study staff experiences and recommendations for future trials.

[37] Madiba S, Friedland B, Abbott S, Katzen L, de Kock A, Cishe S Male partner involvement in the Carraguard^®^ Phase 3 Trial: data from a qualitative study of the informed consent process.

[38] Abbott S, Morar N, Madiba S, Katzen L, Phillip J, Mokgatle-Nthabu M Microbicides acceptability: the influence of social and cultural norms, interpersonal relations and sexual socialization.

[39] de Kock A, Abbott S, Morar N, Friedland B, Mtimkulu V, Cishe S Exploring microbicide acceptability in terms of socio-cultural norms, relationships and product attributes in the Carraguard^®^ Phase 3 Trial in South Africa.

[40] Marrazzo J, Ramjee G, Nair G, Palanee T, Mkhize B, Nakabiito C Pre-exposure prophylaxis for HIV in women: daily oral tenofovir, oral tenofovir/emtricitabine or vaginal tenofovir gel in the VOICE study (MTN 003).

[41] van der Straten A, Stadler J, Montgomery E, Hartmann M, Magazi B, Mathebula F (2014). Women's experiences with oral and vaginal pre-exposure prophylaxis: the VOICE-C qualitative study in Johannesburg, South Africa. PLoS One.

[42] Cohen CR, Brown J, Moscicki AB, Bukusi EA, Paull JR, Price CF (2011). A phase I randomized placebo controlled trial of the safety of 3% SPL7013 Gel (VivaGel(R)) in healthy young women administered twice daily for 14 days. PLoS One.

[43] Headley J, Baumgartner JN, Kaaya S, Minja A, Kalungula H, Bangapi D (2014). Adolescent and young women's acceptability and use of a proxy microbicide gel or pill in a mock trial in Tanzania.

[44] Tolley E, Kaaya S, Kaale A, Kalungula H, Headley J, Baumgartner JN Do adolescents under 18 years old warrant inclusion in HIV prevention clinical research? Comparing sexual risk patterns of adolescent and young adult women in a mock trial in Tanzania.

[45] Baumgartner JN, Kaaya S, Kaale A, Headley J, Tolley E Domestic violence among adolescents and young women in HIV prevention research in Tanzania: participant experiences and measurement issues.

[46] Tolley E, Kaaya S, Bangapi D, Minja A, Headley J, Kaale A Feasibility of recruiting adolescent women into a mock HIV prevention trial.

[47] Tolley E, Baumgartner JN, Kaaya S, Sastry J, Headley J Adolescent sexual risk and perceived need for PrEP: paving the way for their future participation in HIV prevention trials.

[48] Baumgartner JN, Tolley E, Kaaya S, Sastry J, Headley J Adolescent and community perspectives on microbicide trial participation in Tanzania and India.

[49] Sastry J, Tolley E Research on adolescent sex: to do or not to do.

[50] Venables E, Stadler J (2012). ‘The study has taught me to be supportive of her’: empowering women and involving men in microbicide research. Cult Health Sex.

[51] Padian NS, van der Straten A, Ramjee G, Chipato T, de Bruyn G, Blanchard K (2007). Diaphragm and lubricant gel for prevention of HIV acquisition in southern African women: a randomised controlled trial. Lancet.

# Sub article: An interdisciplinary framework for measuring and supporting adherence in HIV prevention trials of ARV-based vaginal rings

**DOI:** 10.7448/IAS.17.3.19158

**Published:** 2014-09-08

**Authors:** Kathleen M MacQueen, Elizabeth E Tolley, Derek H Owen, K Rivet Amico, Kathleen M Morrow, Thomas Moench, David R Friend, Barbara Friedland

**Affiliations:** 1 Social and Behavioral Health Sciences, FHI 360, Durham, NC, USA; 2 Contraceptive Technology Institute, FHI 360, Durham, NC, USA; 3 Department of Health Behavior and Health Education, School of Public Health, University of Michigan, Ann Arbor, MI, USA; 4 Centers for Behavioral & Preventive Medicine, The Miriam Hospital, Providence, RI, USA; 5 Department of Psychiatry & Human Behavior, Warren Alpert Medical School of Brown University, Providence, RI, USA; 6 Reprotect Inc, Baltimore, MD, USA; 7 The Johns Hopkins University, Baltimore, MD, USA; 8 Product Development, CONRAD, Arlington, VA, USA; 9 Population Council, New York, NY, USA

**Keywords:** vaginal ring, acceptability, adherence, clinical trial research, HIV prevention, measurement

## Abstract

**Introduction:**

Product adherence and its measurement have emerged as a critical challenge in the evaluation of new HIV prevention technologies. Long-acting ARV-based vaginal rings may simplify use instructions and require less user behaviour, thereby facilitating adherence. One ARV-based ring is in efficacy trials and others, including multipurpose rings, are in the pipeline. Participant motivations, counselling support and measurement challenges during ring trials must still be addressed. In previous HIV prevention trials, this has been done largely using descriptive and post-hoc methods that are highly variable and minimally evaluated. We outline an interdisciplinary framework for systematically investigating promising strategies to support product uptake and adherence, and to measure adherence in the context of randomized, blinded clinical trials.

**Discussion:**

The interdisciplinary framework highlights the dual use of adherence measurement (i.e. to provide feedback during trial implementation and to inform interpretation of trial findings) and underscores the complex pathways that connect measurement, adherence support and enacted adherence behaviour. Three inter-related approaches are highlighted: 1) adherence support – sequential efforts to define motivators of study product adherence and to develop, test, refine and evaluate adherence support messages; 2) self-reported psychometric measures – creation of valid and generalizable measures based in easily administered scales that capture vaginal ring use with improved predictive ability at screening, baseline and follow-up that better engage participants in reporting adherence; and 3) more objective measurement of adherence – real-time adherence monitoring and cumulative measurement to correlate adherence with overall product effectiveness through innovative designs, models and prototypes using electronic and biometric technologies to detect ring insertion and/or removal or expulsion. Coordinating research along these three pathways will result in a comprehensive approach to product adherence within clinical trials.

**Conclusions:**

Better measurement of adherence will not, by itself, ensure that future effectiveness trials will be able to address the most basic question: if the product is used per instructions, will it prevent HIV transmission? The challenges to adherence measurement must be addressed as one component of a more integrated system that has as its central focus adherence as a behaviour emerging from the social context of the user.

## Introduction

In July 2010, the CAPRISA 004 clinical trial of tenofovir 1% gel demonstrated that an ARV-based vaginal gel could prevent acquisition of HIV [1]. Subsequently, results from the iPrEX, Partners PrEP, TDF2 and Bangkok Tenofovir trials of oral ARV dosing further bolstered confidence in ARV-based prevention [2–5]. In each of these trials, sub-analyses indicated that poor adherence to the prescribed dosing regimen reduced efficacy. The importance of adherence to product use instructions was underscored by results from the VOICE and FEM-PrEP trials, where poor adherence to vaginal (VOICE) and oral (VOICE and FEM-PrEP) dosing regimens resulted in an inability to determine effectiveness [6, 7].

The delivery of microbicides through vaginal rings is perceived by some as a way to achieve better adherence, in part because rings have the potential to simplify the behavioural requirements for use. A number of long-acting ARV-based and multi-purpose prevention rings are in development. Two phase 3 clinical trials are now underway to assess the efficacy of a 28-day vaginal ring containing the antiretroviral drug dapivirine to reduce HIV transmission among African women [8]. Other rings in development include CONRAD's 90-day ring that contains both tenofovir and a contraceptive; the International Partnership for Microbicide's (IPM) dapivirine ring with or without a contraceptive added that would be effective for 30 days and potentially up to 60–90 days; the Population Council's 90-day ring containing MIV-150, zinc acetate and carrageenan, with or without a contraceptive, designed to protect against HIV, herpes simplex virus type-2 and human papillomavirus (Barbara Friedland, personal communication, 2014); and Merck's project to develop their ethylene vinyl acetate copolymer contraceptive ring as a method for also delivering ARVs. It is anticipated that over the next three years, multiple products will move from preclinical to clinical testing of safety and pharmacokinetics during use ranging from 30 days to 12 months.

Social science research with participants in trials of microbicides and PrEP indicate that a wide array of factors may potentially influence adherence. For example, lack of understanding about clinical research practices (e.g. how biological specimens will be used) [9], concerns about product safety [10] and distrust of the motivations of researchers and trial sponsors (e.g. motivated by negative values such as greed and racism) [9, 11, 12] are frequently described for HIV prevention research participants and communities. For women, decisions about whether and what to disclose to male partners about product use and research participation reflect culturally regulated gender dynamics that can either generate support or create obstacles for a woman's decision to participate in a trial and adhere to product use [10, 13–17]. There is also increasing recognition of the fact that the on-going and intensive level of interaction among participants in the research clinic waiting room and between participants and research staff foster the emergence of a research culture that may exacerbate or mitigate these other challenges [10].

Research conducted by IPM in preparation for phase 3 trials of the 30-day dapivirine ring found that women reported removal or expulsion of the ring between study visits at frequencies ranging from 4 to 18% of participants over varying time frames [18, 19]. The most common reasons cited for ring removal were menses, periodic cleaning and expulsion during urination or defecation. Currently, self-report is the primary method for estimating the number of hours or days that a removed or expelled ring remains outside the vagina before it (or a new ring) is inserted. Although self-reported adherence has been shown to over-estimate product use to varying degrees in ARV-based HIV prevention trials, collecting information on reasons for non-adherence and participants’ experiences with product use more generally is essential to providing effective counselling and support for proper use of all such products, including vaginal rings. Recognition of adherence challenges is necessary but not sufficient to ensure that participants receive adequate support for adherence at the level needed to evaluate product effectiveness. Also important are motivations for adherence within the context of a clinical trial. Most research on adherence outcomes has focused on the treatment context, where the goal is to maximize health benefits for clients. In the trial context, the health benefits of product use are unclear and may be non-existent, especially if there is randomization to a placebo arm. There is also greater ambiguity about the potential risks than is normally the case with approved medications. These unique clinical trial elements must be considered in optimizing clinical trial adherence to ring use. Additional consideration must be given to how the clinical trial elements are situated relative to the social context of the trial participants, for example, how the motivations of researchers are understood in the community.

Because vaginal rings can be inserted and removed by the user, direct observation of enacted adherence, that is, the actual act of using the product over time, is not feasible. In this regard, the adherence measurement challenges for rings are similar to those faced in identifying a valid, reliable and accurate measure of microbicide adherence with respect to other drug delivery systems, which has been elusive [20]. Proxy measures of adherence for microbicides specifically, and for PrEP more generally, have ranged from self-reported counts of use/non-use to measurement of drug concentration in the blood and vaginal fluids. All proxy measures have strengths and weaknesses [21]. For example, in the case of vaginal rings, the wide intra-individual variability in drug blood levels during use creates challenges for accurately assessing how long an ARV-based ring has been inserted or if it has been removed or expelled and reinserted [22]. Efforts are underway to identify biomarkers of adherence, for example, measurement of residual drug in rings, the quantification of biofilm on the surface of vaginal rings to assess duration of insertion and assessment of plasma drug levels aggregated by site. Nonetheless, adherence measurement remains dependent, at least in part, on self-report, which is known to be affected by social desirability biases and cognitive limits on recall [23, 24].

No single approach to measuring ring adherence will be adequate to provide the level of detail needed to 1) monitor adherence in real time to target participants for focused discussions around product use, 2) allow for statistical inferences about effectiveness while controlling for adherence and 3) inform planning for adherence assessments during programmatic implementation of effective products. Increasingly, researchers are advocating for use of several adherence measures that can be triangulated [25, 26].

While considerable innovation is emerging to provide adherence support and develop more objective measures, approaches have been largely specific to a particular study or study-site. Furthermore, the adherence support procedures used to date in microbicide or PrEP trials have been derived from theory and practice established mainly in the treatment context, with no evaluative piloting for the experimental prevention research context. In the CAPRISA 004, iPrEX and VOICE trials adherence support procedures underwent modification after trials were well underway [27, 28]. Empirical data on the fidelity of research teams to adherence support guidelines is limited, further undermining efforts to understand the effectiveness of a given adherence support approach. Biometric measures such as drug levels have similarly undergone continual refinement in the context of the very trials where those measures have been used to evaluate adherence. Innovative approaches sometimes have difficulty getting traction in clinical trials, due to concerns about their impact on trial implementation in the absence of pilot data.

## Discussion

### Developing an interdisciplinary framework

An interdisciplinary framework is needed to move past the current ad hoc approach and integrate the various dimensions of adherence assessments without burdening or detracting from on-going product development or trial implementation. Figure 1 describes the key components of such a framework for understanding, measuring and supporting ring adherence. This framework highlights the dual use of adherence measurement (i.e. to provide feedback during trial implementation and to inform interpretation of trial findings) and underscores the complex pathways that connect measurement, adherence support and enacted adherence behaviour.

**Figure 1:** An integrated framework for adherence support and measurement in clinical trials of ARV-based vaginal rings for HIV prevention.

### Adherence support process

Secondary analysis of data from the CAPRISA 004 trial suggests that use of a theory-driven approach to support adherence to product use instructions can be effective. Midway through the CAPRISA 004 trial the investigators revised their adherence support approach to a counselling package based on motivational interviewing, referred to as Adherence Support Program (ASP). Findings indicate that implementation of ASP was associated with an increase in the proportion of women reporting high adherence, an increase in detectable tenofovir levels and an increase in the estimate of product effectiveness [29]. Similarly, the Uganda Partners PrEP site conducted an ancillary adherence counselling intervention study with a subset of participants. The intervention was adapted from an evidence-based HIV treatment adherence intervention called Lifesteps [30] that included cognitive-behavioural, motivational interviewing, and problem-solving techniques and significantly increased adherence as measured by unannounced pill counts [31].

In the iPrEX PrEP trial among MSM, which also demonstrated product effectiveness, a different but somewhat related approach was developed called next step counselling (NSC). NSC was designed to promote open discussions about product use and to build motivation and positive regard for the use of an unproven product (or potentially a placebo product) within a clinical trial [32]. In conjunction with NSC, a neutral assessment (NA) approach was also implemented where rates of product use were collected by non-counselling team members who were trained to intentionally remove judgment and negative consequences (e.g. extensive follow-up, probing or additional procedures) for reporting product non-use. The combined NSC/NA approach subsequently formed the basis for adherence support procedures called VASP (VOICE adherence strengthening program) used in the latter half of the VOICE trial. Neither trial had a comparison arm by which to evaluate the combined approach. However, unlike the CAPRISA 004 and Uganda Partners PrEP findings, mid-trial adoption of VASP did not appear to increase adherence when individual adherence was examined pre- and post-VASP implementation. A NSC/NA type of approach was used in the early part of FACTS 001 (an on-going follow-on phase 3 trial to replicate CAPRISA 004), which was subsequently switched to using the ASP approach. The IPM and MTN trials currently assessing IPM's 28-day dapivirine vaginal ring draw on a variety of adherence support strategies. The MTN study uses a VASP-style approach, called Adherence Counseling Education (ACE) and a number of small group-focused discussions and activities. Common elements across all of the ARV-based trials described above include aspects of motivational interviewing and client centred models originally developed outside the research context; some also include use of product returns, self-report or biomarker (drug level) measures of product non-use to target adherence counselling with participants.

While current efforts to address the adherence support challenges in real-time with on-going clinical trials are theory-based and draw on behavioural science methods, none of the approaches used in blinded biomedical randomized controlled trials of HIV prevention have been evaluated rigorously for that context. The motivational interviewing and client-centred counselling approaches used in these trials were derived from models largely developed with U.S. populations and focused on counselling intended to engage clients in the dynamics of change, that is, moving from a behaviour or set of behaviours that are recognized as costly/unhealthy to ones that are beneficial/healthy [33]. Counsellors sought to promote investment in change, avoiding overt instruction or persuasion about what clients “should” do. Instead, counsellors respected clients’ reasons *not* to change, provided a safe place for full expression of this resistance to change, and facilitated genuine development of an appreciation and desire for an alternative to current behaviour that maximized values and perceived benefits from a client's perspective.

The reality of clinical trial research limits implementation of full motivational interviewing principles. For example, while participants in randomized controlled trials have autonomy with regard to deciding whether to enrol or whether to comply with all study requirements, they lack autonomy to actively choose the intervention to be administered – a component of choice that is core to the MI approach. Similarly, given the critical importance of protocol compliance for successful trial implementation, it is unlikely that research staff can maintain genuine appreciation for a participant's right not to adopt the trial-desired behaviour. Finally, the primary motivational basis behind MI is undermined by the potential lack of a direct (or any) health benefit accruing to the participant as a result of adopting the clinically-desired adherence behaviour.

In contrast to motivational interviewing-based approaches, compliance-oriented approaches rely on overt persuasion, use of external reinforcements or the threat of negative consequences to encourage clients to adopt a behaviour judged by an external expert to be “better for you.” However, the use of pure persuasion can be alienating and could create negative feelings towards product use and trial participation, which could further deteriorate motivation to report on product non-use in the clinical research setting. The use of motivational interviewing-based engagement with participants, with its valuing of genuine discourse and attempt to balance power between researchers and participants is arguably a better fit with the participatory kinds of engagement reflective of current standards for HIV prevention research practice [34].

Effective support for study product use in blinded HIV prevention trials requires a theory-based, informed approach that meets the specific needs of both trial participants and the trial itself. Such a *participant-centred* approach would leverage respect for autonomy, as classically defined for human research protections [35, 36], while opening a dialog to identify, develop and foster personal investment in the objectives of the prevention trial. It would acknowledge, and then work with the uncertainty of a direct health benefit and the unknown health risks accruing to the participant as a result of adherence in the experimental context of a prevention trial. It would also explicitly recognize the cultural and social dynamics that enter into a person's motivations to enrol in a trial, continue active engagement, take up product use as assigned and adhere to study requirements as requested. Unique to a participant-centred approach, the intervention focus would be the participants’ motivation towards and skills in achieving full engagement with the research goals of the study rather than specifically on product use, per se. Persistent, as-recommended product use would be one of several desired outcomes of personal engagement in the study.

### Adherence support fidelity

Anecdotal evidence suggests that fidelity to adherence support protocols by trial staff may be a significant but unmeasured factor in participant adherence to instructions for product use. The importance of fidelity was underscored in a recent assessment of MI to support ARV treatment adherence in South Africa [37]. Yet fidelity measures are rarely included in HIV prevention trials. Considerable attention has been placed on the failure of women to follow product use instructions in the FEM-PrEP and VOICE trials but very little has been focused on whether those providing the instructions and adherence support were doing so in compliance with the standards and procedures developed for the trials. In the absence of fidelity measures it is impossible to determine the relative contributions of participant motivation, adherence support system design and provider implementation. An effective adherence support process will not ensure adequate levels of adherence if it is poorly implemented.

### Adherence psychometrics

Despite shortcomings, the assessment of self-reported adherence remains a standard component of trials because it is inexpensive, relatively non-invasive and allows for immediate feedback [21]. Methods to improve validity and reliability of self-reported adherence measures have largely focused on mode of data collection (e.g. use of ACASI). However, there has been a lack of support for development of psychometric measures similar to those long used to assess treatment non-compliance [38, 39]. Psychometrically validated scales are composed of multiple items in the form of questions or statements that, when combined, measure a more complex concept that may not be directly observable [40–42]. As such, they may better assess the multiple factors likely to contribute to non-adherence.

Drawing on behavioural theory and existing social and behavioural research on acceptability and adherence, it should be feasible to identify a multidimensional set of items that assess adherence to products in the HIV prevention pipeline. The process for developing such psychometric measures is fairly straightforward. The first step is to identify relevant conceptual domains that influence ring adherence, developing specific items that adequately represent each domain. Both the published literature and unpublished qualitative data may be consulted to determine potential domains, such as product-related attitudes (i.e. perceived benefits and barriers to ring use), motivations for product use within a clinical trial setting and perceived self-efficacy to use a ring as instructed, as well as barriers potentially associated with non-adherence (i.e. low levels of knowledge, social stigma associated with product use, male partner concerns, domestic violence, lack of mobility.) Content validity – the degree to which the domains and draft items represent the underlying dimensions of vaginal ring adherence behaviour within a clinical trial context – would be assessed in part through review and feedback by a panel of behavioural and social researchers, cultural experts and/or clinicians with expertise in HIV prevention adherence. Both the content, sequence and wording of draft items, as well as the overall framing and instructions for scale administration, must then be evaluated in a small number of culturally diverse sites, using standard cognitive interviewing techniques and/or focus group discussions, to verify that the draft measure includes relevant content and that individual items are salient, well understood and easily rated by participants similar to those who might take part in vaginal ring trials.

After the draft scale items are refined, they are incorporated into a survey that also includes measures of behaviours or attitudes that are hypothesized to predict or affect adherence directly or indirectly. The survey is administered to women in one or more cultural settings who are current or former trial product users. Exploratory and confirmatory factor analysis techniques are applied to determine the final structure and content of the adherence scale, and the theoretical associations between adherence as measured by the scale and other factors/variables are explored. Once the psychometric properties of the scale measure is confirmed, opportunities to assess the predictive value of the adherence scale can then be sought, for example, by examining how well the scale score correlates with biometric indicators of product use.

A validated scale of vaginal ring adherence would potentially have multiple applications within the context of HIV prevention clinical research. It could support the trial's evaluation of vaginal ring efficacy by providing complementary evidence on adherence, particularly when biomarker adherence data may be missing or incomplete for some trial participants. The use of a composite biomarker-plus-adherence scale approach was shown to correlate better with viral load than use of biomarker data alone in one study on HAART effectiveness [43]. Additionally, given the multidimensional composition of a vaginal ring adherence scale, it could alert staff to a participant's potential issues around adherence at baseline (if, for example, commitment to correct and continuous product use ranked low among an individual's motivations for trial participation) and/or provide real-time feedback on barriers or facilitators of use to adherence support staff over the course of the trial. While the process of developing adherence psychometrics requires an upfront investment of time and resources, once a scale is validated it is a cost-effective tool to adapt and implement across the applications described above.

### Adherence biometrics

There has been considerable attention given to the development of biomarkers and biometric measures that rely less on verbal self-report by participants and provide more direct measures of events or adherence. Examples include electronic devices such as MemsCap and WisePill devices for pill storage and Wisebag for microbicide applicator storage, which allow electronic tracking of when the storage devices are opened, ostensibly for use (or, in the case of rings, for storage) of the product [44, 45]. Some biometric approaches may indicate a point-in-time when the product is used, while other approaches may indicate more cumulative use patterns. For example, two approaches currently under investigation with the potential to measure varying durations of ring use include quantification of ring biofilm (which should increase with duration of insertion) and of residual drug (which should decrease with duration of insertion). Other biometric methods have the potential to indicate ring insertion at a given point in time. One such approach is the adaptation of an esther taggant previously tested in vaginal gel to ring technologies [46]. Another approach with some potential is the use of an RFID tag that would allow random non-intrusive scanning for ring insertion. Rather than measuring ring insertion, some biometric approaches might indicate current or cumulative ring removal. For example, it might be possible to develop a cumulative measure of ring exposure to air that could indicate the proportion of time the ring was removed, or exposure to water that might indicate removal and washing.

### Adherence analytics

To address vaginal ring adherence in the comprehensive way described above, we need to consider the analytical integration of the various approaches. As illustrated in Figure 1, analytic findings from each framework component may inform other components as well as ensuring that the best science is used within each component.

Individual elements of comprehensive adherence support processes should minimally be pretested through use of cognitive interviewing techniques and piloted using comprehension assessments on the part of participants and fidelity assessments with regard to implementation by research staff prior to their use in effectiveness trials. Experimental or quasi-experimental assessments of the comparative effectiveness of different adherence support system designs would be ideal. Formative research to develop adherence support can also inform development of the psychometric measures and determine acceptability of various biometric measurement approaches in specific cultural contexts.

Examples of fidelity measures that should be included in ARV-based HIV prevention trials include the quality and extent of staff training in the method, quality and consistency in implementation, organizational support for implementation, documentation of practices and adaptation to local and emergent needs. Use of mobile phones or other technology may also be helpful in monitoring fidelity as well as adherence on the part of participants. Here again, formative research findings will be critical for developing and adapting measures to specific trials and contexts.

Points of potential interface between psychometric and biometric measurement and adherence support should be identified and consciously and overtly considered when designing measures, support strategies and guidelines for administering each.

## Conclusions

After more than two decades of biomedical HIV prevention effectiveness trials, it is now clear that randomization, blinding and use of placebo controls cannot mitigate any behavioural or sociocultural context-specific issues that might arise during the course of a trial. Rather, behaviours and contexts that drive adherence are at issue for evaluating effectiveness. Increasingly, researchers are advocating for use of several adherence measures that can be triangulated [19, 38]. Coordinated research is needed along three pathways to generate complementary measures that can then be triangulated for a comprehensive picture: 1) psychometrics to improve self-report measures reflective of behaviour; 2) electronic dispensing or storage options to capture events corresponding to intended product use or non-use; and 3) biometrics to capture indications of product ingestion or insertion, as well as frequency or duration of same. Better measurement of adherence will not, by itself, ensure that future effectiveness trials will be able to address the most basic question: does the product prevent HIV transmission? The challenges to adherence measurement must be addressed as one component of a more integrated system that has as its central focus adherence as a context-driven behaviour.

## References

[1] Abdool Karim Q, Abdool Karim SS, Frohlich JA, Grobler AC, Baxter C, Mansoor LE (2010). Effectiveness and safety of tenofovir gel, an antiretroviral microbicide, for the prevention of HIV infection in women. Science.

[2] Grant RM, Lama JR, Anderson PL, McMahan V, Liu AY, Vargas L (2010). Preexposure chemoprophylaxis for HIV prevention in men who have sex with men. N Engl J Med.

[3] Thigpen MC, Kebaabetswe PM, Paxton LA, Smith DK, Rose CE, Segolodi TM (2012). Antiretroviral preexposure prophylaxis for heterosexual HIV transmission in Botswana. N Engl J Med.

[4] Choopanya K, Martin M, Suntharasamai P, Sangkum U, Mock PA, Leethochawalit M (2013). Antiretroviral prophylaxis for HIV infection in injecting drug users in Bangkok, Thailand (the Bangkok Tenofovir Study): a randomised, double-blind, placebo-controlled phase 3 trial. Lancet.

[5] Baeten JM, Donnell D, Ndase P, Mugo NR, Campbell JD, Wangisi J (2012). Antiretroviral prophylaxis for HIV prevention in heterosexual men and women. N Engl J Med.

[6] van der Straten A, Van Damme L, Haberer JE, Bangsberg DR (2012). Unraveling the divergent results of pre-exposure prophylaxis trials for HIV prevention. AIDS.

[7] Van Damme L, Corneli A, Ahmed K, Agot K, Lombaard J, Kapiga S (2012). Preexposure prophylaxis for HIV infection among African women. N Engl J Med.

[8] International Partnership for Microbicides Two phase III sister studies of a microbicide ring to prevent HIV: the Ring Study & ASPIRE. International Partnership for Microbicides.

[9] Macqueen KM, Alleman P (2008). International perspectives on the collection, storage, and testing of human biospecimens in HIV research. Ethics Hum Res.

[10] van der Straten A, Stadler J, Montgomery E, Hartmann M, Magazi B, Mathebula F (2014). Women's experiences with oral and vaginal pre-exposure prophylaxis: the VOICE-C qualitative study in Johannesburg, South Africa. PLoS One.

[11] Mack N, Robinson ET, MacQueen KM, Moffett J, Johnson LM (2010). The exploitation of “Exploitation” in the tenofovir prep trial in Cameroon: lessons learned from media coverage of an HIV prevention trial. J Empir Res Hum Res Ethics.

[12] Saethre E, Stadler J (2013). Malicious whites, greedy women, and virtuous volunteers: negotiating social relations through clinical trial narratives in South Africa. Med Anthropol Q.

[13] Woodsong C, Macqueen K, Namey E, Sahay S, Morar N, Mlingo M (2006). Women's autonomy and informed consent in microbicides clinical trials. J Empir Res Hum Res Ethics.

[14] Mngadi KT, Maarschalk S, Grobler AC, Mansoor LE, Frohlich JA, Madlala B (2014). Disclosure of microbicide gel use to sexual partners: influence on adherence in the CAPRISA 004 trial. AIDS Behav.

[15] Succop SM, MacQueen KM, van Loggerenberg F, Majola N, Abdool Karim Q, Abdool Karim SS (2014). Trial participation disclosure and gel use behavior in the CAPRISA 004 tenofovir gel trial. AIDS Care.

[16] Montgomery ET, van der Straten A, Chidanyika A, Chipato T, Jaffar S, Padian N (2011). The importance of male partner involvement for women's acceptability and adherence to female-initiated HIV prevention methods in Zimbabwe. AIDS Behav.

[17] Ware NC, Wyatt MA, Haberer JE, Baeten JM, Kintu A, Psaros C (2012). What's love got to do with it? Explaining adherence to oral antiretroviral pre-exposure prophylaxis for HIV-serodiscordant couples. J Acquir Immune Defic Syndr.

[18] Montgomery ET, van der Straten A, Cheng H, Wegner L, Masenga G, von Mollendorf C (2012). Vaginal ring adherence in sub-Saharan Africa: expulsion, removal, and perfect use. AIDS Behav.

[19] Nel A, Kamupira M, Woodsong C, van der Straten A, Montgomery E, van Niekerk N (2012). Safety, acceptability and pharmacokinetic assessment (adherence) of monthly dapivirine vaginal rings (Ring-004) for HIV prevention.

[20] van der Straten A, Montgomery ET, Hartmann M, Minnis A (2013). Methodological lessons from clinical trials and the future of microbicide research. Curr HIV/AIDS Rep.

[21] Tolley EE, Harrison PF, Goetghebeur E, Morrow K, Pool R, Taylor D (2010). Adherence and its measurement in phase 2/3 microbicide trials. AIDS Behav.

[22] Kiser PF, Johnson TJ, Clark JT (2012). State of the art in intravaginal ring technology for topical prophylaxis of HIV infection. AIDS Rev.

[23] Fenton KA, Johnson AM, McManus S, Erens B (2001). Measuring sexual behaviour: methodological challenges in survey research. Sex Transm Infect.

[24] Catania JA, Binson D, Van Der Straten A, Stone V (1995). Methodological research on sexual behavior in the AIDS era. Ann Rev Sex Res.

[25] Muchomba FM, Gearing RE, Simoni JM, El-Bassel N (2012). State of science of adherence in pre-exposure prophylaxis and microbicide trials. J Acquir Immune Defic Syndr.

[26] Pool R, Montgomery CM, Morar NS, Mweemba O, Ssali A, Gafos M (2010). A mixed methods and triangulation model for increasing the accuracy of adherence and sexual behaviour data: the Microbicides Development Programme. PLoS One.

[27] Amico KR, Mansoor LE, Corneli A, Torjesen K, van der Straten A (2013). Adherence support approaches in biomedical HIV prevention trials: experiences, insights and future directions from four multisite prevention trials. AIDS Behav.

[28] Amico KR, McMahan V, Goicochea P, Vargas L, Marcus JL, Grant RM (2012). Supporting study product use and accuracy in self-report in the iPrEx study: next step counseling and neutral assessment. AIDS Behav.

[29] Mansoor LE, Karim QA, Werner L, Madlala B, Ngcobo N, Cornman DH (2014). Impact of an adherence intervention on the effectiveness of tenofovir gel in the CAPRISA 004 trial. AIDS Behav.

[30] Safren SA, Otto MW, Worth JL, Salomon E, Johnson W, Mayer K (2001). Two strategies to increase adherence to HIV antiretroviral medication: life-steps and medication monitoring. Behav Res Ther.

[31] Psaros C, Haberer JE, Katabira E, Ronald A, Tumwesigye E, Campbell JD (2014). An intervention to support HIV pre-exposure prophylaxis (PrEP) adherence in HIV serodiscordant couples in Uganda. J Acquir Immune Defic Syndr.

[32] Holstad MM, Foster V, Diiorio C, McCarty F, Teplinskiy I (2010). An examination of the psychometric properties of the Antiretroviral General Adherence Scale (AGAS) in two samples of HIV-infected individuals. J Assoc Nurses AIDS Care.

[33] Rubak S, Sandbaek A, Lauritzen T, Christensen B (2005). Motivational interviewing: a systematic review and meta-analysis. Br J Gen Pract.

[34] Joint United Nations Programme on HIV/AIDS (UNAIDS), AVAC (2011). Good participatory practice guidelines for biomedical HIV prevention trials.

[35] Participants in the Conference on Ethical Aspects of Research in Developing Countries (2002). Ethics. Fair benefits for research in developing countries. Science.

[36] Rennie S, Sugarman J (2010). Developing ethics guidance for HIV prevention research: the HIV Prevention Trials Network approach. J Med Ethics.

[37] Dewing S, Mathews C, Schaay N, Cloete A, Louw J, Simbayi L (2012). Behaviour change counselling for ARV adherence support within primary health care facilities in the Western Cape, South Africa. AIDS Behav.

[38] Morisky DE, Green LW, Levine DM (1986). Concurrent and predictive validity of a self-reported measure of medication adherence. Med Care.

[39] Johnson MO, Neilands TB, Dilworth SE, Morin SF, Remien RH, Chesney MA (2007). The role of self-efficacy in HIV treatment adherence: validation of the HIV Treatment Adherence Self-Efficacy Scale (HIV-ASES). J Behav Med.

[40] DeVellis RF (2003). Scale development theory and applications.

[41] Mahoney CA, Thombs DL, Howe CZ (1995). The art and science of scale development in health education research. Health Educ Res.

[42] Gamst G, Meyers L, Guarino AJ, Burke HM, Guest G, Namey E (2015). Scale development and validation. Public health research methods [Internet].

[43] Liu H, Golin CE, Miller LG, Hays RD, Beck CK, Sanandaji S (2001). A comparison study of multiple measures of adherence to HIV protease inhibitors. Ann Intern Med.

[44] Haberer JE, Baeten JM, Campbell J, Wangisi J, Katabira E, Ronald A (2013). Adherence to antiretroviral prophylaxis for HIV prevention: a substudy cohort within a clinical trial of serodiscordant couples in East Africa. PLoS Med.

[45] Gengiah TN, Upfold M, Naidoo A, Mansoor LE, Feldblum PJ, Abdool Karim Q (2014). Monitoring microbicide gel use with real-time notification of the container's opening events: results of the CAPRISA Wisebag study. AIDS Behav.

[46] Morey TE, Wasdo S, Wishin J, Quinn B, van der Straten A, Booth M (2012). Feasibility of a breath test for monitoring adherence to vaginal administration of antiretroviral microbicide gels. J Clin Pharmacol.

# Sub article: Comparing patterns of sexual risk among adolescent and young women in a mixed-method study in Tanzania: implications for adolescent participation in HIV prevention trials

**DOI:** 10.7448/IAS.17.3.19149

**Published:** 2014-09-08

**Authors:** Elizabeth E Tolley, Sylvia Kaaya, Anna Kaale, Anna Minja, Doreen Bangapi, Happy Kalungura, Jennifer Headley, Joy Noel Baumgartner

**Affiliations:** 1 Social and Behavioral Health Sciences, FHI 360 Durham, NC, USA; 2 Muhimbili University of Health and Allied Services, Dar es Salaam, Tanzania

**Keywords:** adolescents, clinical trials research, sexual and reproductive health, HIV prevention, Tanzania

## Abstract

**Introduction:**

Despite the disproportionate impact of HIV on women, and adolescents in particular, those below age 18 years are underrepresented in HIV prevention trials due to ethical, safety and logistical concerns. This study examined and compared the sexual risk contexts of adolescent women aged 15–17 to young adult women aged 18–21 to determine whether adolescents exhibited similar risk profiles and the implications for their inclusion in future trials.

**Methods:**

We conducted a two-phase, mixed-method study to assess the opportunities and challenges of recruiting and retaining adolescents (aged 15–17) versus young women (18–21) in Tanzania. Phase I, community formative research (CFR), used serial in-depth interviews with 11 adolescent and 12 young adult women from a range of sexual risk contexts in preparation for a mock clinical trial (MCT). For Phase II, 135 HIV-negative, non-pregnant adolescents and young women were enrolled into a six-month MCT to assess and compare differences in sexual and reproductive health (SRH) outcomes, including risky sexual behaviour, incident pregnancy, sexually transmitted infections (STIs), reproductive tract infections (RTIs) and HIV.

**Results:**

In both research phases, adolescents appeared to be at similar, if not higher, risk than their young adult counterparts. Adolescents reported earlier sexual debut, and similar numbers of lifetime partners, pregnancy and STI/RTI rates, yet had lower perceived risk. Married women in the CFR appeared at particular risk but were less represented in the MCT. In addition, adolescents were less likely than their older counterparts to have accessed HIV testing, obtained gynaecological exams or used protective technologies.

**Conclusions:**

Adolescent women under 18 are at risk of multiple negative SRH outcomes and they underuse preventive services. Their access to new technologies such as vaginal microbicides or pre-exposure prophylaxis (PrEP) may similarly be compromised unless greater effort is made to include them in clinical trial research.

## Introduction

In 2010, 34 million people were living with HIV and approximately half of the adult cases were among women [1]. Although estimates of HIV incidence have begun to decline in general populations, young African women aged 15–24 continue to bear a high burden of the disease [1, 2]. Indeed, in Tanzania, HIV prevalence in young women is approximately 3%, three-fold higher than in men of the same age group [3]. As elsewhere, Tanzanian young women's risk of HIV has been attributed to early sexual debut [4] and fuelled by poverty and socio-cultural conditions that undermine the use of currently available HIV prevention methods, such as condoms [5–10] .

Despite the disproportionate impact of HIV on young women, they are markedly underrepresented in clinical trials of new HIV prevention methods. Although a number of potential prevention approaches, including antiretroviral-based vaginal and oral products, have been evaluated in women aged 18 and older, only two trials to date have enrolled women below the age of 18 [11–13] .

There is some recent evidence that younger adults in PrEP trials have poorer product adherence and trial retention, thus undermining our ability to show product effectiveness [14]; however, many have argued that adolescents’ inclusion in clinical microbicide trials is a matter of social justice, providing important opportunities to assess the safety, acceptability and effectiveness of new prevention products within a high-risk population who might differ from their young adult counterparts in important ways [11, 15–17]. The social justice argument may well supersede the logistical needs of trials, or rather, further highlight the special attention and work required to adequately include adolescents in trials. Furthermore, because regulatory bodies such as the United States Food and Drug Administration and the South African Medicines Control Council are unlikely to allow microbicides to be marketed to adolescents without data demonstrating that these products are safe and effective for the adolescent population [18], their exclusion is likely to delay or even limit adolescent access to new HIV prevention products.

Nevertheless, multiple barriers exist to the recruitment of adolescent women, including ethical concerns about younger women's biological safety and their cognitive ability to give informed consent for trial participation, as well as the legal and social challenges of recruiting young, sexually active minors [15, 16, 18]. In addition, although epidemiological research clearly identifies young women as a high-risk group for HIV, little research has been conducted to distinguish the risk behaviours and cultural contexts that put specific adolescents at risk [19]; without more information on adolescent sexual behaviour, it is uncertain whether younger women's patterns of sexual risk behaviours would even make them good candidates for trial participation.

In order to assess the opportunities and challenges of recruiting adolescent women into HIV prevention clinical trials, we conducted a mixed-method study in Dar es Salaam, Tanzania that included community formative research (CFR) and a six-month mock clinical trial (MCT). The specific objectives of the CFR were manifold but included gathering more in-depth information from socio-demographically diverse adolescents and young adult women representative of the types of participants we anticipated being recruited into the MCT in order to finalize study procedures and measures for the MCT. For this paper, we use data from both the CFR and the MCT to identify and compare the sexual relationship patterns, HIV risk perception, HIV risk reduction behaviours and incident sexual and reproductive health (SRH) outcomes of adolescents aged 15–17 with those of young women aged 18–21 to determine whether they face similar or unique risks and to determine how such risks might affect trial participation.

## Methods

This study comprised two research phases – the CFR and the MCT. During the CFR phase, we conducted a total of 64 in-depth interviews (IDI) – for up to three IDIs each with 23 sexually active young women aged 15–21 from different sexual risk contexts: married adolescents, married young women, single in-school and single out-of-school adolescents and young women, of whom some were currently or previously engaged in sex work. [Note: two participants completed only the first IDI and one participant completed the first and second interview, but not the third.] The study team worked with representatives from community-based organizations and schools, community and government leaders and members of a “Youth Interactive Group” (YIG) to identify and recruit participants from 14 very-low-income residential locations with high concentrations of bars, brothels and retail alcohol outlets. Interviews were conducted in Swahili by trained female interviewers, audio-recorded, transcribed and uploaded into NVivo8, a software programme that assists with qualitative data management and analysis. The first and second interviews explored participants’ home life, school or work situation, first and current sexual relationships, reproductive and sexual health knowledge and use of protective behaviours. The third interview focused on their attitudes towards pre-exposure prophylaxis (PrEP), microbicides and participation in HIV prevention clinical research.

During the second research phase, we enrolled 135 sexually active, HIV-negative, non-pregnant adolescents and young women aged 15–21 into a MCT with interviews at baseline, two, four and six months. Research staff conducted informational meetings in communities identified during the CFR and worked with community representatives and YIG members to recruit participants for the MCT. The study was conducted at the Infectious Disease Clinic (IDC) in Dar es Salaam, which offers youth-friendly services. During the study, participants received urine pregnancy tests at each visit and were tested for HIV, bacterial vaginosis (BV) (via gram stain test) and trichomoniasis (via microscopy), at baseline and month four. Those who tested positive were called and offered treatment. They also received behavioural assessments at every visit, with more extensive assessments at baseline and six months.

Qualitative CFR data were analysed thematically following a process of reading, coding and the development of analytic memos and data reduction matrices [20]. Initial codes were proposed and discussed by the US and the Tanzanian research team after reading the first transcripts from interview 1. The codebook was updated after each round and coding took place in parallel to data collection. Memos identified and described dimensions of coded themes (i.e. identifying common contexts within the *HIV Risk Perception* code in which participants did or did not express concern about HIV risk). Important themes/sub-themes were quantified and typed into Excel matrices to identify patterns across age groups and/or risk contexts. For the MCT, we conducted chi-square tests or Fisher's exact tests, as appropriate depending on cell size, to examine bivariate associations of socio-demographic, sexual risk behaviour and SRH risk outcomes. Given our small sample size, we did not further develop multivariable models to assess differences in adolescent versus young women's risk profiles.

Ethical approvals were received from Muhimbili University of Health and Allied Services, the National Institute for Medical Research and FHI 360's Protection of Human Subjects Committee. Participants aged 16 or older gave written informed consent; for 15-year-old participants, a parent/guardian and the participant gave written consent.

## Results

### CFR phase

A total of 23 adolescents and young women participated in the CFR phase. Our analysis of sexual relationships, risk reduction behaviours and risk perception revealed few differences by age group, but many more by risk context (Table 1). More than two-thirds of CFR participants had fractured family lives in which one or both parents had died or were divorced. In many cases – but especially among five young women engaged in sex work – these precarious beginnings appeared to enhance their vulnerability. Each of these young women described a scenario in which the death or divorce of parents and the inability or unwillingness of other relatives to help care for their needs led to their entry into sex work. All but one lived on their own, in a brothel or with a friend. This is in contrast to in-school youth, all of whom were living with one or both parents.

**Table 1:** Community formative phase characteristics by risk group and age

	Sex workers (*N*=5)	Married participants (*N*=8)	Single, out-of-school (*N*=5)	Single, in school (*N*=5)	Adolescents (15–17) (*N*=11)	Young women (18–21) (*N*=12)

	*N* (%)	*N* (%)	*N* (%)	*N* (%)	*N* (%)	*N* (%)
Mean age	17.2	18.4	18.0	18.4	16.4	19.6
Mean years of education	8.4	6.9	8.3	10.2	7.2	9.3
Living with one or both parents	1 (20)	1 (13)	1 (20)	5 (100)	4 (36)	4 (33)
Living with husband or partner	0 (0)	7 (87)	0 (0)	0 (0)	3 (27)	4 (33)
Living with relative	0 (0)	0 (0)	3 (60)	0 (0)	3 (27)	0 (0)
Living on own (brothel/with friend)	4 (80)	0 (0)	1 (20)	0 (0)	1 (9)	4 (33)
Parents separated/divorced	2 (40)	1 (13)	3 (60)	2 (40)	4 (36)	4 (33)
One or both parents deceased	3 (60)	6 (75)	2 (40)	0 (0)	5 (45)	6 (50)
Currently employed	5 (100)	4 (50)	3 (60)	1 (20)	5 (45)	8 (67)
Two or more lifetime sex partners	3 (100)^a^	6 (75)	5 (100)	5 (100)	8 (72)	8 (67)
Ever described partner abuse	0 (0)	7 (87)	2 (40)	0 (0)	4 (36)	5 (42)
Ever tested for HIV	3 (75)^a^	7 (87)	2 (40)	3 (60)	7 (64)	8 (67)
Ever RTI symptoms	2 (40)	3 (37)	2 (40)	3 (60)	2 (18)	8 (67)
Ever pregnant	1 (20)	7 (87)	2 (40)	4 (80)	6 (54)	8 (67)
Currently using condoms	5 (100)	2 (25)	3 (60)	2 (40)	6 (54)	6 (50)
Ever used hormonal contraception	2 (40)	2 (25)	2 (40)	0 (0)	1 (9)	5 (42)

a Some missing information due to lack of second or third interviews.

### Sexual relationship patterns

Sexual debut ranged from 13 to 18 years and was almost two years earlier, on average, for adolescents (14.8 years) than young women (16.6 years). For seven of the eight married participants, the first sexual partner was their husband, although sex often occurred before marriage. Regardless of risk context, first partners tended to be older (3–6 years) than participants, with several husbands older by as much as 11–17 years. No first sexual encounters were reported as physically forced – however, several participants felt they were tricked into having sex the first time.

Participants’ accounts of their current sexual relationships did not appear to vary by age, but did vary by sexual context. Only two of the eight participants who were or had been married described their husbands in positive terms. More commonly, they used terms such as “cruel” or “harsh,” or described husbands as prone to humiliate or insult them. All four of the married adolescents described being controlled and sometimes beaten by their husband. A 17-year-old who was currently staying at her mother's house admitted that “there was too much humiliation … A small mistake and he beats me.”

In contrast, sex workers and in-school youth tended to describe their relationships in more positive terms than the other participants. For example, an 18-year-old sex worker described her boyfriend as “not very capable” of much financial support but able to satisfy her sexually. She added “I am enjoying (sex) to a large extent; that is why we love each other.” A 21-year-old single university student described the emotional and material support she derived from her current partner, a non-Tanzanian businessman: “He is so caring; when I fall sick he takes me to the hospital; when I am hungry he cooks for me. So, he somehow treats me like a baby. … He gives me daily company.”

The word “trust” was often used to describe participants’ current relationships. The term, however, conferred multiple and sometimes contradictory meanings, including emotional and material support, and sexual discretion but rarely sexual exclusivity. A 20-year-old, who reported being married from the age of 13 or 14, was both doubtful and trustful of her husband's behaviour, “I trust my husband; if he has women outside, he will use a condom … fidelity is so important to me, because I trust someone – because he is not a player. He doesn't do dirty things … he is not just having sex with women. That is why I trust him.”

### Risk reduction behaviours

About half of the CFR participants (similar proportions of adolescents and young women) reported currently using condoms, although this varied widely by risk context. Only two of the eight married women used condoms with their husbands and about half of the unmarried in-school and out-of-school participants used them with one or more partners; most described using condoms to avoid pregnancy as well as disease. In contrast, all of the participants engaged in sex work described consistently using condoms, mostly to avoid HIV/STIs.

Adolescents and young adult women suggested that condom use was acceptable or even expected at the beginning of a new relationship. However, most participants described having stopped condom use within their own relationships as “trust” had been established. Several participants suggested that trust was more firmly established after both had tested negative for HIV. The majority of participants who had ever tested for HIV did so within the context of pregnancy or because of illness, although two students and two sex workers described more routine testing behaviour. Five married participants and one out-of-school participant said their partners refused testing.

Among those who were not sex workers, more-consistent condom use appeared to be for the purpose of preventing pregnancy. Only four participants reported currently using a modern, non-barrier method, including two injectable users and one participant each using oral pills and an implant. Several others reported having used or considered using an injectable previously, but stopped or did not initiate use out of fear of side effects.

### Risk perception

Most participants recognized they were at some risk of HIV, but about half appeared reluctant to attribute that risk directly to their own or their partner's behaviours. A 17-year-old married adolescent both trusted and did not trust her 20-year-old husband: “I think I can get HIV if my husband goes outside marriage, if he has sex with a person that is HIV positive, because I don't use protection … I don't use protection because I trust my husband.” To protect herself, she said that it was important “to be settled with my husband and not to have many men. Myself – I can settle, but I don't know about my partner.”

A few participants were more direct in their acknowledgement of risk. A 21-year-old university student admitted that, “Most of us young ladies get HIV because of having relationships for money or other material things, so that drives you to have reckless sex. For example, I can't face somebody and tell him ‘Let's go and test.’ I can't, I will just test myself, so that lack of confidence … leads to acquiring HIV.” Similarly, a sex worker stated, “I have told you my business; worrying is inevitable.” Several participants assessed their risk as minimal, because they took precautions. For example, a 20-year-old university student agreed that she was “of course concerned about AIDS, but not too concerned about other disease … because I protect myself …. Also, (I use) condoms and then I don't mix people. If I have one (partner), it's just him. Maybe if we breakup, that's when I go to another person.”

### SRH outcomes

Participants’ knowledge about sexually transmitted infections (STIs) was variable. Almost half of participants described having ever experienced reproductive tract symptoms, including itching, “fungus,” unusual vaginal discharge or painful intercourse. A few women appeared to use home remedies such as “Dettol” (an antiseptic liquid soap), although most sought treatment from a health facility. However, while participants described being provided medications for treatment, they rarely described being counselled about the source of infection or about ways to reduce sexual risk.

Unprotected sex and unplanned pregnancy were common among the CFR participants. More than half (14 of 23) had ever been pregnant – almost equally divided by age group. Only 7 of the 14 ever-pregnant participants had living children and two adolescent participants became pregnant during the CFR. Four participants, two in each age group, reported having aborted at least once.

### MCT phase

Adolescents and young adult women who participated in our MCT differed from the CFR qualitative sample in several important ways. First, they appeared to come from more stable homes, given that the majority of MCT participants (83% of adolescents and 58% of young adult women) were living with one or both parents. In addition, we enrolled few married women into the MCT. However, there were also striking similarities between the CFR and the MCT phases, especially in terms of sexual debut, patterns of sexual risk and risk reduction behaviours. Below, we describe the MCT participants and patterns of sexual risk, with a focus on whether and/or how adolescents differed from their adult counterparts. Table 2 describes the socio-demographic, sexual risk and protective factors by age group.

**Table 2:** Mock clinical trial: sexual risk and protective factors at baseline, by age group

	Adolescents (aged 15–17) (*N*=41)	Young women (aged 18–21) (*N*=94)	*p*
Age (mean)	16.4	19.3	
Years of education (mean)	8.4	8.9	0.18
Relationship status	%	%	
Married or cohabitating	2.4	9.6	
Regular partner, not cohabitating	97.6	79.8	0.02a
Single	0.0	10.6	
Living with one/both parents	% 82.9	% 58.5	0.006
Occupation	%	%	0.13
Paid employment	22.0	30.9	
Student	29.2	14.9	
Unemployed, not in school	48.8	54.2	
Religion	%	%	
Muslim	56.1	52.1	0.36^a^
Catholic	39.0	34.0	
Other	4.8	13.8	
Mean age at sexual debut	15.2	16.3	
Range	(11–17)	(10–20)	0.0002
First sexual encounter coerced/forced	34.2%	45.7%	0.21
First sexual partner 5+ years older	(*N*=36)	(*N*=87)	1.00^a^
	8.3%	10.3%	
Mean number of lifetime sex partners	1.7	2.6	
Range	(1–5)	(1–10)	0.002
Mean number of sexual partners per year of sexual exposure	1.24	1.06	0.24
Two or more partners in lifetime	39.0%	72.3%	<0.001
Ever concurrent partners	19.5%	16.0%	0.61
Ever exchanged sex for money or gifts	14.6%	13.8%	0.09
Ever tested for HIV	47.5%	75.0%	0.002
Ever had a pelvic exam	5.1%	15.7%	0.14^a^
Ever used modern contraceptives	2.4%	21.3%	0.004^a^
Current contraceptive use (non-condom)	0.0	14.9	0.006^a^
Had sex in last week	(*N*=41) 51.2%	(*N*=93) 49.5%	0.85
Condom at last sex	43.9%	50.0%	0.51
Alcohol at last sex	12.2%	8.5%	0.53^a^
Forced/coerced last sex	4.9%	8.5%	0.72^a^
	(*N*=41)	(*N*=93)	0.005^a^
Perceptions of HIV risk	%	%	
No perceived HIV risk	36.6	15.1	
Perceived a little HIV risk	58.5	64.5	
Perceived a lot of HIV risk	4.9	20.4	
Any incident SRH outcome^b^	(*N*=35) 22.9%	(*N*=69) 14.5%	0.29
Incident pregnancy	17.1%	10.1%	0.31
Diagnosis of bacterial vaginosis or trichomoniasis after baseline^b^	(*N*=22) 9.1%	(*N*=55) 5.5%	0.62^a^

a Fisher's exact test used for any cells with values of 5 or less

b subsample includes only those who had a month 4 clinic visit, during which pelvic and lab tests were conducted. RTI=reproductive tract infection; SRH=sexual and reproductive health; STI=sexually transmitted infection.

### Socio-demographics

Overall, the MCT sample of 135 participants was more similar to the group of single out-of-school CFR participants than to other risk contexts. They had similar years of education and work. About half of the participants were neither in school nor employed. Almost all adolescents (98%) and most young adult women (80%) were single, but had a regular partner.

### Sexual relationship patterns

As in the CFR, adolescents who joined the MCT reported similar or higher risks than their young adult counterparts. On average, adolescents initiated sex about six months earlier than did young adult participants (at 15.2 versus 16.3 years of age.) More than a third of participants, with similar proportions of adolescents and young adult women, reported their first sexual encounter to be coerced or forced. About 10% of participants’ first sexual partners were five or more years older than themselves.

At baseline, adolescents reported fewer sex partners over their lifetime (range, 1–5) than young adult women (range, 1–10). However, mean number of sexual partners was similar when adjusted for years of sexual exposure. About 14% of participants reported ever exchanging sex for money or goods, with no differences by age. There were no statistically significant differences in current sexual or substance use risks across age categories. Half of the participants in each age group had engaged in sex during the week prior to enrolment and about half reported using a condom at last sex. About 10% of participants reported using alcohol during their last sexual encounter; 4.9% of adolescents and 8.5% of adult participants reported that their last sexual encounter was coerced or forced.

### Risk reduction behaviours and risk perceptions

The rate of having ever used contraception was low; no adolescents and fewer than 15% of young women were currently using a modern contraceptive method. Condom use at last sex was similar between age groups. Although the difference was not statistically significant, fewer adolescents (about half) than young women (nearly two-thirds) reported any condom use in the past four weeks (data not shown).

Despite early sexual debut and some similarities in patterns of sexual risk, over a third of adolescents (36.6%) perceived themselves to be at no risk of HIV, compared to 15.1% of their counterparts.

### SRH outcomes

Over the six-month study period, there were 13 incident pregnancies (six in adolescents and seven in young women) among the 104 participants who had at least one follow-up visit and five confirmed cases of incident RTIs/STIs (three cases of BV and two cases of trichomoniasis) among the 78 participants who attended the four-month visit and completed lab tests. In addition, three cases of BV and five cases of trichomoniasis were identified and treated at enrolment. No participant was diagnosed more than once with an RTI/STI. Four young women and one adolescent were not enrolled in the MCT due to a positive HIV diagnosis at screening (data not shown). However, no incident HIV infections were detected post-enrolment. Although not statistically different, almost 20% of adolescents and 10% of young adult women became pregnant during this six-month study.

## Discussion

The findings from this study have several implications when considering adolescent participation in HIV prevention trials. First, sexually active adolescents in this study reported behaviours that put them at similar risk of HIV and other STIs as their adult counterparts. Adolescents in both phases initiated sex earlier than young adult women; had similar numbers of lifetime partners; and similar patterns of sexual concurrency, transactional sexual behaviour and alcohol use. Despite high levels of pregnancy and moderate levels of RTIs/STIs, use of condoms and other contraceptive methods was low or inconsistent for half or more of the participants, regardless of age. Given their sexual risk and barriers to using available risk reduction methods, it is important to determine whether new HIV prevention technologies would be safe, efficacious and acceptable to this population.

Nevertheless, adherence problems identified in two recent trials that aimed to evaluate the efficacy of new vaginal and/or oral HIV prevention methods in African women have raised concern about recruiting young women. Both the FEM-PrEP and the VOICE trials failed to produce evidence of effectiveness for products that were shown to be effective in other populations. Although neither trial recruited women below the age of 18, in the VOICE trial, young, single participants aged 18–24 were much more likely to contract HIV and much less likely to adhere to their study product than married women aged 25 and above [14]. In the FEM-PrEP trial, low adherence was attributed in part to women's low perception of HIV risk [21].

Indeed, the majority of young women – but especially adolescents who participated in our MCT perceived themselves to be at little or no risk of HIV. In that respect, those who joined our clinical study differed from the CFR sample. In particular, young married women and single, out-of-school women appeared particularly vulnerable. Most described sexual partners who were at times controlling or even abusive. They expressed concern about HIV risk, but had difficulty articulating actions they could take on their own to reduce that risk. Despite young married women's apparent need for new, women-initiated HIV prevention methods, the small number of married participants who enrolled in our study suggests additional challenges in recruiting this population.

This paper does not address issues related to the feasibility of adolescent recruitment and retention in HIV prevention trials, or their adherence to products. However, several South African studies examining the feasibility of recruiting adolescents into HIV biomedical and/or vaccine clinical trials found similar rates of incident pregnancy, STIs and HIV among adolescents below the age of 18 and adult women [11, 22]. While adolescents comprised a much smaller proportion of the overall sample in both studies (less than 20%), there were no differences in retention by age groups. A third South African study followed 100 HIV-negative adolescent women and men aged 14–17 over 12 months in preparation for HIV vaccine trials. Follow-up at one year was high (82%), but while incident pregnancy occurred among 7% of female participants, no incident HIV infections were diagnosed [23]. Finally, in a recent analysis of adolescent data from the Carraguard microbicide trial, young women aged 16–17 were no more likely than those aged 18–19 to miss visits, report missed doses or be lost to follow-up. However, they acknowledge that adolescent inclusion in clinical trials may require some modifications to trial design or implementation (i.e. training in youth-friendly counselling and further consideration of informed consent and compensation issues) to support their participation [24].

The study had several limitations. Originally, we aimed to conduct this study in two culturally diverse research sites, with an intended MCT sample size of 300 for subsequent regression analyses: 150 participants each from Pune, India, and Dar es Salaam, Tanzania. Unfortunately, the Indian site was closed after the CFR phase, largely due to difficulties obtaining approval from the local ethics committee (EC) for the MCT. The Indian EC restricted adolescent recruitment to married women only, with husband and/or parental consent required if below the age of 18. The Indian EC's stance further highlights the cultural dimensions that shape young women's sexual risk and their ability or inability to reduce risk by accessing HIV prevention information, products, services – and even clinical trials. Furthermore, observed differences across age groups on those sexual behaviour measures that summarize a participant's entire sexual history have ambiguous interpretations. For example, while older participants were more likely to report “ever” being tested for HIV, this difference might be expected since they are likely to have been sexually active for a longer period of time and also more likely to have become pregnant and tested within the context of antenatal care. And, since we did not collect data on the timing of these behaviours, we are unable to adjust for the unequal periods of risk across the two age groups. However, this limitation does not apply to the reported number of sex partners per year, questions relating to the first sexual experience, or to measures that refer to a behaviour within a specified time frame (e.g. sex in the past week or incident pregnancy). Finally, while the qualitative data from our CFR participants provide a richer understanding about the contexts within which young women experience risk, these data cannot be linked directly to MCT participants – who were recruited from the same venues, but through different approaches.

## Conclusions

Adolescents under age 18 are at risk of negative SRH outcomes, including pregnancy, STIs, RTIs and HIV. They are less likely than their older counterparts to report accessing HIV testing, obtaining gynaecological exams or using protective technologies such as contraception. With adolescents at similar, if not higher risk, than their young adult counterparts, combined with their underuse of current preventive services, their inclusion in clinical trials for new preventative technologies is clearly warranted – but additional support for recruitment, retention and product adherence may be required. Their access to new technologies such as vaginal microbicides or PrEP may be compromised unless research is undertaken to assess the safety, acceptability and effectiveness of these products in this age group. Adolescent exclusion from HIV prevention trials may hinder access to new HIV prevention technologies, once available.

## References

[1] World Health Organization, UNAIDS, UNICEF (2011). Global HIV/AIDS response: epidemic update and health sector progress towards universal access.

[2] Beyrer C, Abdool Karim Q (2013). The changing epidemiology of HIV in 2013. Curr Opin HIV AIDS.

[3] Tanzania Commission for AIDS (TACAIDS), Zanzibar AIDS Commission (ZAC), National Bureau of Statistics (NBS), Office of Chief Government Statistician (OCGS), Inc. II (2011–12). Tanzania HIV/AIDS and Malaria Indicator Survey.

[4] Mmbaga EJ, Leonard F, Leyna GH (2012). Incidence and predictors of adolescent's early sexual debut after three decades of HIV interventions in Tanzania: a time to debut analysis. PLoS One.

[5] Imanguli N (2008). Youth and the global HIV/AIDS pandemic.

[6] Maman S, Mbwambo JK, Hogan NM, Kilonzo GP, Campbell JC, Weiss E (2002). HIV-positive women report more lifetime partner violence: findings from a voluntary counseling and testing clinic in Dar es Salaam, Tanzania. Am J Public Health.

[7] Maganja RK, Maman S, Groves A, Mbwambo JK (2007). Skinning the goat and pulling the load: transactional sex among youth in Dar es Salaam, Tanzania. AIDS Care.

[8] Yamanis TJ, Doherty IA, Weir SS, Bowling JM, Kajula LJ, Mbwambo JK (2013). From coitus to concurrency: sexual partnership characteristics and risk behaviors of 15–19 year old men recruited from urban venues in Tanzania. AIDS Behav.

[9] Urassa W, Moshiro C, Chalamilla G, Mhalu F, Sandstrom E (2008). Risky sexual practices among youth attending a sexually transmitted infection clinic in Dar es Salaam, Tanzania. BMC Infect Dis.

[10] Desai J, Katz K, Janowitz B (2005). Iringa Youth Behavioral Survey: findings and report.

[11] Abdool Karim Q, Kharsany ABM, Frohlich JA, Werner L, Mlotshwa M, Madlala BT (2012). HIV incidence in young girls in KwaZulu-Natal, South Africa-public health imperative for their inclusion in HIV biomedical intervention trials. AIDS Behav.

[12] Skoler-Karpoff S, Ramjee G, Ahmed K, Altini L, Plagianos MG, Friedland B (2008). Efficacy of Carraguard for prevention of HIV infection in women in South Africa: a randomised, double-blind, placebo-controlled trial. Lancet.

[13] Nunn A, McCormack S, Crook AM, Pool R, Rutterford C, Hayes R (2009). Microbicides Development Programme: design of a phase III trial to measure the efficacy of the vaginal microbicide PRO 2000/5 for HIV prevention. Trials.

[14] Rossi L Understanding the results of VOICE2013.

[15] DiClemente RJ, Ruiz MS, Sales JM (2010). Barriers to adolescents’ participation in HIV biomedical prevention research. J Acquir Immune Defic Syndr.

[16] MacQueen KM, Karim QA (2007). Practice brief: adolescents and HIV clinical trials: ethics, culture, and context. J Assoc Nurses in AIDS Care: JANAC.

[17] Pomfret S, Abdool Karim Q, Benatar SR (2010). Barriers to adolescents’ participation in HIV biomedical prevention research. J Acquir Immune Defic Syndr.

[18] Essack Z, Slack C, Strode A (2008). Overcoming key obstacles to adolescent involvement in HIV vaccine & microbicide trials: a roadmap for stakeholders. HIV AIDS Vaccines Ethics Group in collaboration with the Global Campaign for Microbicides.

[19] Jaspan HB, Berwick JR, Myer L, Mathews C, Flisher AJ, Wood R (2006). Adolescent HIV prevalence, sexual risk, and willingness to participate in HIV vaccine trials. J Adolesc Health.

[20] Ulin PR, Robinson ET, Tolley EE (2006). Qualitative methods in public health: a field guide for applied research.

[21] Van Damme L, Corneli A, Ahmed K, Agot K, Lombaard J, Kapiga S (2012). Preexposure prophylaxis for HIV infection among African women. New Engl J Med.

[22] Middelkoop K, Myer L, Mark D, Mthimunye SP, Smit J, Wood R (2008). Adolescent and adult participation in an HIV vaccine trial preparedness cohort in South Africa. J Adolesc Health.

[23] Jaspan HB, Flisher AJ, Myer L, Mathews C, Middelkoop K, Mark D (2011). Sexual health, HIV risk, and retention in an adolescent HIV-prevention trial preparatory cohort. J Adolesc Health.

[24] Schenk KD, Friedland BA, Chau M, Stoner M, Plagianos MG, Skoler-Karpoff S (2014). Enrollment of adolescents aged 16–17 years old in microbicide trials: an evidence-based approach. J Adolesc Health.

# Sub article: Communicating about microbicides with women in mind: tailoring messages for specific audiences

**DOI:** 10.7448/IAS.17.3.19151

**Published:** 2014-09-08

**Authors:** Sekou Sidibe, Allison P Pack, Elizabeth E Tolley, Elizabeth Ryan, Caroline Mackenzie, Emily Bockh, George Githuka

**Affiliations:** 1 Social & Behavioural Health Sciences, FHI360, Durham, NC, USA; 2 Social Marketing and Communication, FHI 360, Washington, DC, USA; 3 Research, FHI 360, Nairobi, Kenya; 4 National AIDS and STI Control Programme, Nairobi, Kenya

**Keywords:** microbicide, communication, women, HIV prevention, messages, Kenya

## Abstract

**Introduction:**

Current HIV prevention options are unrealistic for most women; however, HIV prevention research has made important strides, including on-going development of antiretroviral-based vaginal microbicide gels. Nevertheless, social-behavioural research suggests that women's ability to access and use new HIV prevention technologies will be strongly influenced by a range of socio-cultural, gender and structural factors which should be addressed by communications and marketing strategies, so that these products can be positioned in ways that women can use them.

**Methods:**

Based on an extensive literature review and in-country policy consultation, consisting of approximately 43 stakeholders, we describe barriers and facilitators to HIV prevention, including potential microbicide use, for four priority audiences of Kenyan women (female sex workers [FSWs], women in stable and discordant relationships, and sexually active single young women). We then describe how messages that position microbicides might be tailored for each audience of women.

**Results:**

We reviewed 103 peer-reviewed articles and reports. In Kenya, structural factors and gender inequality greatly influence HIV prevention for women. HIV risk perception and the ability to consistently use condoms and other prevention products often vary by partner type. Women in stable relationships find condom use challenging because they connote a lack of trust. However, women in other contexts are often able to negotiate condom use, though they may face challenges with consistent use. These women include FSWs who regularly use condoms with their casual clients, young women in the initial stages of a sexual relationship and discordant couples. Thus, we consider two approaches to framing messages aimed at increasing general awareness of microbicides – messages that focus strictly on HIV prevention and ones that focus on other benefits of microbicides such as increased pleasure, intimacy or sexual empowerment, in addition to HIV prevention.

**Conclusions:**

If carefully tailored, microbicide communication materials may facilitate product use by women who do not currently use any HIV prevention method. Conversely, message tailoring for women with high-risk perception will help ensure that microbicides are used as additional protection, together with condoms.

## Introduction

In Kenya, HIV prevalence has fallen from 7.2% in 2007 to 5.6% in 2012 among adults aged 15–64 [1, 2]. However, women continue to be disproportionally affected by HIV; prevalence rates were 6.9% among women, compared to 4.4% among men [1]. In addition, approximately 40% of new infections have occurred within stable couples [1]. Women's need for new HIV prevention technologies remains a priority.

Recent trials have demonstrated the effectiveness of new antiretroviral (ARV)-based HIV prevention products. Oral pre-exposure prophylactic (PrEP) use of tenofovir or Truvada (tenofovir and emtricitibine) was shown to reduce HIV transmission in heterosexual discordant couples (by 62% for tenofovir-only and 73% for Truvada) [3–5]. Additionally, the CAPRISA 004 clinical trial in South Africa produced a proof of concept for peri-coital vaginal use of 1% tenofovir gel in reducing HIV transmission to heterosexual women [6].

While oral PrEP has been approved for use in high-risk populations in the United States [7], decisions about vaginal microbicide gel may still be several years away as regulators await confirmatory results from the FACTS 001 trial. Furthermore, despite commonalities between oral and vaginal ARV-based products for HIV prevention, their introduction strategies may require different approaches.

## The need for tailored introduction strategies

Social-behaviour research conducted alongside microbicide clinical trials suggests women's acceptability of new HIV prevention technologies, and their ability to access and use them, will be strongly influenced by the way products are positioned by communication and marketing strategies [8]. For messages to be effective, they must be tailored; different audiences will perceive different sources of risk, outcome expectations and barriers to action. For example, previous research suggests women's ability to identify their HIV risk varies considerably by geographic and sexual relationship context [9–12]. Research globally has associated low rates of condom use, especially among women in stable relationships, with the perception that condoms are primarily for HIV prevention. And, because HIV is associated with high-risk sexual behaviour, the request for condom use within a stable relationship connotes unfaithfulness. Female condom programmes have worked to reverse such connotations by emphasizing the use of female condoms for contraception and by promoting their use in loving relationships [13]. It will be equally important to develop and test potential ways to position microbicide use to avoid stigmatization.

Some researchers and advocates have suggested that vaginal microbicide gel might be less stigmatized if it is positioned as a sexual lubricant [14]. However, although the notion that gel use increases sexual pleasure could be positively perceived by some, it may be negatively perceived by others. Similarly, if gel is promoted for use among audiences in which condom use is a norm, communication strategies should model dual use of gel and condoms to avoid the substitution of more efficacious condoms with less efficacious microbicide products. Therefore, introduction strategies, including the content and framing of messages, will need to be tailored to various audiences if new technologies are to be widely accessed and used.

*Communicating about Microbicides with Women in Mind* is a three-phase project to assist Kenyan policymakers, programme implementers and advocacy groups in planning for future introduction of ARV-based vaginal microbicide gel, once proven effective. Specifically, the project aims to develop a suite of communication materials tailored to the needs of potential users and to assess the effects of message framing on interest in microbicide use, negative attitudes towards microbicides and potential condom migration.

In this paper, we draw on an extensive literature review and in-country policy consultation, conducted during the first project phase, to describe four potential audiences who might benefit from microbicide use. We also discuss how communication materials might require tailoring for each audience.

## Methods

### Literature search strategy

During the first phase, we systematically searched peer-reviewed articles, from January to July 2012, using PubMed and Google Scholar. We sought peer-reviewed articles of academic quality that identified barriers and facilitators to HIV prevention among four potential end-user audiences representing different sexual relationship contexts: female sex workers (FSWs), women in stable relationships, women in discordant relationships, and sexually active, single young women. These priority audiences were selected based on their high HIV prevalence rates and previous research of key populations who might benefit from vaginal microbicide use [6, 9, 10]. Other data sources included national surveillance system data reports, such as the AIDS indicator surveys and demographic health surveys.

The search included medical subject headings (MeSH) terms for HIV and AIDS, terms associated with each of the four priority audiences (“sex work,” “FSWs,” “couples,” “serodiscordant couples,” “young women,” “women,” “youth,” and “adolescents”), and key terms associated with HIV prevention (“HIV testing,” “condoms,” “microbicides,” “PrEP,” “sexual relationships,” “communication,” “discussion,” “negotiation,” “HIV risk perception,” “sexual pleasure,” “sexual practices,” “sexual power,” “gender-based violence/coercion,” “stigma,” “education,” “social support,” “money,” “substance use,” and “alcohol use”).

### Inclusion and exclusion criteria

Studies of any design were eligible for inclusion if they addressed HIV prevention among one or more of the specified audiences and if they were published between 1990 and 2012. Articles were also included in the analysis if they reported findings from Kenya or neighbouring countries, including Uganda, Rwanda and Tanzania. Several multi-site studies in sub-Saharan African were also included when Kenya was one of the prioritized countries. All surveillance reports specific to Kenya were included in the analysis. Articles and reports were excluded if they did not focus on any of the priority audiences, HIV prevention, or Kenya and its neighbouring countries (Figure 1).

**Figure 1:** Flow chart of included articles and reports.

### Analysis

The search yielded 103 unique articles and reports (Table 1), 96 of which were specific to Kenya; the remaining pertained to neighbouring sub-Saharan African countries. Several articles (*n*=27) included information on men and young boys; they were retained because they contained information relevant to the priority audiences.

**Table 1:** Geographical focus of the articles

Geographical focus	Sex workers	Young women	Stable relationships	Discordant couples	Other groups	Total (%)
Kenya	38	25	7	5	21	96 (93.20)
Sub-Saharan Africa	2	0	0	0	5	7 (6.79)
Total	40	25	7	5	27	103 (100)

Articles and reports were uploaded into NVivo 9.2, read for content and coded to identify 26 broad themes, including risk perception, sexual practices, communication and condom use as well as priority audiences. Articles and reports were categorized and analysed by audiences based on specific themes; those containing data on multiple audiences were coded for each relevant audience. Data pertaining to multiple audiences or themes were also double coded. For each main theme, we created an Excel matrix organized by article, with up to three illustrative excerpts across the columns, and colour-coded by audience group. These thematic matrices helped identify similarities and differences among audiences in terms of HIV prevention needs and challenges.

Data were presented and discussed during a two-day national policy consultation, conducted in Naivasha, Kenya, in collaboration with the National AIDS and STI Control Programme and the Kenya Medical Research Institute. Participants included 43 stakeholders comprising Kenyan policymakers, programme managers and civil society advocates, as well as representatives from international non-governmental organizations (NGOs) and funding agencies. The potential introduction of ARV-based HIV prevention in Kenya, including various priority audiences for microbicide communication materials, was discussed.

## Results

### Literature review

The varying social contexts within which women and their partners live, work and love create unique challenges for HIV prevention efforts (Table 2). The number and type of partners, in large part influenced by structural factors and gender inequality [15], affect women's HIV risk perception and their ability to respond to that risk [16]. For example, the ability to use condoms consistently and correctly often varies by partner type [9, 17, 18]. As Kenya considers microbicide introduction, understanding current HIV prevention behaviour and related sexual practices in Kenya may prove useful in anticipating potential barriers and facilitators to new product use for various audiences.

**Table 2:** Focused, summary results from the literature review by audience

Female sex workers	Women in stable relationships
Partner information • Partners include clients, regular partners, boyfriends, husbands – of unknown status • Low decision-making power with regular partners • Sex is often planned • Potential for violence HIV risk perception and information source • High-risk perception • Receive HIV information from peer educators Condom use • High condom use with clients • Low condom use with regular partners Microbicide considerations • Lubrication could facilitate condom use • Some perceive that clients prefer dry sex • Multiple partners, substance use might affect adherence • Vaginal washing might reduce effectiveness	Partner information • Some have one regular partner of unknown status; others have additional partners of unknown status • Low decision-making power in general • Potential for violence HIV risk perception and information source • Low-risk perception • Receive HIV information and testing in antenatal care settings Condom use • Some condom use at start of relationship • Low/no condom use once committed Microbicide considerations • Partner involvement shown to improve adherence • High acceptability among married trial participants • Some concerns about stigma; may imply infidelity
**Discordant couples**	**Single young women (15 to 24 years)**

Partner information • Some have one regular partner only; others have additional partners of unknown status • Low decision-making power with additional partners • Potential for violence HIV risk perception and information source • Low-risk perception prior to testing and disclosure, but high-risk perception after testing, disclosure • Receive HIV information from health system Condom use • High condom use with negative partner, if relationship continues • Low condom use with additional partners Microbicide considerations • Could protect discordant couples who wish to conceive • May be useful for HIV-negative women before HIV-positive partner initiates treatment	Partner information • Partners can include transactional, cross-generational sex partners, regular partners, boyfriends – of unknown status • Low decision-making power in general • Sex is not often planned • Potential for violence HIV risk perception and information source • Low-risk perception • Receive HIV information from schools and youth-friendly clinics and peers, but information is limited Condom use • Some condom use at start of intimate relationship • Low condom use in general Microbicide considerations • Interest in new, effective products • Concern about use disclosure • Some concern about product insertion, adherence

#### Female sex workers

The 2012 Most-at-Risk Populations (MARPs) Surveillance Report estimates that the HIV prevalence rate among FSWs in Kenya is between 24 and 50% [19]. Several Kenyan studies [19–24] suggest that FSWs work within a culture of violence, commonly triggered by substance use and discussions of condom use and payment. The illegal nature of sex work perpetuates discrimination, deterring FSWs from reporting the violence or seeking assistance from law enforcement [20–21]. Moreover, economic necessity negatively affects FSWs’ ability to negotiate condom use; inconsistent use often results from a client's willingness to pay more for unprotected sex [9, 25].

Nevertheless, HIV risk perception is high among many sex workers [16, 25], and many are able to respond to their risk. FSWs report considerable awareness of STI symptoms, high rates of treatment-seeking behaviours, HIV testing and subsequent knowledge of HIV status [9, 18]. A number of studies conducted across Kenya report high rates of consistent condom use among FSWs [9, 24, 26].

Yet, condom use among FSWs is strongly associated with partner type [9, 16, 18, 27, 28]; higher rates of condom use are reported with casual partners or clients than with regular or primary partners. Some studies suggest that for FSWs, primary partners are considered less risky than casual partners [23, 28], a theme which is consistent with women in other contexts.

One study assessing vaginal microbicide gel acceptability among FSWs revealed that gel could be promoted as a lubricant to facilitate condom use, thus enhancing protection [10]. However, a study assessing intravaginal practices and implications for new HIV prevention products among FSWs in urban Kenya revealed that vaginal washing practices, often influenced by perceived or real male preference for “dry sex,” and anal sex were also common [28, 29]. Such practices may reduce the effectiveness of vaginal microbicides.

#### Women in stable relationships

According to the 2012 KAIS, HIV prevalence among women in stable relationships was estimated to be 5.3% [1]. Moreover, an estimated 44% of new infections are thought to occur within stable relationships, according to the 2009 Kenya HIV Response and Modes of Transmission Analysis [30]. Studies suggest that women in stable relationships perceive their risk for HIV to be low [22, 31] and are therefore rarely tested for HIV outside of antenatal contexts [32]; as part of the 2007 KAIS, most women (about 60%) reported being tested within the context of antenatal services [2].

Within stable relationships, men and women report different patterns of sexual behaviour. While women report approximately two lifetime partners, men report more than seven. Similarly, concurrent relationships were reported by 1% of women in monogamous relationships and 2.6% of women in polygamous relationships, compared to 7 and 10% of men [2].

Kenyan men are important decision makers in sexual matters, from decisions about the number and timing of children to the use of contraceptive and disease prevention methods. In contrast, women have been socialized to comply with their partner's desires for sex and pleasure [32].

Women in stable relationships face barriers to requesting condom use, largely because such requests raise suspicions of unfaithfulness and increase the potential for violence [33–36]. And, while condom use for contraception appears more acceptable than for disease prevention, less than 2% of women in stable relationships report using condoms for contraception [37]. Cross-sectional studies across Kenya report low spousal communication related to HIV prevention, further suggesting that women in stable relationships have little ability to negotiate condom use or other safe sex practices [35–37].

#### Discordant couples

According to the KAIS 2012, there are an estimated 260,000 HIV-discordant couples in Kenya [1]. Among couples in which both partners received HIV test results, 5% were identified as HIV-discordant and 3% were concordant HIV-positive [1]. Yet, risk perception is generally low for these couples before testing. Indeed, 53% of men and women who were tested as part of the 2012 KAIS and found to be in HIV-discordant relationships were unaware of their status [1]. Similarly, a study conducted in Mombasa revealed that women were unaware that their primary partners were involved in other relationships [38].

Before diagnosis, women and men in discordant relationships are like other stable couples in which women's fidelity and fertility are highly valued. In fact, fertility continues to be important even within HIV-affected relationships. The 2007 KAIS reported that about one-fourth of women who self-declared as HIV positive reported wanting a/another child [2], yet fear of stigma is thought to limit the use of necessary PMTCT services for HIV-positive women [39–41].

Fear of stigma has also been found to inhibit HIV status disclosure between partners, regardless of relationship length. However, once discordancy has been disclosed, if couples choose to remain together, both HIV risk perception and the ability to negotiate risk reduction behaviours can increase with effective HIV counselling [11].

#### Single young women

An estimated 5.6% of sexually active young women aged 15–24 were identified as HIV positive in the 2007 KAIS study [2]. Among young women who participated in the 2012 KIAS, 66% reported ever having sex [1]; other studies suggest that women initiate sex at young ages and that sexual activity often begins within the first month of a new relationship [12, 42].

Despite widespread reports of multiple concurrent partnerships among Kenyan youth [17, 43, 44], HIV risk perception and subsequent condom use is low for young women [45, 46]; only 11% reported consistent condom use in the 2012 KAIS [1]. As with women in other contexts, condom use varies by partner type; it primarily occurs at the start of a relationship when risk is perceived to be high [12, 43] and is abandoned once “trust” is established [43]. Young women's ability to negotiate condom use is also compromised by gender, age and economic disparities, especially in situations involving cross-generational and transactional sex [12, 46, 47]. While some use these exchanges to meet basic needs [43, 48, 49], others are motivated by the potential for supplementary gifts [46, 49].

Misinformation about HIV transmission is pervasive among many young women [17, 46–50]. Despite lessons on life-skills, teachers in Kisumu and Nakuru have reported feeling unprepared to talk about sexuality in the classroom, in part, due to parental concerns [51]. Similarly, cultural taboos about adolescent sexuality pose barriers to purchasing condoms, negotiating condom use with partners [48, 52], or visiting a health centre for HIV and STI testing. However, providing an increase in youth-friendly HIV testing and counselling centres in Kisumu resulted in an increase in HIV testing [44].

Montandon *et al*. qualitatively examined the potential acceptability and use of microbicides among young women in Kisumu [49] in 2008. This study suggested that the unplanned nature of adolescent sexual activity, a fear of vaginally inserting or trying new, “experimental” products, and concerns about product efficacy will likely impede vaginal microbicide use for young women [49].

#### Policy consultation

Results from the literature review, shared at the Naivasha consultation, provided context for participants to prioritize audiences they felt would be most suited for microbicide communication materials. With financial and human resources likely to be a challenge for new product implementation, some participants felt it would be important to prioritize known risk groups (FSWs and/or HIV-negative women in discordant couples) already identified as a national priority for HIV prevention.

However, participants also expressed concern about the effects of stigma, and whether focusing on known risk groups would inadvertently lead to the stigmatization and subsequent non-use of microbicides by other women. Some participants felt strongly that while women in stable relationships and young women may not typically be considered most-at-risk, current HIV prevention options are not realistic for them.

Consequently, a group of known high-risk women, (FSWs) and two groups of at-risk women who have not traditionally been targeted with HIV prevention information (single young women aged 15–24 and women in stable relationships) were identified as primary audiences for the *Communicating about Microbicides with Women in Mind* project. Men and health care providers were identified as secondary audiences.

Finally, meeting participants also emphasized that civil society organizations should work closely with the government to develop appropriate procedures and regulations for microbicide introduction. They agreed the government should drive the agenda for the introduction of ARV-based HIV prevention, including the development of national guidelines. A project advisory committee (PAC), consisting of key governmental and non-governmental stakeholders, was therefore established to guide the *Communicating about Microbicides with Women in Mind* project.

## Discussion

Social marketing and communication strategies can potentially increase the uptake of new HIV prevention technologies, including microbicides. UNAIDS has long acknowledged the role of social marketing as an effective tool in the global response to HIV/AIDS [53]. In a review of more than 6500 studies, PSI conducted an analysis of social marketing approaches and programmes, demonstrating the effectiveness of social marketing for changing health behaviours. Twenty studies demonstrated increases in HIV risk perception, knowledge, and self-efficacy; 18 demonstrated increases in condom use and HIV testing; and eight demonstrated reductions in HIV prevalence [54]. However, the design and implementation of effective strategies relies on a solid understanding of priority audiences and the many factors that influence their behaviours. This information determines the most effective way of positioning products to generate interest in use.

Because vaginal microbicides are likely to be partially effective, they may need to be promoted within the context of condom use. Experience from programmes that have promoted dual protection against pregnancy and HIV may be useful. Unfortunately, there have been few quality studies documenting the effectiveness of behavioural interventions for promoting dual protection. Yet, many organizations that have implemented dual protection interventions have documented lessons learned; these include the need to tailor messages to specific HIV contexts and the fact that a dual protection message is not always appropriate [55].

Microbicide introduction strategies will need to consider how to position microbicides so they:

1. Can be used by all types of sexually active women
2. Do not connote lack of trust or infidelity
3. Are used when condom use is not possible or as a back-up for condoms
4. Do not replace condoms when condom use is possible

To this end, it will be important to determine whether microbicide awareness-raising materials, should focus exclusively on HIV prevention or more prominently on other benefits such as sexual pleasure, intimacy, and/or female empowerment. Which type of framing generates the most interest in microbicides will likely differ depending on the target audience. At the same time, it will be important to avoid linking microbicides to stigmatizing behaviours such as unfaithfulness or sex work. And finally, because microbicides are currently not as effective as condoms, materials must avoid promoting them in a way that would result in decreased condom use among those who use condoms with all or some of their partners.

Widespread use of microbicides will require more than awareness, however; it will also require intensive counselling about risks, benefits, feasibility and correct use of microbicides. Educational materials, tailored for specific audiences, offer an opportunity for more detailed discussion about these topics and potential adherence challenges. Educational materials designed for FSWs and HIV-negative women in discordant relationships, for example, will need to emphasize the importance of combining microbicides with condom use, as opposed to substituting condom use with microbicides. As indicated by the literature, FSWs have high-risk perception and an ability to negotiate condom use with clients, yet they are also economically motivated and may view condom-less sex as an opportunity to obtain additional cash. Materials will need to deter this behaviour and promote dual use of microbicides and condoms as enhancing protection and potentially facilitating condom use.

Moreover, materials for FSWs will need to address various contexts in which microbicides may be used, including with primary partners. Because sex is often anticipated for these women, materials might want to encourage daily or routine use of microbicides. And finally, materials will need to address various sexual practices reportedly common among some FSWs, including vaginal douching and anal sex.

For young women and women in stable relationships, however, educational materials will need to acknowledge that partial protection from microbicides is better than no protection at all. Moreover, materials should provide detailed information on HIV risk and prevention options. As indicated by the literature, despite widespread reports of concurrent partnerships, many of these women do not perceive themselves to be at risk and have not used, or do not currently use, condoms with their partners. Likewise, with many young women and women in stable relationships reporting difficulty negotiating safe sex with their partners, educational materials should provide guidance to ensure women can decide whether and/or how they should involve their partners in discussions about microbicide use.

For young women, in particular, materials will need to address the fact that many of them have limited experiences with reproductive health services, may fear necessary HIV testing or the insertion of new vaginal products, and often do not plan for sex.

Limitations for this project include the fact that there are currently few studies available on microbicide acceptability in Kenya and HIV prevention among women in stable relationships and women in discordant couples. Moreover, without an available product, much of the work we have conducted is based on hypothetical assessments.

## Conclusions

Findings from this project address a critical gap in our current approach to HIV prevention communication by providing evidence-based recommendations that should be considered for communication materials in order to support the future introduction of vaginal microbicide gels in Kenya. We believe that these recommendations can be used to create effective materials and will contribute to increased use of microbicides by women in a variety of sexual contexts – including young women and those in stable relationships – who have not traditionally been considered at high risk of HIV, but may actually be some of the most-at-risk.

## References

[1] National AIDS and STI Control Programme (2013). Kenya AIDS indicator survey 2012: preliminary report.

[2] National AIDS and STI Control Programme (2008). Kenya AIDS indicator survey 2007.

[3] Celum C (2011). HIV preexposure prophylaxis: new data and potential use. Top Antivir Med.

[4] Baeten JM, Donnell D, Ndase P, Mugo NR, Campbell JD, Wangisi J (2012). Antiretroviral prophylaxis for HIV prevention in heterosexual men and women. N Engl J Med.

[5] Grant RM, Lama JR, Anderson PL, McMahan V, Liu AY, Vargas L (2010). Preexposure chemoprophylaxis for HIV prevention in men who have sex with men. N Engl J Med.

[6] Karim QA, Karim SSA, Frohlich JA, Grobler AC, Baxter C, Mansoor LE (2010). Effectiveness and safety of tenofovir gel, an antiretroviral microbicide, for the prevention of HIV infection in women. Science.

[7] Liu A, Cohen S, Follansbee S, Cohan D, Weber S, Sachdev D (2014). Early experiences implementing pre-exposure prophylaxis (PrEP) for HIV prevention in San Francisco. PLoS Med.

[8] Becker J, Dabash R, McGory E, Copper D, Harries J, Hoffman M (2004). Paving the path: preparing for microbicide introduction report of a qualitative study in South Africa.

[9] Ngugi E, Benoit C, Hallgrimsdottir H, Jansson M, Roth EA (2012). Partners and clients of female sex workers in an informal urban settlement in Nairobi, Kenya. Cult Health Sex.

[10] Greene E, Batona G, Hallad J, Johnson S, Neema S, Tolley EE (2010). Acceptability and adherence of a candidate microbicide gel among high-risk women in Africa and India. Cult Health Sex.

[11] Benki-Nugent S, Chung MH, Ackers M, Richardson BA, McGrath CJ, Kohler P (2011). Knowing a sexual partner is HIV-1-uninfected is associated with higher condom use among HIV-1-infected adults in Kenya. Sex Transm Dis.

[12] Luke N, Goldberg RE, Mberu BU, Zulu EM (2011). Social exchange and sexual behavior in young women's premarital relationships in Kenya. J Marriage Fam.

[13] UNFPA (2011). HIV prevention gains momentum: successes in female condom programming.

[14] Montgomery CM, Lees S, Stadler J, Morar NS, Ssali A, Mwanza B (2008). The role of partnership dynamics in determining the acceptability of condoms and microbicides. AIDS Care.

[15] Chiao C, Mishra V, Ksobiech K (2011). Spousal communication about HIV prevention in Kenya. J Health Commun.

[16] McClelland L, Wanje G, Kashonga F, Kibe L, McClelland RS, Kiarie J (2011). Understanding the context of HIV risk behavior among HIV-positive and HIV-negative female sex workers and male bar clients following antiretroviral therapy rollout in Mombasa, Kenya. AIDS Educ Prev.

[17] Westercamp N, Mattson CL, Madonia M, Moses S, Agot K, Ndinya-Achola JO (2010). Determinants of consistent condom use vary by partner type among young men in Kisumu, Kenya: a multi-level data analysis. AIDS Behav.

[18] Matovu JK, Ssebadduka BN (2012). Sexual risk behaviours, condom use and sexually transmitted infection treatment-seeking behaviours among female sex workers and truck drivers in Uganda. Int J STD AIDS.

[19] National AIDS and STI Control Programme (2012). Most-at-risk populations: unveiling new evidence for accelerated programming in MARPS surveillance report.

[20] Okal J, Chersich MF, Tsui S, Sutherland E, Temmerman M, Luchters S (2011). Sexual and physical violence against female sex workers in Kenya: a qualitative enquiry. AIDS Care.

[21] FIDA Kenya, The Federation of Women Lawyers (2008). Documenting human rights violation of sex workers in Kenya.

[22] Ochako R, Ulwodi D, Njagi P, Kimetu S, Onyango A (2011). Trends and determinants of comprehensive HIV and AIDS knowledge among urban young women in Kenya. AIDS Res Ther.

[23] Thomsen SC, Ombidi W, Toroitich-Ruto C, Wong EL, Tucker HO, Homan R (2006). A prospective study assessing the effects of introducing the female condom in a sex worker population in Mombasa, Kenya. Sex Transm Infect.

[24] Tegang SP, Abdallah S, Emukule G, Luchters S, Kingola N, Barasa M (2010). Concurrent sexual and substance-use risk behaviours among female sex workers in Kenya's Coast Province: findings from a behavioural monitoring survey. SAHARA J.

[25] Okal J, Luchters S, Geibel S, Chersich MF, Lango D, Temmerman M (2009). Social context, sexual risk perceptions and stigma: HIV vulnerability among male sex workers in Mombasa, Kenya. Cult Health Sex.

[26] Bukusi EA, Gallo MF, Sharma A, Njoroge B, Jamieson DJ, Nguti R (2009). Adherence to diaphragm use for infection prevention: a prospective study of female sex workers in Kenya. Infect Dis Obstet Gynecol.

[27] Hawken MP, Melis RDJ, Ngombo DT, Mandaliya K, Price J, Dallabetta G (2002). Part time female sex workers in a suburban community in Kenya: a vulnerable hidden population. Sex Transm Infect.

[28] Priddy FH, Wakasiaka S, Hoang TD, Smith DJ, Farah B, del Rio C (2011). Anal sex, vaginal practices, and HIV incidence in female sex workers in urban Kenya: implications for the development of intravaginal HIV prevention methods. AIDS Res Hum Retroviruses.

[29] Smith DJ, Wakasiaka S, Hoang TD, Bwayo JJ, Del Rio C, Priddy FH (2008). An evaluation of intravaginal rings as a potential HIV prevention device in urban Kenya: behaviors and attitudes that might influence uptake within a high-risk population. J Womens Health (Larchmt).

[30] National AIDS Control Council (2009). Kenya HIV prevention response and modes of transmission analysis.

[31] Taegtmeyer M, Kilonzo N, Mung'ala L, Morgan G, Theobald S (2006). Using gender analysis to build voluntary counselling and testing responses in Kenya. Trans R Soc Trop Med Hyg.

[32] Akwara PA, Madise NJ, Hinde A (2003). Perception of risk of HIV/AIDS and sexual behaviour in Kenya. J Biosoc Sci.

[33] Kaiser R, Bunnell R, Hightower A, Kim AA, Cherutich P, Mwangi M (2011). Factors associated with HIV infection in married or cohabitating couples in Kenya: results from a nationally representative study. PLoS One.

[34] Muturi NW (2005). Communication for HIV/AIDS prevention in Kenya: social-cultural considerations. J Health Commun.

[35] Miller AN, Golding L, Ngula KW, Wambua M, Mutua E, Kitizo MN (2009). Couples’ communication on sexual and relational issues among the Akamba in Kenya. Afr J AIDS Res.

[36] Neville AN, Rubin DL (2007). Factors leading to self-disclosure of a positive HIV diagnosis in Nairobi, Kenya: people living with HIV/AIDS in the Sub-Sahara. Qual Health Res.

[37] Kenya National Bureau of Statistics, ICF Macro (2010). Kenya demographic and health survey 2008–09.

[38] Sarna A, Chersich M, Okal J, Luchters SM, Mandaliya KN, Rutenberg N (2009). Changes in sexual risk taking with antiretroviral treatment: influence of context and gender norms in Mombasa, Kenya. Cult Health Sex.

[39] Were E, Wools-Kaloustian K, Baliddawa J, Ayuo PO, Sidle J, Fife K (2008). Stakeholders perception of HIV sero-discordant couples in western Kenya. East Afr Med J.

[40] Awiti Ujiji O, Ekström AM, Ilako F, Indalo D, Wamalwa D, Rubenson B (2011). Reasoning and deciding PMTCT-adherence during pregnancy among women living with HIV in Kenya. Cult Health Sex.

[41] Miller NA, Rubin DL (2007). Factors leading to self-disclosure of a positive HIV diagnosis in Nairobi, Kenya: people living with HIV/AIDS in the Sub-Sahara. Qual Health Res.

[42] Maticka-Tyndale E, Kyeremeh C (2010). The trouble with condoms: norms and meanings of sexuality and condom use among school-going youth in Kenya. Int J Sex Health.

[43] Njue C, Voeten HA, Remes P (2011). Porn video shows, local brew, and transactional sex: HIV risk among youth in Kisumu, Kenya. BMC Public Health.

[44] Kabiru CW, Luke N, Izugbara CO, Zulu EM (2010). The correlates of HIV testing and impacts on sexual behavior: evidence from a life history study of young people in Kisumu, Kenya. BMC Public Health.

[45] Ngom P, Magadi M, Owuor T (2003). Parental presence and adolescent reproductive health among the Nairobi urban poor. J Adolesc Health.

[46] Longfield K, Glick A, Waithaka M, Berman J (2004). Relationships between older men and younger women: implications for STIs/HIV in Kenya. Stud Fam Plann.

[47] Maticka-Tyndale E, Gallant M, Brouillard-Coyle C, Holland D, Metcalfe K, Wildish J (2005). The sexual scripts of Kenyan young people and HIV prevention. Cult Health Sex.

[48] Nzioka C (2001). Perspectives of adolescent boys on the risks of unwanted pregnancy and sexually transmitted infections: Kenya. Reprod Health Matters.

[49] Montandon M, Sahin-Hodoglugil NN, Bukusi E, Agot K, Boland B, Cohen CR (2008). Sexuality, HIV risk and potential acceptability of involving adolescent girls in microbicide research in Kisumu, Kenya. Sex Health.

[50] Njue C, Voeten HA, Remes P (2009). Disco funerals: a risk situation for HIV infection among youth in Kisumu, Kenya. AIDS.

[51] Warenius L (2008). Sexual and reproductive health services for young people in Kenya and Zambia.

[52] Papo JK, Bauni EK, Sanders EJ, Brocklehurst P, Jaffe HW (2011). Exploring the condom gap: is supply or demand the limiting factor – condom access and use in an urban and a rural setting in Kilifi district, Kenya. AIDS.

[53] UNAIDS (1998). Social marketing: an effective tool in the global response to HIV/AIDS.

[54] Population Services International (2010). The social marketing evidence base.

[55] Tombros A (2005). Integrating efforts to prevent HIV, other STIs, and pregnancy among teens in developing countries.

# Sub article: The importance of choice in the rollout of ARV-based prevention to user groups in Kenya and South Africa: a qualitative study

**DOI:** 10.7448/IAS.17.3.19157

**Published:** 2014-09-08

**Authors:** Natasha Mack, Emily M Evens, Elizabeth E Tolley, Kate Brelsford, Caroline Mackenzie, Cecilia Milford, Jennifer A Smit, Joshua Kimani

**Affiliations:** 1 Social and Behavioral Health Sciences, FHI 360, Durham, NC, USA; 2 Health Services Research, FHI 360, Durham, NC, USA; 3 Duke Clinical Research Institute, Duke University, Durham, NC, USA; 4 Applied Research Unit, FHI 360, Nairobi, Kenya; 5 MatCH (Maternal, Adolescent and Child Health), Durban, South Africa; 6 Kenya AIDS Control Project, Nairobi, Kenya

**Keywords:** pre-exposure prophylaxis, sub-Saharan Africa, women, ARV-based HIV prevention methods, HIV prevention

## Abstract

**Introduction:**

Stakeholders continue to discuss the appropriateness of antiretroviral-based pre-exposure prophylaxis (PrEP) for HIV prevention among sub-Saharan African and other women. In particular, women need formulations they can adhere to given that effectiveness has been found to correlate with adherence. Evidence from family planning shows that contraceptive use, continuation and adherence may be increased by expanding choices. To explore the potential role of choice in women's use of HIV prevention methods, we conducted a secondary analysis of research with female sex workers (FSWs) and men and women in serodiscordant couples (SDCs) in Kenya, and adolescent and young women in South Africa. Our objective here is to present their interest in and preferences for PrEP formulations – pills, gel and injectable.

**Methods:**

In this qualitative study, in Kenya we conducted three focus groups with FSWs, and three with SDCs. In South Africa, we conducted two focus groups with adolescent girls, and two with young women. All focus groups were audio-recorded, transcribed and translated into English as needed. We structurally and thematically coded transcripts using a codebook and QSR NVivo 9.0; generated code reports; and conducted inductive thematic analysis to identify major trends and themes.

**Results:**

All groups expressed strong interest in PrEP products. In Kenya, FSWs said the products might help them earn more money, because they would feel safer accepting more clients or having sex without condoms for a higher price. SDCs said the products might replace condoms and reanimate couples’ sex lives. Most sex workers and SDCs preferred an injectable because it would last longer, required little intervention and was private. In South Africa, adolescent girls believed it would be possible to obtain the products more privately than condoms. Young women were excited about PrEP but concerned about interactions with alcohol and drug use, which often precede sex. Adolescents did not prefer a particular formulation but noted benefits and limitations of each; young women's preferences also varied.

**Conclusions:**

The circumstances and preferences of sub-Saharan African women are likely to vary within and across groups and to change over time, highlighting the importance of choice in HIV prevention methods.

## Background

Sub-Saharan African women at risk of HIV infection are a heterogeneous group. They include married and unmarried women, women whose partner(s) may have other partners, women in serodiscordant couples (SDCs), women in polygamous relationships, adolescent girls and women who engage in a range of transactional sexual behaviours with one or more partners. Given the diverse characteristics of African women, their needs and preferences for HIV prevention methods are likely to differ.

Stakeholders in HIV prevention continue to discuss the appropriateness of different formulations of antiretrovirals (ARVs) as pre-exposure prophylaxis (PrEP) for HIV prevention among women in sub-Saharan Africa and elsewhere [1]. In particular, these women and others at risk of HIV need formulations they can adhere to given that effectiveness correlates with adherence [2]. This was demonstrated in the iPrex study, which found high effectiveness of oral PrEP among participants with high adherence [3], as well as in the FEM-PrEP and MTN 003 trials, both of which were stopped for futility and later reported low adherence to oral PrEP among participants [4–6]. The CAPRISA 004 trial of 1% tenofovir gel also found that the product's effectiveness corresponded with degree of adherence [7].

Evidence from family planning research provides an insight into how to encourage uptake of and adherence to prevention methods, thereby increasing overall coverage at the population level. Specifically, contraceptive use, continuation and sustained adherence may be increased by expanding the method mix, improving features of methods and increasing the quality of and access to family planning services [8]. Providers’ assistance with the selection and continued use of a method has also been found to be essential to sustained contraceptive use. Another important lesson is the key role of choice in uptake of and adherence to contraception – choice in terms of the user's ability to select a method, a range of contraceptive methods to choose from and places to access them [8–10].

Extrapolating from family planning, to explore the potential role of choice in women's use of HIV prevention methods, we conducted a secondary analysis of data from qualitative research with potential user groups of ARV-based PrEP formulations in Kenya (female sex workers [FSWs], men and women in SDCs) and in South Africa (adolescent and young women). Our objective here is to present our analysis of these groups’ attitudes towards ARV-based HIV prevention, including acceptability, interest and concerns, and their preferences for different formulations – choosing from pills, gel and an injectable.

## Methods

This study was conducted in Kenya and South Africa from 2011 to 2012 in collaboration with local partners. This was subsequent to the 2010 announcement of the positive efficacy results of the CAPRISA 004 clinical trial on 1% tenofovir gel [7] and the iPrex oral PrEP trial [3], but prior to the early closure of the FEM-PrEP oral PrEP trial and the MTN 003 results, which found that oral PrEP (FEM-PrEP and MTN 003) and gel (MTN 003) were not effective because women did not adhere to the products [4–6]. In Kenya, the research was managed by the FHI 360 Kenya office in collaboration with the University of Manitoba/University of Nairobi Sex Work Outreach Program (SWOP), with data collected in Nairobi and Nakuru. In South Africa, data were collected in the eThekwini District of KwaZulu-Natal by MatCH (Maternal, Adolescent and Child Health; Department of Obstetrics and Gynecology; University of Witwatersrand).

Potential participants for focus group discussions (FGDs) with FSWs were identified through the SWOP clinics, a group of Nairobi, Kenya, clinics serving FSWs. Clinic staff informed clinic clients of the study objectives and participant selection criteria. Clients then voluntarily chose to participate. All participants were age 18 or older, residents of Nairobi and self-identified FSWs. To recruit SDCs, staff working with an SDC support group at the local public hospital in Nakuru, Kenya, discussed the study with potential participants who then voluntarily participated. All participants were age 18 or older, residents of Nakuru and self-identified as being in a sexual relationship with someone of the opposite HIV status. Participants for FGDs with adolescent and young women were recruited with the help of a pre-existing community advisory board (CAB) that was affiliated with the local South African research group who conducted the data collection activities. The CAB conducted community meetings to share information about the research with potential participants and the community at large. Participants for the FGDs with adolescent women were age 14 to 17; those in FGDs with young women were 18 to 24. After obtaining parental permission for potential participants under the age of 18, adolescents and young women expressing interest in the study were recruited by phone.

The risk profile of potential participants was the key determinant for recruitment. Aside from age (for adolescent and young women) and partnership status (for SDCs), other demographic factors were not examined.

The study protocol was approved in the United States by FHI 360's Protection of Human Subjects Committee; in Kenya by the Kenya Medical Research Institute; and in South Africa by the University of the Witwatersrand Human Research Ethics Committee and the eThekweni District and KwaZulu-Natal Provincial Departments of Health.

Written, informed consent was obtained from all participants age 18 and above. For participants age 14 to 17, informed consent was obtained from their parents or caregivers, and informed assent was obtained from the participants.

### Data collection

In Kenya, we conducted six FGDs – three in Nairobi with a total of 33 FSWs aged 21 to 37, and three in Nakuru with SDCs aged between 24 and 48 with a total of 32 participants (16 men, 16 women). In South Africa, we conducted four FGDs with a total of 36 participants. Two were with adolescent girls age 14 to 17, and two were with young women age 18 to 24. One FGD in each user group was with participants from informal settlements, and one with participants from formal settlements; this was a proxy for socioeconomic status, as residents of informal settlements tend to have a lower socioeconomic status than those of formal settlements. In the FGDs, we explored the potential user groups’ interest in and preferences for three different formulations of PrEP – oral PrEP, a vaginal gel and an injectable. The FGD guides comprised a combination of standard and population-specific questions.

Prior to the FGDs, all study participants were given information on PrEP products, including how HIV-negative individuals could use PrEP to avoid becoming infected with HIV, that PrEP products contain ARV-based drugs similar to the ones used to treat HIV-positive individuals, that PrEP products are still being researched, descriptions of the different formulations of PrEP and how they would be used, and how PrEP would likely offer partial protection from HIV infection.

Each FGD was conducted by two research assistants trained in research ethics and qualitative research methods, and fluent in English and the local language (isiZulu in South Africa, Kiswahili in Kenya). One research assistant served as the moderator and the other as the note taker. All FGDs were audio-recorded and simultaneously transcribed and translated into English as needed.

### Data analysis

Through a standard iterative process, we developed a codebook to structurally and thematically code the FGDs using QSR NVivo 9.0. Initially, two data analysts independently coded a sub-set of transcripts, and inter-coder agreement was assessed. This process was repeated until inter-coder agreement was reached for each set. Subsequently, each transcript was coded once by a given analyst, with the analysts consulting one another frequently to ensure that coding decisions were made jointly. The codebook was updated as new themes emerged. Once all data were coded, we generated code reports and conducted inductive thematic analysis to identify major trends and themes.

## Results

### Attitudes towards PrEP, including acceptability, interest and concerns

#### FSWs, Kenya

FSWs in Kenya expressed great interest in PrEP products. However, the majority appeared to assume initially that PrEP would provide 100% protection against HIV, and many were disappointed to learn that they would still need to use condoms to prevent HIV and other sexually transmitted infections (STIs). The majority of FSWs’ concerns regarding PrEP were related to its degree of effectiveness and to their preference for a PrEP product that could prevent both HIV and other STIs. They feared that some FSWs (and other users) would forgo condoms if PrEP became available, thus increasing the likelihood of transmitting HIV and STIs.

FSWs’ interest in PrEP was not only related to preventing HIV transmission, but also to helping them earn more money – because they would feel safer accepting more clients or would feel more comfortable having sex without using condoms. As one FSW described:

They [FSWs] will not have unprotected sex carelessly because as we have heard from some of us earlier on; they said that they usually have unprotected sex when they are offered more money like 3000 [Kenyan Shillings, about $35 US dollars] for sex without a condom or 300 [Kenyan Shillings, about $3.50 US dollars] for sex with a condom. PrEP will help in protecting them from getting HIV while making more money.

A few FSWs expressed concern about side effects, either generally or in relation to particular formulations. Most of these participants emphasized the need to help each woman identify the formulation appropriate for her.

#### SDCs, Kenya

SDCs in all FGDs expressed enthusiastic support for PrEP. We did not systematically survey the SDCs on whether they were currently using condoms consistently, although some mentioned that they were using condoms. However, the overwhelming consensus was that PrEP could, should, or will replace – rather than accompany – condoms as the primary form of HIV prevention within SDCs. Many respondents said they would not want to use PrEP and condoms at the same time.

SDCs also expressed great optimism and hope that PrEP would help reanimate sex lives and draw couples closer together by reducing or eliminating the need for condoms, which many participants described as diminishing sexual pleasure: *You see, we forgot having unprotected sex, so our love for each other will be revived* [with PrEP]. Another respondent explained, *We will accept them* [PrEP products] *as will clear the thoughts of using condoms and hence improve the relationship with my wife*. Participants frequently mentioned PrEP as a way to alleviate the stress associated with condom breaks.

Few SDCs had concerns regarding PrEP, but those that did worried about its safety or about the burden of daily-use formulations to the user.

#### Adolescent girls, South Africa

Adolescents were also interested in PrEP as protection from HIV, although a few participants appeared confused about whether PrEP would protect them from pregnancy as well. They found PrEP particularly appealing because it would eliminate concerns about being seen while obtaining condoms from clinics and because they felt PrEP products could be used privately. As one adolescent explained, *PrEP products … are much easier to use because you can just take a pill, unlike condoms because you are scared to get them from the clinics because you do not know who is watching you*. Some girls expressed hesitation in regards to PrEP, stating that they would be interested in using it only after seeing other girls use it.

#### Young women, South Africa

Young women were excited about and interested in using PrEP products to prevent HIV infection. One concern mentioned by several young women was that it might be difficult for women to use coitally dependent PrEP formulations while under the commonly reported influence of alcohol or drugs prior to sexual activity. Another concern was that it would be challenging to negotiate PrEP use with a partner or that young women simply would not use the product with trusted partners, similar to condom use patterns. One young woman explained that if a male partner *says he does not want to use* [PrEP] *today, you will not use it*.

Unclear on the correct timing of the use of different formations of PrEP, some young women were concerned that PrEP might be difficult to use in the midst of a sexual encounter: *Once your hormones are high, you forget […] both your hormones are already high. When are you going to insert the gel? When are you going to take the pills once you are in the middle of it?* A few women thought their peers would be more willing to use PrEP if they could use it covertly. No differences were identified between adolescent and young women from informal and formal settlements.

### Preferences for PrEP formulations

#### FSWs, Kenya

The majority of FSWs clearly preferred an injectable over other formulations. They liked that one dose would last for a prolonged period of time and would require little user intervention, unlike a pill that they must remember to take every day. In addition, injections were perceived as relatively private, making it less likely that others would know a woman was using the product. A few women described alcohol use as potentially interfering with their ability to take a daily pill but not posing a problem with injections, as this respondent explained:

*Ah okay, I would prefer the injection because it is private and you can use it without your client or partner knowing. Just like the family planning, injection most women who are using it, their partners are not aware. Secondly I personally love taking alcohol so with the PrEP injection I will be safe because I won't need to remember to take it daily like the oral pill or vaginal gel*.

Most FSWs were concerned that they might forget or that it would be burdensome to take a pill consistently. Several women were concerned about side effects associated with an oral product or interactions between pills and alcohol. A few women were worried that people would see them taking pills, whereas others liked the idea of taking a pill because it was daily, something they controlled and similar to contraceptive pills.

Nearly all FSWs reported using some form of vaginal lubricant in the past; however, there was little interest in PrEP gel. This was because women thought they might forget to apply it before and/or after sex and because clients might be against its use. As one FSW noted, *If asking a client to wear a condom is challenging then you can only imagine what they will do if they see you applying the vaginal gel before sex. It is crazy*.

Most FSWs said they would prefer to apply a gel before sex rather than afterwards; however, they saw problems with applying the gel at any point. For example, although the gel might help with lubrication before sex, some FSW noted that there would not always be time to apply it before sex and that FSWs who are drunk or high might forget to use it. Those who cited specific limitations with post-coital use most often noted that women were tired after sex and unlikely to remember to use it.

#### SDCs, Kenya

The majority of SDCs strongly preferred injectable PrEP because it required little user involvement and was long-lasting, in contrast to pills or gel. For example, one person said:

I also believe that the injection will be ideal because one won't be getting a jab every day, maybe it will be three or six months instead of every time you want to have sex, you either take the pills or apply the gel.

Another respondent said:

If it will be for three months I will know it is after every three months, it is better than the pills as one can forget to swallow, or maybe you did not have the pills with you because you travelled, so one will be at high risk.

Others said an injectable would be preferable because they did not like swallowing pills or taking medicine. Some individuals, however, said they would not use an injectable because they feared needles or the pain associated with injections.

Few individuals preferred pills to the other formulations; reasons for preferring pills included: liking the idea of an intermittent pill taken only when having intercourse, being accustomed to taking pills and disliking injections. Disadvantages of pills included a dislike of pills, having to remember to take them regularly and needing to remember to take them along when leaving the house or traveling.

Only three individuals commented on the gel formulation, but these three individuals thought it would be good because it would not be painful like an injection and would help men learn about the vagina. Fewer than half of the females in the SDCs in our focus groups reported ever using a vaginal lubricant or medicine. Among those who had used a vaginal product, the majority had used a vaginal medicine during pregnancy. SDCs overwhelmingly agreed that a PrEP gel should be applied before sexual intercourse, as this respondent commented: *I was feeling that the gel should be applied way before even starting to romance because I believe that it will be ready to fight with the virus*. Couples insisted that a gel provided before sexual intercourse would be more effective than one provided afterward. Couples noted that condoms are used before, not after sex, and they compared pre-coital use to “prevention” and post-coital use to a “cure.”

#### Adolescent women, South Africa

Adolescents did not express a clear preference for a particular PrEP formulation but noted some benefits and limitations of each one. Participants noted that users could forget to take all PrEP formulations. Adolescents felt that the process of obtaining pills could be more private than obtaining condoms, since pills in general might be taken for a variety of reasons. Additionally, whereas PrEP injections would require users to wait in a clinic and a gel would require privacy to insert, pills could be taken relatively privately at any time and place. Additionally, one adolescent noted that it would be easier to take a pill every day than try to predict when sex might happen. Some disadvantages associated with PrEP pills included difficulty swallowing them and concerns that they might cause urine to smell bad.

Adolescents’ concerns about injections included their potential to be painful, with some preferring an injection in the arm rather than the buttocks because they perceived it as less painful. A few adolescents also believed walking or sitting would be difficult after receiving an injection to the buttocks. Several girls were uncomfortable with having to remove their pants or skirts in order to receive the injection.

Only a few (four) adolescents commented on gel dosing. Half felt that it was better to use a gel daily in order to establish a routine. They also noted that daily use would limit situations in which girls wanted to have sex but did not have the gel with them. The other half thought a gel formulation would be better if used intermittently, either pre or post-coitally, especially for adolescents who did not frequently engage in sexual relations.

Participants debated over whether gel or pills were preferable. Advantages of gel included the perception that coitally dependent use was less burdensome than a daily pill and less painful than an injection. Disadvantages included the belief that predicting sex could be difficult, inserting a vaginal gel was unpleasant and required privacy, and a gel could have an unpleasant odour.

#### Young women, South Africa

Some young women preferred PrEP pills compared to other formulations because they believed pills were safer than injections and more private than gel. However, others felt it would be difficult to take a pill every day. Some young women preferred a coitally-dependent gel over pills because they thought it would be easier to remember to apply a gel. Still others preferred injections over a pill because they thought injections would be safer, longer lasting, more private and difficult to forget. One woman expressed concern that injections might not be effective in combination with alcohol and drugs.

Compared to adolescents, young women expressed less concern over receiving an injection in the buttocks. Some young women actually preferred that location, explaining that they were already accustomed to receiving contraceptive injections there. No differences were identified between adolescent and young women from informal and formal settlements.

## Discussion

This study contributes to existing literature on the acceptability of ARV-based PrEP formulations among beneficiary groups. All four potential target populations expressed strong interest in these products, similar to the favourable reactions identified in previous research among other key groups [7, 11–21]. Furthermore, our research introduces the concept of choice into work on the acceptability of HIV prevention methods. Although the importance of choice has been recognized and incorporated into contraceptive provision, it is an emerging idea in HIV prevention, most likely because condoms have been the only method of protection thus far. A range of new potential HIV prevention methods is in development, but there continues to be tension about which to develop and move forward – and our research suggests that no one method will meet all needs.

Although the SDCs in our study preferred the injectable formulation, their rationale for their support of the method is consistent with SDC participants of the Partners PrEP trial of oral tenofovir-only or oral Truvada (tenofovir combined with emtricitibine) in Uganda; SDCs from both studies viewed use of a PrEP product as a “way out” of the “discordance dilemma,” as defined in the Partners PrEP study, that is, that long-term condom use is unrealistic for SDCs (i.e. expensive, inconvenient, uncomfortable and incompatible with the desire to become pregnant), with preserving health and remaining in the relationship seemingly incompatible [19].

Although most women in the CAPRISA 004 trial testing 1% tenofovir gel found it acceptable [7], in our study, hypothetical use of a PrEP gel had mixed acceptability. In particular, some SDCs, FSWs and adolescent women found it unacceptable due to the inconvenience of pre- and post-coital application. In addition, FSWs cited clients’ potential disapproval of the gel as a disadvantage, suggesting that they may not seek or be able to use it covertly. In contrast, African, Indian and Peruvian FSWs have been found to support covert use of gels, viewing this as an advantage of the formulation [13, 22]. It has been suggested that for young adult women, men's passive acceptance of microbicide gel use may be more important than covert use [23]. Notably, adolescent participants in our study did not mention disclosure to partners as playing a role in their interest in PrEP gel or other PrEP formulations. However, we did not ask them if they were sexually active, and those who were not would not have had experience communicating with partners about HIV prevention or considering issues of disclosure.

This study provides some initial information on the preferences of at-risk populations for different formulations of PrEP, a topic about which little is yet known. In our study, most (but not all) FSWs and SDCs in Kenya preferred an injectable PrEP formulation, which they viewed as long-lasting and private. There was greater variation among adolescent and young women in South Africa regarding their preferences for PrEP formulation, with each group noting some benefits and limitations associated with each one. Factors affecting method preferences among the target groups included privacy of the method (for all but SDCs). Adolescent and young women especially were concerned with how PrEP products could be used privately; in particular inserting gels was seen as difficult to do privately while, conversely, pills were viewed as a product that could be used without others such as partners, friends, or family members noting or asking questions. Concerns over privacy are likely linked to an underlying stigma of adolescent and young women having sex, an issue that must be addressed to stimulate demand for PrEP products among this group.

Other factors that affected method preferences included: frequency of use, painfulness, the need for user intervention, side effects, interaction with alcohol and drugs, coital dependence, partner approval and convenience. Interestingly, efficacy was not mentioned as a factor influencing formulation preference, although it was mentioned in reference to the acceptability of formulations; however, it is possible that participants assumed all methods would be equally effective.

Offering PrEP users a choice of methods could also provide an additional benefit – the process of educating users on the methods available could serve as an opportunity to provide individualized risk-reduction counselling. PrEP trials routinely offer this and see benefits associated with the provision of this information. Additionally, method distribution and the regular HIV testing required for PrEP provision is another opportunity for the provision of brief counselling.

Among the limitations of our study are the small number of FGDs, the use of convenience sampling which may not have produced a fully representative sample of the target populations and questions about hypothetical use of these PrEP products, which may differ from both clinical trial use and real-world usage of available products. We also did not systematically collect information on whether condom migration would occur with the introduction of PrEP formulations, although some groups brought this up during the discussion. An additional limitation is that the context-specific nature of the results may limit generalizability of the findings to other cultures and country settings, but this ultimately relates to our main observation and conclusion.

## Conclusions

Women at risk of HIV in sub-Saharan Africa are a diverse population and do not necessarily share the same life circumstances, social norms, risk profiles or perceived ability to use particular PrEP formulations. Their circumstances and needs, as well as choices, are also likely to change over time as their lives take course, depending, for example, on age, relationship status and parity. Sub-Saharan African women's preferences for HIV prevention methods and formulations may be similar within particular user groups – or not, highlighting the importance of ensuring the availability of choice in HIV prevention methods.

## References

[1] Person AK, Hicks CB (2012). Pre-exposure prophylaxis – one more tool for HIV prevention. Curr HIV Res.

[2] Baeten J, Celum C (2012). Oral antiretroviral chemoprophylaxis: current status. Curr Opin HIV AIDS.

[3] Grant RM, Lama JR, Anderson PL, McMahan V, Liu AY, Vargas L (2010). Preexposure chemoprophylaxis for HIV prevention in men who have sex with men. N Engl J Med.

[4] Van Damme L, Corneli A, Ahmed K, Agot K, Lombaard J, Kapiga S (2012). Preexposure prophylaxis for HIV infection among African women. N Engl J Med.

[5] van der Straten A, van Damme L, Haberer JE, Bangsberg DR (2012). How well does PREP work? Unraveling the divergent results of PrEP trials for HIV prevention. AIDS.

[6] Marrazzo JM, Ramjee G, Nair G, Palanee T, Mkhize B, Nakabiito C Pre-exposure prophylaxis for HIV in women: daily oral tenofovir, oral tenofovir/emtricitabine or vaginal tenofovir gel in the VOICE study (MTN 003).

[7] Abdool Karim Q, Abdool Karim SS, Frohlich JA, Grobler AC, Baxter C, Mansoor LE (2010). Effectiveness and safety of tenofovir gel, an antiretroviral microbicide, for the prevention of HIV infection in women. Science.

[8] Ross J, Stover J (2013). Use of modern contraception increases when more methods become available: analysis of evidence from 1982–2009. Glob Health Sci Pract.

[9] Frost J, Darroch J (2008). Factors associated with contraceptive choice and inconsistent method use, United States, 2004. Perspect Sex Reprod Health.

[10] Gray A, Smit J, Manzini N, Beksinska M Systematic review of contraceptive medicines. “Does choice make a difference?”.

[11] Nodin N, Carballo-Dieguez A, Ventuneac AM, Balan IC, Remien R (2008). Knowledge and acceptability of alternative HIV prevention bio-medical products among MSM who bareback. AIDS Care.

[12] Heffron R, Ngure K, Mugo N, Celum C, Kurth A, Curran K (2012). Willingness of Kenyan HIV-1 serodiscordant couples to use antiretroviral based HIV-1 prevention strategies. J Acquir Immune Defic Syndr.

[13] Galea JT, Kinsler JJ, Salazar X, Lee SJ, Giron M, Sayles JN (2011). Acceptability of pre-exposure prophylaxis as an HIV prevention strategy: barriers and facilitators to pre-exposure prophylaxis uptake among at-risk Peruvian populations. Int J STD AIDS.

[14] Liu AY, Kittredge PV, Vittinghoff E, Raymond HF, Ahrens K, Matheson T (2008). Limited knowledge and use of HIV post- and pre-exposure prophylaxis among gay and bisexual men. J Acquir Immune Defic Syndr.

[15] Mimiaga MJ, Case P, Johnson CV, Safren SA, Mayer KH (2009). Preexposure antiretroviral prophylaxis attitudes in high-risk Boston area men who report having sex with men: limited knowledge and experience but potential for increased utilization after education. J Acquir Immune Defic Syndr.

[16] Golub SA, Kowalczyk W, Weinberger CL, Parsons JT (2010). Preexposure prophylaxis and predicted condom use among high-risk men who have sex with men. J Acquir Immune Defic Syndr.

[17] Dunkle K, Wingood G, Camp C, DiClemente R Intention to use pre-exposure prophylaxis among African-American and white women in the United States: results from a national telephone survey.

[18] Van der Elst EM, Mbogua J, Operario D, Mutua G, Kuo C, Mugo P (2013). High acceptability of HIV pre-exposure prophylaxis but challenges in adherence and use: qualitative insights from a phase I trial of intermittent and daily PrEP in at-risk populations in Kenya. AIDS Behav.

[19] Ware NC, Wyatt MA, Haberer JE, Baeten JM, Kintu A, Psaros C (2012). What's love got to do with it? Explaining adherence to oral antiretroviral pre-exposure prophylaxis for HIV-serodiscordant couples. J Acquir Immune Defic Syndr.

[20] Brooks RA, Landovitz RJ, Kaplan RL, Lieber E, Lee SJ, Barkley TW (2012). Sexual risk behaviors and acceptability of HIV pre-exposure prophylaxis among HIV-negative gay and bisexual men in serodiscordant relationships: a mixed methods study. AIDS Patient Care STDS.

[21] Terris-Prestholt F, Hanson K, MacPhail C, Vickerman P, Rees H, Watts C (2013). How much demand for New HIV prevention technologies can we really expect? Results from a discrete choice experiment in South Africa. PLoS One.

[22] Greene E, Batona G, Hallad J, Johnson S, Neema S, Tolley EE (2010). Acceptability and adherence of a candidate microbicide gel among high-risk women in Africa and India. Cult Health Sex.

[23] Rupp RE, Rosenthal SL (2003). Vaginal microbicides and teenagers. Curr Opin Obstet Gynecol.

# Sub article: Community and research staff collaboration for development of materials to inform microbicide study participants in Africa

**DOI:** 10.7448/IAS.17.3.19156

**Published:** 2014-09-08

**Authors:** Cynthia Woodsong, John Michael Mutsambi, Smangalisa Ntshele, Peggy Modikoe

**Affiliations:** 1 International Partnership for Microbicides, Silver Spring, MD, USA; 2 Community Engagement Consultant, Cape Town, South Africa; 3 International Partnership for Microbicides, Paarl, South Africa; 4 TB/HIV Care Association, Cape Town, South Africa

**Keywords:** microbicide trials, community collaboration, participant information materials

## Abstract

**Introduction:**

Clinical trials of new vaginal products require careful communication with participants about trial requirements. Most microbicide trials have been multi-site studies conducted among women in sub-Saharan Africa, where literacy levels and understanding of scientific methods differ from those designing and conducting the trials. Microbicide trials require women to insert objects in their vagina and ensure they are present in the vagina during sex. For many women, this is a novel behaviour. These behaviours take place within the context of clinical trial participation, which is an additional novelty. Research teams must develop informational materials to help participants understand the clinical trial and input from local research staff and community members can improve the content and format of these materials.

**Methods:**

This paper discusses the development of illustrated materials developed for microbicide trial participants, presenting examples from two studies. In both studies, research staff and community advisory groups collaborated to review and revise materials.

**Results:**

Collaborative efforts revealed insights about how to convey information about clinical trial participation and microbicide use. These insights highlighted realities of the local context, details that might be misunderstood, illustrations of a sensitive nature and concerns about blood testing. In particular, information about blood testing and product use instructions required careful consideration. Although the research team anticipated needing advice on how best to convey information on these topics to participants, some aspects of potential participant concerns about these topics were also new to the research team. Community advisors and local research staff suggested better ways to convey this information, and provided guidance on how to use the materials.

**Conclusions:**

The collaboration served to develop informational materials for microbicide trial participants. Furthermore, staff gained a better understanding of issues and concerns that could influence trial participation. A collaborative engagement process can provide important insights into local culture and knowledge beyond what is needed for development of clinical trial participant information materials. Research teams should be sensitive to this possibility, avail themselves of information and take appropriate action.

## Introduction

The continued need for HIV prevention methods that women can use provides motivation for efforts to develop vaginal microbicides, which are products inserted in the vagina and meant to remain present during sexual intercourse to impede sexual transmission of HIV [1]. For many women, this is an entirely novel behaviour, and for those accustomed to traditional vaginal practices [2], the specific use requirements for microbicide products may be more exacting. Use requirements have included timing of insertion in association with sexual intercourse, daily routine insertion of gels and insertion of a vaginal ring that is left in the vagina for weeks at a time [3]. These behaviours take place within the context of placebo-controlled clinical trial participation, which is a further novelty, and careful communication is required to support meaningful informed consent and good adherence to study requirements.

Potential study participants, research staff, community members and the family and social network of participants all need clear information about clinical trials conducted locally [4]. Most microbicide trials have included research sites in multiple countries, and research has been concentrated in sub-Saharan Africa, where incidence of HIV infection is high [5]. In this region, study participants’ literacy and knowledge of scientific methods and concepts often differ from those designing and conducting trials and thus special attention is needed to develop culturally appropriate and effective communication materials about trial procedures and requirements. Incorporation of illustrations in educational materials can improve communication [6, 7], and community input is increasingly used in the development of such materials.

The collaborative process for developing communication materials provides insights into issues that influence clinical trial participation and adherence, as well as those that could impact future introduction and use of products and interventions proven effective. In microbicide research, such collaboration usually occurs through interactions with community advisory groups [8–10]. Communication is intended to flow in both directions, from community to the research group conducting the trial, and vice versa [11].

This paper reviews the collaborative and adaptive processes to develop informational materials for participants in two microbicide trials, one initiated in 2005 and one in 2012, and highlights the accumulated experiences with communication needs over that time. The first trial is HPTN 035, a Phase IIB study of vaginal gels conducted by the HPTN at sites in Malawi, South Africa, United States and Zimbabwe from 2005 to 2009 [12]. The second study, initiated in 2012, is IPM (“The Ring Study”), a Phase III safety and efficacy clinical trial testing a vaginal ring and currently being conducted by the International Partnership for Microbicides (IPM) at research sites in South Africa and Uganda [13].

In the 10 years that passed between initiating design of the HPTN 035 materials and completing materials for participants in IPM 027, much had been learned about the issues and communication needs for microbicide trial participants, and this knowledge has been applied to IPM 027. After providing a summary of the types of informational materials developed for trial participants, we discuss the lessons learned through the collaborative process.

## Discussion

Illustrated materials described here were developed for both studies prior to study initiation, so that they would be available for the initial informed consent process. They were not developed using a scientific research protocol, and they were not formally evaluated or tested. Rather, the materials development process began with drafts prepared by a team working at the study coordinating centre (for HPTN 035) and study sponsor headquarters (for IPM 027). This central team then engaged in a consultative process with staff and community advisory groups at the local research sites.

In HPTN 035, local input on drafts of illustrated materials was obtained from staff at the research sites, including staff responsible for community engagement. At some of the sites, these staff in turn asked for informal feedback from members of their local community advisory group. For IPM 027, input was provided by the staff at the research sites and a more formal process was used to solicit feedback from the community advisory groups associated with the research sites conducting the study. Draft materials were initially presented to research site staff and revised according to feedback. Staff then presented the second draft to their community advisory group, and written feedback was provided to the staff member developing the materials at IPM. Materials continued to be revised until the groups involved considered that feedback had been appropriately incorporated. For both studies, translations followed illustration development, using an iterative process that sometimes required additional adjustments to illustrations. Importantly, the community advisory groups in IPM 027 were given formal feedback on how their recommendations were accommodated in the revised and final versions.

Materials were approved by ethics committees prior to being provided to participants. Centralized training on use of all materials developed for both studies was provided to the research site staff, using a training-of-trainers approach. Training manuals included a page-by-page discussion of the purpose and intent of the illustrations, talking points and counselling approaches to be used with the materials. Training was completed and materials in place prior to the start of both trials.

## Types of materials

The educational materials for participants in both trials use illustrations. In both studies, local input was used to develop the main characters for print materials, which included a study participant, her partner and a staff member at the research site. Since both studies included participants from multi-cultural settings, local staff and community advisors advised that the characters be somewhat neutral, avoiding attire and hairstyles that could be identified with a specific ethnic group. For HPTN 035, separate sets of African and U.S. illustrations were developed, although the content and text (translated into local languages) was the same. These characters appeared throughout the sets of illustrated materials, which included the following items:

1. *Informed consent materials*: Both studies developed table-top flipcharts to be used at the research centres during the informed consent discussion, proving information on study design and purpose, procedures, risks and benefits, confidentiality and what is expected of study participants. In HPTN 035, a booklet version of the flipchart was also given to participants to take with them.
2. *Product use instruction sheets*: Both studies developed illustrated instruction sheets to provide participants with instructions about how to correctly use the study products. Instructions for HPTN 035 focused on how to correctly apply the gel before sex and dispose of the applicator. The instructions for IPM 027 carried forward the illustration concepts for insertion, but expanded the instructions to explain what to do if the vaginal ring is out of the vagina between visits.
3. *Blood testing information sheets*: During the period that HPTN 035 was being conducted, experiences were accumulating in HPTN 035 and other microbicide trials, highlighting a number of issues that are challenging to convey to participants [14–16]. These include aspects of blood testing in general and the HIV testing algorithm adopted for the study. In response to this need, an additional set of information sheets was developed for IPM 027, to be used as needed throughout the trial.

## Adapting to local context

Some of the required revisions were fairly straightforward and would likely have become apparent in a routine review. For example, community advisors noted that not all have access to running water, and thus an illustration of product rinsing was changed to convey a basin of water, rather than a spigot. Similarly, the community advisory group in one country noted that motorcycle transportation is much more common in their area than the minibus portrayed in the original illustration, and thus a motorbike was added to the page. Although both of these example revisions were easily accommodated, it is worth noting that the discussion which highlighted the local context served to further sensitize the research staff across all sites.

Within the local context, some specific items to be illustrated revealed sensitivities of a personal nature. For example, community advisory group members objected to one of the initial depictions of vaginal ring insertion. The instructions show options for inserting a ring either by squatting, lying down or standing with one foot raised. Some community members felt that one of these illustrations could be misconstrued as portraying masturbation. The illustration for IPM 027 was easily modified to depict the ring clearly in the participant's hand. The HPTN 035 informed consent flipchart included an illustration of a woman receiving a pelvic exam, and local advice was to not include this in the booklet version given to women to take home, since it could be embarrassing or confusing if seen by children.

## Clarification of instructions for correct use of microbicides

The product use instructions developed for both studies are undoubtedly stronger because of the local input received. The information is more in tune with the realities of participants’ lives and possible misperceptions have been clarified. The instructions provide a convenient and appropriate visual aid to reinforce product use-adherence. For example, the IPM 027 product use instruction sheet makes it clear that sometimes an expelled ring should not be reinserted, while in other cases it can be rinsed and reinserted. The sheet depicts only ring insertion, not removal, in order to avoid conveying the message that the ring should be removed. The product use instruction sheet used in HPTN 035 clearly illustrated that the applicator should be inserted only halfway into the vagina; this was meant help avoid a mistaken assumption that women should strive to push the applicator in as far as possible, which could possibly cause trauma.

## Addressing blood testing concerns

An important issue that has given rise to rumours and misperceptions in HIV prevention research, including microbicide trials, concerns blood testing [14–16]. In microbicide trials, routine and repeated blood testing is required over extended periods of time (e.g. 1 to 2 years or more), and some scheduled tests may collect multiple vials of blood. The volume and/or frequency of blood testing has given rise to rumours that researchers sell blood for profit-driven or nefarious purposes, including “Satanism.” Some who accept explanations about the need for and purpose of blood testing may nevertheless be concerned about what happens to the blood once tests are complete (e.g. Is it stored? Is it sold? If it is destroyed, how is that done?). On a different note, staff at some research sites noted that participants may not understand how quickly blood is replaced in the body, or how much blood the body contains. Finally, those working in the larger field of HIV/AIDS have long reported that people find HIV testing algorithms confusing, and they may not believe the accuracy of testing results.

On the advice of local staff, the HPTN 035 illustrated materials depicted a hand holding a blood collection vial, to counter previous rumours that cola-sized bottles of blood were collected. For IPM 027 three illustrated sheets were developed to explicitly address the multiple blood draw issues that had been raised in previous trials, and by the community advisors and staff working in the trial sites (Figure 1). One sheet is a simple diagram of the HIV algorithm, using illustrations and characters from the informed consent flipchart to show the sequence of testing when an initial test is positive. A second sheet illustrates the amount of blood drawn at different types of visits (e.g. monthly, quarterly) throughout the trial, using common household items, such as a spoon or measuring cup to convey volume. This sheet is available to supplement the informed consent discussion, as well as at any visit when blood is taken, or the participant expresses concerns or questions. A third illustrated sheet shows how much blood is in the average women's body, again using a household item for comparison, and compares that volume with the amounts taken during the different study visits as well as how much blood a woman normally loses during menses. This sheet also explains that the body replenishes blood within 24 hours, samples are kept in a locked location and that blood is subjected to tests for “HIV, other health problems and dapivirine drug levels.”

**Figure 1:** Supplemental information sheets about blood testing.

Development of all these materials benefitted from community consultation. For example, it was requested that the algorithm sequence, which uses illustrated silhouettes, be accompanied by the flipchart illustration of a blood draw, which had previously been scrutinized and revised to meet community approval. The household items to be used for comparison with various blood volumes were discussed and agreed upon. At the community advisors’ suggestion, an illustration was added to explain that a participant's blood is never mixed with anyone else's. This concern of participants was previously unknown to the research team developing the illustrated materials. The research team was unable to develop a satisfactory illustration of how blood is destroyed (illustrations depicting burning were considered too alarming), so this concern was added to the talking points only.

## Conclusions

The use of illustrations has been shown to be an effective way to convey health information, particularly in low-literacy settings [10]. It is incumbent on research teams to make such materials available to the larger research field, so they can be adapted through a consultative process for use in other settings. Indeed, over the time period covered during the two trials discussed, the basic style for illustrated consent materials and user instructions described in this paper has become widely used in microbicide trials. The blood-testing information sheets are unique, and they provide an example of how research teams with a foundation of illustrated materials can develop additional materials as needed throughout a study, building on participant familiarity with the characters and information format. Although communication about clinical trial objectives, procedures, requirements and risks and benefits is particularly concentrated during the enrolment process, such communication is needed throughout the study period [4].

As an added benefit, the review and consultation process provided the research staff and community advisory groups with a deeper understanding of the trial procedures and requirements, and community concerns and interests. The discussions helped elucidate issues known to be problematic, and surface new ones that required the attention of the research staff. Moving forward, future clinical research could continue to benefit from the accumulated lessons learned through collaborative communication with local research teams and community advisory groups. Knowledge of the experiences of trial participants, the rumours that circulate in trial communities about blood testing and continued engagement with community advisors will be helpful in future research as well as introduction of HIV prevention methods.

## References

[1] Shattock RJ, Rosenberg Z (2012). Microbicides: topical prevention against HIV. Cold Spring Harb Perspect Med.

[2] Martin Hilber A, Hull TH, Preston-Whyte E, Bagnol B, Smit J, Wacharasin C (2010). A cross cultural study of vaginal practices and sexuality: implications for sexual health. Soc Sci Med.

[3] Karim QA, Baxter C, Karim SA (2013). Topical microbicides-what's new?. J Acquir Immune Defic Syndr.

[4] Woodsong C, Karim QA (2005). A model designed to enhance informed consent: experiences from the HIV prevention trials network. Am J Public Health.

[5] UNAIDS (2013). Global report – UNAIDS report on the global AIDS epidemic 2013.

[6] Houts PS, Doak CC, Doak LG, Loscalzo MJ (2006). The role of pictures in improving health communication: a review of research on attention, comprehension, recall, and adherence. Patient Educ Couns.

[7] Peregrin T (2010). Picture this: visual cues enhance health education messages for people with low literacy skills. J Am Diet Assoc.

[8] Strauss R, Sengupta S, Quinn SC, Goeppinger J, Spaulding C, Kegeles SM (2001). The role of community advisory boards: involving communities in the informed consent process. Am J Public Health.

[9] Morin SF, Maiorana A, Koester KA, Sheon NM, Richards TA (2003). Community consultation in HIV prevention research: a study of community advisory boards at 6 research sites. J Acquir Immune Defic Syndr.

[10] Quinn SC (2004). Ethics in public health research: protecting human subjects: the role of community advisory boards. Am J Public Health.

[11] MacQueen K (2014). Stakeholder engagement and good participatory practices for global clinical trials: developing tools and models for the 21st century.

[12] Abdool Karim SS, Richardson BA, Ramjee G, Hoffman IF, Chirenje ZM, Taha T (2011). Safety and effectiveness of BufferGel and 0.5% PRO2000 gel for the prevention of HIV infection in women. AIDS.

[13] IPM (2012). IPM 027 IPM 027.

[14] MacQueen KM, Alleman P (2008). International perspectives on the collection, storage, and testing of human biospecimens in HIV research. IRB.

[15] Stadler J, Eirik S (2010). Rumours about blood and reimbursements in a microbicide gel trial. Afr J AIDS Res.

[16] Fairhead J, Leach M, Small M (2006). Where techno-science meets poverty: medical research and the economy of blood in The Gambia, West Africa. Soc Sci Med.

# Sub article: Learning from the private sector: towards a keener understanding of the end-user for microbicide introduction planning

**DOI:** 10.7448/IAS.17.3.19162

**Published:** 2014-09-08

**Authors:** Amy H Lin, Tiffany L Breger, Matthew Barnhart, Ann Kim, Charlotte Vangsgaard, Emily Harris

**Affiliations:** 1 United States Agency for International Development (USAID), Center for Accelerating Innovation and Impact Washington, DC, USA; 2 Department of Epidemiology, University of North Carolina Gillings School of Global Public Health Chapel Hill, NC, USA; 3 United States Agency for International Development (USAID), Office of HIV/AIDS Washington, DC, USA; 4 IDEO, Boston, MA, USA; 5 ReD Associates New York, NY, USA

**Keywords:** HIV, prevention, microbicides, end-user, user research, women, adherence, introduction planning

## Abstract

**Introduction:**

In planning for the introduction of vaginal microbicides and other new antiretroviral (ARV)-based prevention products for women, an in-depth understanding of potential end-users will be critically important to inform strategies to optimize uptake and long-term adherence. User-centred private sector companies have contributed to the successful launch of many different types of products, employing methods drawn from behavioural and social sciences to shape product designs, marketing messages and communication channels. Examples of how the private sector has adapted and applied these techniques to make decisions around product messaging and targeting may be instructive for adaptation to microbicide introduction.

**Discussion:**

In preparing to introduce a product, user-centred private sector companies employ diverse methods to understand the target population and their lifestyles, values and motivations. ReD Associates’ observational research on user behaviours in the packaged food and diabetes fields illustrates how ‘tag along’ or ‘shadowing’ techniques can identify sources of non-adherence. Another open-ended method is self-documentation, and IDEO's mammography research utilized this to uncover user motivations that extended beyond health. Mapping the user journey is a quantitative approach for outlining critical decision-making stages, and Monitor Inclusive Markets applied this framework to identify toilet design opportunities for the rural poor. Through an iterative process, these various techniques can generate hypotheses on user drop-off points, quantify where drop-off is highest and prioritize areas of further research to uncover usage barriers. Although research constraints exist, these types of user-centred techniques have helped create effective messaging, product positioning and packaging of health products as well as family planning information. These methods can be applied to microbicide acceptability testing outside of clinical trials to design microbicide marketing that enhances product usage.

**Conclusions:**

The introduction of microbicide products presents an ideal opportunity to draw on the insights from user-centred private sector companies’ approaches, which can complement other methods that have been more commonly utilized in microbicide research to date. As microbicides move from clinical trials to real-world implementation, there will be more opportunities to combine a variety of approaches to understand end-users, which can lead to a more effective product launch and ultimately greater impact on preventing HIV infections.

## Introduction

With several ground-breaking possibilities for women-initiated methods of HIV prevention on the horizon, it is critical to begin planning for product introduction. Recent trials of antiretroviral (ARV)-based prevention products have demonstrated that pre-exposure prophylaxis (PrEP) will be effective if adherence is sufficient [1–5]. Though a lack of efficacy was observed in the FEM-PrEP [6] and VOICE (7) trials of PrEP among young women in sub-Saharan Africa, product use was low as measured by drug levels. Thus, hopes remain high that trials of either dapivirine ring (in the Ring Study and ASPIRE) and/or tenofovir gel (in FACTS 001) will confirm the efficacy observed in CAPRISA 004 and ultimately lead to licensure and introduction of one or more new vaginally administered products for HIV prevention.

The importance of integrating behavioural and social sciences in biomedical microbicide trials is widely recognized [6, 8–12], and behavioural science methods have been emphasized in past and on-going HIV research and intervention methodology. While it is beyond the scope of this commentary to provide a comprehensive review of all literature to date, the diversity in the quantitative and qualitative behavioural science methods used to support microbicide planning efforts is noteworthy. Previous and on-going microbicide research has included discrete choice experiments exploring the effect of efficacy and contraceptive potential on a woman's stated willingness to use microbicides [13]; focus groups, interviews and questionnaires examining microbicide acceptability [14–20]; analysis of disclosure of trial participation on microbicide use and adherence [21]; and Motivational Interviewing to identify and address trial participants’ barriers to adherence [22]. However, although behavioural science methods have been used in smaller studies of non-trial participants to examine factors such as perceptions of topical vaginal gels [23, 24] and intravaginal insertion practices [25], studies with this broader community have not been the norm, which has limited data related to potential microbicide end-users. Furthermore, methods from the broader social sciences have not been frequently utilized in HIV research and intervention methodology with either trial or non-trial participants.

As microbicide product development moves from clinical trials to real-world implementation in the coming years, there will be more opportunities to use complementary methods to understand women's interaction with a real product in a less artificial context. Deepening awareness of the barriers to microbicide use and access in a more demographically diverse population will be essential to ensuring this prevention method meets its potential to empower women in their sexual relationships and health. In this regard, ‘user-centred’ private sector companies (distinguished from marketing firms by a more intensive and open-ended approach to understand the end-user) have demonstrated success in increasing uptake of many different types of products. This has been achieved through utilizing a wide range of techniques to understand the target population as well as their lifestyles and broader environment [23, 26–33]. While behavioural and social sciences provide the foundation for this approach, examples of how the private sector has applied these techniques to make decisions around product messaging and targeting for effective product introduction can be instructive for the public sector's adaptation to the microbicide arena, particularly in the post-licensure phase of microbicide research.

## Discussion

To understand the underlying themes relevant to a new product, user-centred private sector design and marketing firms typically begin by speaking to and studying a wide range of individuals – not only potential end-users outside of the clinical trial context but also other individuals in the environment who may have widely disparate views [28, 32]. Speaking to ‘extreme’ users (e.g. those who embrace abstinence, as well as those who prefer sex without any protection) uncovers attitudes and beliefs that inform the understanding of the target user [32]. While prevention of HIV is the main objective for the public health world, for the user, that may not be the only mental framework. In the case of microbicides, perceptions of the product and HIV, as well as larger issues like beauty, wellness and social norms must be considered for a successful rollout.

Moreover, to fully understand user experiences, perceptions and underlying motivations, the private sector focuses on the broader context affecting decisions that are made around product use, investing heavily in understanding the larger ecosystem of values, beliefs, lifestyles, habits and other influencers of behaviour [27, 28, 33]. While methods used in microbicide trials such as in-depth interviews, focus groups and surveys are important for understanding end-users, one potential limitation is that they can produce an artificial situation where respondents answer according to what they believe is expected and may not reveal what they actually think or do [34]. The flexibility and open-ended nature of in-context observation can potentially mitigate this social desirability bias since this approach does not rely entirely on the interviewee's accountability and interest in articulating underlying motivations [33]. Additionally, it can help generate new hypotheses since questions are not pre-determined and will organically arise from real-time observations [26, 28].

### In-depth observational methods to understand end-users

One method of understanding a user's context and lifestyle is to follow users through their habits and routines, referred to as ‘tag along’ or ‘shadowing’ [28]. This technique can be used to complement in-depth interviews by revealing disconnects between individuals’ aspirations versus actions. ReD Associates demonstrated this in their work in packaged food (ReD Associates is an innovation and strategy consultancy that employs the methods of social science and market analysis). When interviewing mothers about how they choose between different types of food, researchers heard an emphasis on nutrition, organic ingredients and other aspects that emphasized health but found packaged foods that were primarily convenient, fast and easy to use upon looking in the refrigerator. Because similar discrepancies have been recognized between self-reported and measured microbicide adherence [6], these observational techniques might provide an additional source of information from lifestyles or emotional cues to triangulate and better interpret self-reported data.

Furthermore, these techniques have been useful in revealing the role of stigma and other root causes of behaviours. In the diabetes field, ReD Associates’ observational research found that patients would skip shots when in public due to a sense of self-consciousness or a fear of stigma from being identified as diabetic, contradicting the industry's prior assumption that lack of consistency was due to forgetfulness. This insight emphasized the need for discretion – for example, through less intrusive product designs with syringes that looked more like pens – over an intervention based on setting up timed or automated reminders.

While it is understood generally that HIV-related stigma affects microbicide use, our understanding of more specific facilitators of, and barriers to, microbicide use might be enhanced by direct observational research. Though it would not be possible for a researcher to tag along in every situation, and obviously not during sexual intercourse, a researcher may still gain valuable insight by joining a woman as she starts her weekend to observe her social interactions, the physical environment of her home and the places she goes, and even what she carries in her handbag to glean what she associates with sex. Post-observation, the researcher can follow-up with women to determine what sexual health choices were made. This can provide insight into aspects of daily life routines that influence usage of and adherence to microbicides. For example, women might associate cosmetics with beauty and microbicides with medication, which could result in bringing cosmetics – but not a microbicide – to a bar or to meet a sexual partner. This type of observation and usage driver can inform microbicide design so that packaging and marketing invoke the branding of beauty products instead of medications.

Another observational technique commonly used by the user-centred private sector is self-documentation, or enabling users to create photo and video diaries [32]. IDEO recently engaged in a project to uncover women's attitudes and experiences around mammography in the United States (IDEO is an international design and consultancy firm). Rather than accompanying each woman to individual mammogram appointments, the women were given video cameras and recorded their reactions and thoughts before, during and after the mammogram appointment. Giving women the freedom to record in the intimacy of their homes, doctor's offices and other relevant settings enabled open and comfortable reflection, revealing that some women were sceptical of mammograms, while others experienced mammograms as life-saving. Participation in the exercise over time highlighted how reasons for adherence to mammograms extended beyond cancer detection to larger themes around femininity, wellness, self-confidence and modelling behaviour for daughters.

Given the private nature of microbicide use, application of this technique can increase users’ sense of agency in providing feedback. The approach addresses practical needs (since it is not feasible for researchers to ‘embed’ themselves in women's sexual lives), while giving users flexibility and room for privacy. Mobile phones with photo and video capability are becoming more common in developing country settings and might be viewed by microbicide users as an interface that is more approachable than options such as writing essays or completing written questionnaires – and less subject to social desirability bias than in-person interviews. Although this might not be possible in all contexts due to privacy or institutional review board concerns, technology is becoming further integrated into research practice, and similar methods such as PhotoVoice, are also popular in advocacy efforts and women's health [35, 36]. Photo and video journaling can provide insight into the subject of microbicides in users’ minds, the connection to themes beyond the direct purpose of HIV prevention and the interlinked associations that may influence product uptake and usage.

### User journey mapping

Mapping the user journey is another user-centred private sector approach that goes hand-in-hand with observational research [37] and can be employed during the post-licensure phase when microbicides are introduced. This quantitative approach provides a framework for outlining the critical decision-making stages a potential user passes through in order to become a regular product user, allowing researchers to better understand potential drop-off points and calculate attrition rates for each stage. Illustrating this structure is part of an iterative process of end-user research in which initial observational research can generate hypotheses regarding potential drop-off points, and user journey mapping can calculate where drop-off is highest. This can then inform areas where more in-depth observational research can most efficiently and effectively hone in on root causes for these drop-offs.

Monitor Inclusive Markets (MIM) created this type of decision-making map through its customer research in Bihar, India, around willingness of the rural poor to purchase toilets (MIM is a unit of the Monitor group that focuses on market-based solutions for social change). Mapped against the user journey, this research revealed that financial constraints were substantial barriers at the purchase stage, and residents who may otherwise have bought a toilet found it unaffordable. Upon further investigation of this stage, researchers discovered that potential users wanted a long-term sanitation solution and assumed that requiring a 10-foot deep waste pit posed a significant cost. Armed with this knowledge, researchers were able to suggest an alternative, long-term toilet design with lower upfront cost, by using two five-foot deep pits [38]. The user journey analysis also revealed that key influencers of target users were friends, community members and local masons, suggesting the importance of building support for this new design among these trusted information sources.

Examining the cascade of care is not a foreign concept for researchers in the HIV field. What is perhaps different in emphasis amongst user-centred private sector firms is the degree of rigour in focusing on the journey from the end-user's perspective rather than the clinical and health system utilization components of a supply-side oriented perspective. By focusing on the end-user experience with microbicides over time, the user journey framework can enable stages to be identified; examined for important barriers, drivers and influencers of microbicide use; and prioritized for further study to ultimately understand and address root causes of low uptake and adherence. Figure 1 illustrates how this iterative user journey mapping process might be used for microbicide users as well as the information it might provide.

**Figure 1:** User journey mapping for microbicide user illustrating opportunities to identify potential drop-off points and barriers, drivers and influencers at each stage.

### Applying user research to product messaging, positioning and packaging

Synthesis of user research and resulting insights may open possibilities beyond those provided through biomedical trials, behavioural sciences and similar work with a greater focus on the public health interests of disease prevention and drug properties. Bedsider.org, a campaign designed by IDEO with the National Campaign to Prevent Teen and Unwanted Pregnancies, addresses unplanned pregnancy among young women. Through stakeholder research, Bedsider focused on five areas for behavioural change, including awareness and motivational drivers. By offering medically accurate information through a sex-positive brand and addressing a stigmatized topic with humour (versus a clinical, prescriptive tone) and various kinds of media (video, testimonials and other interactions), Bedsider's communication is more engaging and effective for the target audience of women aged 18–29, among whom 7 out of 10 pregnancies are unplanned. To date, over one million users have already visited Bedsider.

While in some cases ‘demedicalizing’ a product may be important, highlighting a product's medical nature can also increase uptake. A psoriasis treatment manufacturer thought potential users were anxious about side effects and marketed their treatment as a beauty product, or ‘just like a cream’. User research conducted by ReD Associates revealed that patients thought it wasn't working and stopped using it. The manufacturer changed the packaging to emphasize potency, and usage increased. With microbicides, user research should similarly play an important role in informing messaging, positioning and packaging.

### Constraints and challenges

In addition to the illustrative techniques outlined above, many other non-traditional methods to understand end-users can be explored for microbicide introduction planning, including co-designing, storyboarding, role plays and drawing as response. Whichever methods are chosen, the following constraints and challenges may arise.

One key issue is how to best test acceptability of a new product when it does not yet exist. Though acceptability assessments are incorporated from phase I to phase III clinical trials, it is critical to gain a more comprehensive understanding of acceptability among the broader (i.e. non-clinical trial participant) population, especially before a completely new class of agents is introduced. One potential workaround is developing an early prototype during the pre-licensure phase and observing how a more diverse population of potential users interacts with this mock product. For example, IDEO developed an insulin starter kit that users could see and touch, enabling researchers to understand how injection instructions were best integrated into the kit, which visuals and text resonated emotionally, and where the kit would be stored at home. By enabling users to physically engage with a prototype and role-play, researchers could better understand how users view the product and how it would fit into users’ lives [28, 30].

Prior to microbicide licensure, lubricants or contraceptive vaginal rings that are already on the market may provide opportunities to conduct user-focused research with products that have commonalities with microbicides. Although motivations for using HIV prevention products are closely linked to risk perception, there are likely to be additional marketing cues that can encourage use, such as product packaging (e.g. colour choice, images, similarities to other product packaging), complexity of instructions or inserts (e.g. terminology, written versus visual directions) and other factors. Conducting this type of acceptability assessment with a broader population can inform the design that is most likely to maximize use and adherence among target demographics.

Another important aspect for user research is to recognize the product promoter's (or promoters’) constraints. These can be investment ceilings, insufficient human resources, or strict regulatory requirements that restrict the ability to act on user insights. It is important to take stock of these constraints early on and to conduct user research with an eye towards producing practical, actionable recommendations. In the case of microbicides, late-stage products using an ARV as the active pharmaceutical ingredient are unlikely to be available for sale over-the-counter, and thus could not be physically placed on the same shelf as other female-focused products like cosmetics, sanitary pads or lubricants. However, early product design prototypes might include packaging that combines condoms and microbicides, emphasizes beauty, promotes sexual pleasure advantages and/or is described by health care service providers in the context of broader issues of women's health and family planning. Instead of messaging only HIV prevention, these approaches might inspire desire for a product, modelled after consumer behaviours (versus patient behaviours).

Lastly, ethical considerations may constrain end-user research that can be done with regard to microbicides in comparison to common consumer products. The examples highlighted in this commentary – for example, contraceptives, diabetes medication and mammography – illustrate that these methods can be successfully and ethically applied to sensitive medical products.

## Conclusions

While biomedical HIV trials and sexual health research utilizes multidisciplinary methods to encourage uptake and adherence in introducing new products, additional user-centred private sector techniques can be integrated outside of the clinical trial context to enhance microbicide introduction planning efforts. In-depth observation, user journey mapping, early prototyping and additional approaches can be employed, as described in the examples above to gain a more comprehensive understanding of the end-user, her environment and the systems with which she interacts. Leveraging such methods will help the field better identify and create opportunities to target users and their key influencers with microbicide messages and positioning that will maximize uptake and adherence.

## References

[1] Baeten JM, Donnell D, Ndase P, Mugo NR, Campbell JD, Wangisi J (2012). Antiretroviral prophylaxis for HIV prevention in heterosexual men and women. N Engl J Med.

[2] Berger RE (2011). Effectiveness and safety of tenofovir gel, an antiretroviral microbicide, for the prevention of HIV infection in women. J Urol.

[3] Grant RM, Lama JR, Anderson PL, McMahan V, Liu AY, Vargas L (2010). Preexposure chemoprophylaxis for HIV prevention in men who have sex with men. N Engl J Med.

[4] Karim SSA, Kashuba AD, Werner L, Karim QA (2011). Drug concentrations after topical and oral antiretroviral pre-exposure prophylaxis: implications for HIV prevention in women. Lancet.

[5] Thigpen MC, Kebaabetswe PM, Paxton LA, Smith DK, Rose CE, Segolodi TM (2012). Antiretroviral preexposure prophylaxis for heterosexual HIV transmission in Botswana. N Engl J Med.

[6] Van Damme L, Corneli A, Ahmed K, Agot K, Lombaard J, Kapiga S (2012). Preexposure prophylaxis for HIV infection among African women. N Engl J Med.

[7] Marrazzo J, Ramjee G, Nair G, Palanee T, Mkhize B, Nakabiito Taljaard M (2013). Pre-exposure prophylaxis for HIV in women: daily oral tenofovir, oral tenofovir/emtricitabine or vaginal tenofovir gel in the VOICE study (MTN 003).

[8] Braunstein S, van de Wijgert J (2005). Preferences and practices related to vaginal lubrication: implications for microbicide acceptability and clinical testing. J Womens Health (Larchmt).

[9] Koblin BA, Andrasik M, Austin J (2013). Preparing for the unexpected: the pivotal role of social and behavioral sciences in trials of biomedical HIV prevention interventions. J Acquir Immune Defic Syndr.

[10] Rausch DM, Grossman CI, Erbelding EJ (2013). Integrating behavioral and biomedical research in HIV interventions: challenges and opportunities. J Acquir Immune Defic Syndr.

[11] Stirratt MJ, Gordon CM (2008). Adherence to biomedical HIV prevention methods: considerations drawn from HIV treatment adherence research. Curr HIV/AIDS Rep.

[12] Tolley EE, Severy LJ (2006). Integrating behavioral and social science research into microbicide clinical trials: challenges and opportunities. Am J Public Health.

[13] Terris-Prestholt F, Hanson K, MacPhail C, Vickerman P, Rees H, Watts C (2013). How much demand for new HIV prevention technologies can we really expect? Results from a discrete choice experiment in South Africa. PLoS One.

[14] Bentley ME, Morrow KM, Fullem A, Chesney MA, Horton SD, Rosenberg Z (2000). Acceptability of a novel vaginal microbicide during a safety trial among low-risk women. Fam Plan Perspect.

[15] Bentley ME, Fullem AM, Tolley EE, Kelly CW, Jogelkar N, Srirak N (2004). Acceptability of a microbicide among women and their partners in a 4-country phase I trial. Am J Public Health.

[16] Greene E, Batona G, Hallad J, Johnson S, Neema S, Tolley EE (2010). Acceptability and adherence of a candidate microbicide gel among high-risk women in Africa and India. Cult Health Sex.

[17] Morrow K, Rosen R, Richter L, Emans A, Forbes A, Day J (2003). The acceptability of an investigational vaginal microbicide, PRO Gel, among women in a phase I clinical trial. J Women’s Health (2002).

[18] Reiff M, Wade C, Chao MT, Kronenberg F, Cushman LF (2008). Health practices and vaginal microbicide acceptability among urban black women. J Womens Health (Larchmt).

[19] Rosen RK, Morrow KM, Carballo-Diéguez A, Mantell JE, Hoffman S, Gai F (2008). Acceptability of tenofovir gel as a vaginal microbicide among women in a phase I trial: a mixed-methods study. J Women’s Health (2002).

[20] Weeks MR, Mosack KE, Abbott M, Sylla LN, Valdes B, Prince M (2004). Microbicide acceptability among high-risk urban U.S. women: experiences and perceptions of sexually transmitted HIV prevention. Sex Transm Dis.

[21] Mngadi K, Maarschalk S, Grobler A, Mansoor L, Frohlich J, Madlala B (2014). Disclosure of microbicide gel use to sexual partners: influence on adherence in the CAPRISA 004 trial. AIDS Behav.

[22] Mansoor L, Karim Q, Werner L, Madlala B, Ngcobo N, Cornman D (2014). Impact of an adherence intervention on the effectiveness of tenofovir gel in the CAPRISA 004 trial. AIDS Behav.

[23] Morrow KM, Fava JL, Rosen RK, Vargas S, Shaw JG, Kojic EM (2013). Designing preclinical perceptibility measures to evaluate topical vaginal gel formulations: relating user perceptions and experiences to formulation properties. AIDS Res Hum Retroviruses [Internet].

[24] Van den Berg JJ, Rosen RK, Bregman DE, Thompson LA, Jensen KM, Kiser PF (2014). “Set it and forget it”: women's perceptions and opinions of long-acting topical vaginal gels. AIDS Behav.

[25] Gafos M, Mzimela M, Sukazi S, Pool R, Montgomery C, Elford J (2010). Intravaginal insertion in KwaZulu-Natal: sexual practices and preferences in the context of microbicide gel use. Cult Health Sex.

[26] Hall P (2011). IDEO takes on the government. Metropolis.

[27] Jahnke M (2009). Innovation through design thinking. Business.

[28] Brown T, Wyatt J (2010). Design thinking for social innovation. Stanford Soc Innovat Rev.

[29] Brown T (2008). Design thinking. Harv Bus Rev.

[30] Buchenau M, Francisco IS, Suri JF Experience prototyping.

[31] Whatmore J (2001). The art of innovation: success through innovation the IDEO way. Long Range Plan.

[32] Suri JF, Howard SG (2006). Going deeper, seeing further: enhancing ethnographic interpretations to reveal more meaningful opportunities for design. J Advert Res.

[33] Madsbjerb C, Rasmussen M (2014). The moment of clarity: using the human sciences to solve your toughest business problems.

[34] Sigall H, Aronson E, Van Hoose T (1970). The cooperative subject: Myth or reality?. J Exp Soc Psychol.

[35] Wang C, Burris MA (1997). Photovoice: concept, methodology, and use for participatory needs assessment. Health Educ Behav.

[36] Wang CC (1999). Photovoice: a participatory action research strategy applied to women's health. J Womens Heal Off Publ Soc Adv Womens Heal Res.

[37] Irwin R (2002). IDEO's design cure: can it fix our sick health-care system?. Metropolis.

[38] Shah A, Thathachari J, Agarwal R, Karamchandani A (2013). A market led, evidence based, approach to rural sanitation [Internet].

# Sub article: WHO guidance grounded in a comprehensive approach to sexual and reproductive health and human rights: topical pre-exposure prophylaxis

**DOI:** 10.7448/IAS.17.3.19279

**Published:** 2014-09-08

**Authors:** Manjula Lusti-Narasimhan, Rajat Khosla, Rachel Baggaley, Marleen Temmerman, Elizabeth McGrory, Tim Farley

**Affiliations:** 1 Department of Reproductive Health and Research, World Health Organization, Geneva, Switzerland; 2 Department of HIV/AIDS, World Health Organization, Geneva, Switzerland; 3 Consultant, New York, NY, USA; 4 Sigma 3 Services SÀRL, Nyon, Switzerland

**Keywords:** human rights, sexual and reproductive health, HIV, prevention, pre-exposure prophylaxis, microbicide

## Abstract

**Introduction:**

Two new microbicide products based on topical (vaginal) application of antiretroviral drugs – 1% tenofovir gel and the dapivirine ring – are currently in late-stage clinical testing, and results on their safety and effectiveness are expected to become available in early 2015. WHO guidelines on the use of topical pre-exposure prophylaxis (topical PrEP) are important in order to ensure that these new prevention products are optimally used.

**Discussion:**

Given that these new topical PrEP products are designed to be woman initiated and will likely be delivered in reproductive health settings, it is important to ensure that the guidance be framed in the context of comprehensive sexual and reproductive health and human rights. In addition to the safety and effectiveness data resulting from clinical trials, and the regulatory approval required for new products, the WHO normative guidelines on the use of topical PrEP will be essential for rapid roll-out in countries.

**Conclusions:**

Human rights standards and principles provide a framework for the provision of woman-initiated HIV prevention products. These include addressing issues related to the gender inequities which are linked to the provision of HIV-prevention, treatment and care for young girls and women. Effective programming for women and girls must therefore be based on understanding the local, social and community contexts of the AIDS epidemic in the country, and adapting HIV strategies and programmes accordingly. Such a framework therefore is needed not only to ensure optimal uptake of these new products by women and girls but also to address sociocultural barriers to women's and girls’ access to these products.

## Introduction

Two new microbicide products based on topical (vaginal) application of antiretroviral drugs for HIV prevention – 1% tenofovir gel and the dapivirine intravaginal ring – are currently in late-stage clinical testing and results on their safety and efficacy are expected to become available in early 2015.

To ensure that these products are able to reach women and young girls most at risk of HIV acquisition, their launch must be accompanied by WHO guidelines on the use of topical pre-exposure prophylaxis (topical PrEP). These guidelines must be evidence-based and integrate human rights standards and principles with recommendations taking into account policy, programmatic and community considerations. These guidelines are important to ensure that new topical PrEP products are implemented in high HIV incidence countries and settings in a manner that is equitable and effective. WHO recommendations have a strong influence on policy, can accelerate implementation in resource-limited settings and are necessary for accessing funding from some donors. They contain clinical, public health and policy recommendations about health interventions, and provide information about what policy makers, health-care providers or patients should do [1]. WHO has adopted internationally recognized standards and methods for guideline development [2] to ensure that guidelines are unbiased and that the products or interventions meet a public health need. The recommendations are based on a comprehensive and objective assessment of the available evidence and the process used to develop the recommendations is clearly described. WHO guidelines are complementary to regulatory review and approval. While national regulatory authorities have the responsibility to determine whether a product should be allowed onto the market in their jurisdiction, WHO guidelines provide advice for national programme managers and policy makers as they decide whether and how a product should be used. This advice addresses, for example, whether and how it should be prioritized to certain segments of the population or risk groups, and how to deliver the new product in an efficient and cost-effective manner with due consideration to other priority health interventions.

Given that new topical PrEP products are designed to be initiated by the woman herself and will likely be primarily delivered in reproductive health settings, it is important to ensure that national programmes are designed and services delivered within a framework of comprehensive sexual and reproductive health and human rights. Human rights standards, principles and treaties provide guarantees specifically relating to access to contraceptives. In addition, they recommend, among other actions, that states should ensure timely and affordable access to good quality sexual and reproductive health information and services, including contraception, which should be delivered in a way that ensures fully informed decision-making, respects dignity, autonomy, privacy and confidentiality, and is sensitive to individuals’ needs and perspectives. These guarantees are essential prerequisites to ensure women and girls are able to access and use the new HIV prevention methods.

WHO has recently issued guidance on ensuring that human rights are integrated into the provision of contraceptive information and services with a total of 24 recommendations on how this can be achieved [3]. Promotion and protection of human rights of women and girls are at the centre of this approach, including their right to have control over and decide freely and responsibly on matters related to their sexual and reproductive health, free of coercion, discrimination and violence. This requires governments to adopt and accelerate the implementation of laws, policies and programmes which protect and enable the enjoyment of all human rights and fundamental freedoms, including their reproductive rights in accordance with the International Conference on Population and Development (ICPD) Programme of Action [4], the Beijing Declaration and Platform for Action [5] and the 2004 WHO Global Reproductive Health Strategy [6]. Human rights are guaranteed in international and regional treaties, as well as in national constitutions and laws. They include the right to non-discrimination; the right to life, survival and development; the right to the highest attainable standard of health; and the rights to education and to information.

These rights have been applied by international, regional and national authoritative human rights bodies – such as UN treaty-monitoring bodies, international and regional courts, and national courts – to a wide range of sexual and reproductive health issues, including HIV, sexually transmitted infections (STIs), and recently to the accessibility of contraceptive information and services. This approach also resonates with global health programmes such as the PEPFAR Gender Strategy which recognizes that gender inequalities increase women's and girls’ vulnerability to HIV and must be addressed in designing and implementing HIV programmes, in particular the HIV prevention programmes [7]. The investment to develop new woman-initiated methods for HIV prevention has been driven by the recognition that there remain important gaps in HIV prevention, particularly for young women in generalized HIV epidemic settings, who remain at high risk of infection, even in clinical trial settings with intensive HIV risk reduction interventions. This is well illustrated by the HIV incidence among women in the placebo arms of recent randomized controlled trials of novel HIV prevention methods – 5.0 per 100 person years in the FEM-PrEP trial of oral PrEP trial conducted in Kenya, South Africa and Tanzania [8], 5.7 in the VOICE trial of oral and vaginal products conducted in South Africa and Uganda [9], 5.9 in the Phambili vaccine trial in South Africa [10] and 9.1 in the CAPRISA 004 trial of 1% tenofovir gel [11]. While condoms are highly effective in reducing the risk of HIV infection in serodiscordant couples [12] and among sex workers [13], their use and impact remains stubbornly low in sex between regular partners [14, 15].

## Discussion

A significant challenge to designing and implementing programmes to deliver new HIV prevention methods is to ensure that they are made available to, and used by, women at high risk of HIV infection who are not using existing methods. Many such women visit family planning and other reproductive health services to obtain contraceptive advice and supplies, or to seek treatment for STIs. Thus, these family planning and reproductive health services have a key role to play in providing information on the new HIV prevention methods, even if a woman can only obtain the products from a limited number of facilities. Making the products available to women who do not access family planning or other reproductive health services will be a greater challenge and innovative programmatic approaches must be developed.

Building upon existing human rights standards and principles, several issues are particularly relevant in the context of provision of woman-initiated HIV prevention products. These include, but are not limited to, improving quality of services through:

1. integration of and/or linkages between reproductive health and HIV services which provide topical HIV prevention, condoms and contraceptive commodities, supplies and equipment, covering a range of methods, including emergency contraception.
2. providing evidence-based, comprehensive information, education and counselling to ensure informed choice with regard to contraception, and to prevention and care for HIV and other STIs.

The ICPD Programme of Action recognizes that women and adolescent girls, especially those who live in impoverished and otherwise disadvantaged communities and circumstances, disproportionately bear the greatest costs and consequences of failure to promote and protect sexual and reproductive health and reproductive rights. However, many women who would benefit most from these new HIV prevention products are young women and adolescents who, in many communities, have poor access to services. They may live in contexts where health-care providers do not have the training or the means to provide young people with age-appropriate services, or choose not to do so based on their own biases. Legal and policy barriers, such as parental consent requirements, can also inhibit girls’ access to services. Issues of confidentiality, women's empowerment and participation and accountability are essential to ensure promotion and protection of sexual and reproductive health and human rights of women and girls. For instance, women's privacy, including confidentiality of medical and other personal information, needs to be respected throughout the provision of HIV and STI prevention and care, and contraceptive information and services. For some women, using, or trying to use, one of the new HIV prevention products may expose them to violence, or exacerbate the risk of intimate partner violence. The ICPD Programme of Action also obliges governments to ensure availability of and access to the information, comprehensive sexuality education, and quality sexual and reproductive health services necessary to ensure sexual and reproductive health and enjoy human rights.

Results from the CAPRISA 004 trial announced in 2010 showed that 1% tenofovir gel used around the time of intercourse reduced the risk of HIV infection by 39% among women in KwaZulu-Natal [11] and the FACTS001 study of the same product, currently underway in eight sites in South Africa, is expected to announce preliminary results in early 2015 [16]. This is in contrast to the VOICE trial of daily gel use which showed no reduction in HIV incidence when users were instructed to use the product every day irrespective of anticipated or actual sexual intercourse [9]. The failure to show any protective effect was attributed to poor adherence to daily product use. The product in a coitally dependent regimen is not likely to be licensed for use for at least two years, given the time necessary to collate data, prepare the regulatory dossier, submit to national regulatory authorities and allow in-depth regulatory review. It will be a further one or two years before the product becomes more widely available to women at high risk of HIV infection who choose to use it. A similar process and timeframe is expected for the second topical microbicide product in development, a vaginal ring containing the antiretroviral dapivirine which is currently in two parallel Phase 3 trials in Malawi, South Africa, Uganda and Zimbabwe [17, 18]. The intervening time from the end of Phase 3 trials to product availability provides an opportunity to implement operational research among former trial participants, new users and/or communities that have not been previously involved in the research. A carefully designed programme of operational research will inform service delivery approaches to best make the products available and support women in their use.

In contrast, there was no opportunity to implement operational research and learn how to design access programmes before oral PrEP was licensed in the United States in 2012. In 2010, the iPrEx study showed that daily oral tenofovir disoproxil fumarate and emtricitabine (TDF/FTC) reduced the risk of HIV infection by 44% among men and transgender women who have sex with men [19]. Additional studies published in 2012 showed that TDF alone or TDF/FTC was also effective in reducing HIV infection in heterosexual men and women in known serodiscordant couples, injecting drug users and other high HIV risk men and women [20–22]. The combination TDF/FTC was approved for use in HIV prevention by the US Food and Drug Administration and WHO released programme guidance in July 2012 with a provisional recommendation on use within the context of demonstration projects for serodiscordant couples, and men and transgender women who have sex with men [23]. The absence of programmatic experience or implementation research on how to deliver oral PrEP to users prevented more specific recommendations from being made at that time. A wide array of introductory and demonstration projects on oral PrEP are now being developed and implemented. These demonstration projects will provide important information over the next two years on acceptable and sustainable programme design, how to reach men and women at risk of HIV infection who wish to access and use oral PrEP and how to retain them in a comprehensive combination HIV prevention programme. However, the majority of the ongoing or planned demonstration projects are in serodiscordant couples, sex workers and men or transgender women who have sex with men. Only 2 of the 26 oral PrEP demonstration projects listed by AVAC in December 2013 involve women at risk of HIV infection who are neither sex workers nor in a stable, known serodiscordant relationship [24]. It is these women at risk of HIV infection who are likely to pioneer use of, and benefit most from, topical PrEP.

In March 2014, WHO and CAPRISA convened a stakeholder consultation to identify priority implementation research on tenofovir gel and dapivirine ring to inform development of WHO guidelines on the use of topical PrEP [25]. The consultation brought together a range of stakeholders to determine what issues were most critical for WHO guideline development, and the research approaches and timing where they can be addressed. Building on research questions and information gaps identified at previous consultations convened since the release of the CAPRISA 004 results, consultation participants prioritized key operational and policy research priorities for the provision of topical PrEP (Table 1).

**Table 1:** Key operational and policy research priorities for provision of topical PrEP

Priority area	Research elements
**Define the core service package** to deliver tenofovir gel and dapivirine ring safely and appropriately in different service delivery settings (family planning clinics, HIV testing sites, sexual and reproductive health services)	• HIV testing and retesting models (including frequency, location and self-testing or provider-led testing) • Prescribing and resupply models (location of initial supply and refills, frequency of product dispensing) • Counselling approaches • Whether and how to monitor for drug resistance in newly infected product users • Frequency and methods for measuring adherence • Barriers to use (travel, time in clinic)
**Determine how best to provide topical PrEP to adolescents and young women** (aged 15 to 24)	• Clinical safety studies in women below age 18 years to allow labelling for use in this age group • Feasibility and acceptability of delivering tenofovir gel and dapivirine rings to young women • Willingness and ability to use the product • How to offer topical PrEP without undermining condom use • Young women's beliefs about the products, risks and how these might affect adherence • Site-specific situation analyses to identify where young women go, or would go, to access topical PrEP for HIV prevention
**Support consistent product use**	• Tools to determine which factors influence use, and identify reasons for non-use • Examine factors at multiple levels that influence product use, and can be complemented by objective measures of adherence • Develop approaches that allow support to be tailored to the individual user • Ongoing assessment of adherence to products known to be safe and effective
**Develop provider training materials**	• Develop, test and adapt materials as experience in implementing programmes and supporting users accumulate • Draw on clinical trial documents and evidence as well as available training materials from other sexual and reproductive health and HIV prevention technologies • Practical tools for screening potential users to be used by providers and women to help identify those at high risk of HIV, those likely to adhere to product use, and other approaches for defining users
**Conduct policy analysis and synthesis to build an enabling environment**	• Synthesize evidence, outstanding questions and approaches to monitoring and minimizing resistance to address policymaker concerns • Synthesize evidence, outstanding questions and approaches to monitoring and minimizing risk compensation to address policymaker concerns • Identify the best strategies and approaches to demand creation • Determine how best to anticipate and address social issues (partner testing and disclosure, preventing and addressing intimate partner violence, considerations for sex workers) • Identify needed data to inform consistent approach and parameters for modeling cost effectiveness, impact and other key outcomes

In addition, stakeholders at the consultation noted that programme implementation of tenofovir gel and dapivirine ring could be addressed in each of the three phases of research: 1) open label extensions of the ongoing clinical trials; 2) pilot introductory studies and operational research after confirmation of safety and efficacy, but before product licensure; and 3) programmatic research following product licensure (Table 2). The stakeholder consultation strongly reaffirmed that implementation research and roll-out should be prioritized in communities and countries that have hosted clinical trials of topical PrEP and other HIV prevention trials in women, including full support for open label extension studies in the sites where trials of tenofovir gel and dapivirine ring are ongoing.

**Table 2:** Research phases and characteristics of operations research studies

Phase of research	Settings/key characteristics	Key outcomes
• Before product licensure Open label extension of efficacy trials	Efficacy trial sites Participation limited to efficacy trial participants All are former trial participants and successful prior users	Additional safety data to support licensure Key questions to allow less restrictive labeling: e.g. • Frequency of HIV testing, product resupply • Adherence support models • Alternative service delivery settings
Pilot introductory studies and operations research (following safety and effectiveness, prior to licensure)	New users Can be in trial sites or other settings Research requires ethical and regulatory approvals and individual informed consent	Inform guidance and product labeling Inform initial programme design (e.g. minimum service package, efficient adherence support models, feasible and sustainable service delivery models) Extend to new user groups (e.g. extended age range, relaxed eligibility criteria and extension to users previously excluded due to minor contraindications)
• After product licensure Demonstration projects/implementation research	New users Projects developed to inform programme design Research designed and conducted in the context of ongoing programmes	Inform successful programme scale up and adaptation Inform development of updated guidance as experience with product delivery and use accumulates Address public health use of products

## Conclusions

Given the continued high rate of new infections among women, especially young women in generalized HIV epidemics, it is critical to determine how best to ensure that effective HIV prevention products, including topical PrEP, can be delivered appropriately and sustainably. Given the gender inequities that drive the need for women-initiated products, WHO guidance on topical PrEP must not only build upon relevant existing WHO guidelines but also be grounded in a comprehensive sexual and reproductive health and human rights framework. Such guidance will be highly influential in determining how these products are provided, accelerating programme uptake of new technologies in resource-limited settings, and facilitating purchase and programme support by international agencies and donors.

Timely design and implementation of a programme of introductory and demonstration studies on topical PrEP will ensure that the period between confirmation of safety and efficacy and product licensure is best used. This period can generate new knowledge on how to deliver the products to users in an effective and sustainable manner, how to retain users in prevention programmes, what mix of HIV prevention methods can be effectively and efficiently delivered together, how to integrate with existing sexual and reproductive health programmes and how to involve the community and potential users in programme design, implementation and monitoring.

## References

[1] World Health Organization (2012). WHO handbook for guideline development.

[2] Guyatt GH, Oxman AD, Vist GE, Kunz R, Falck-Ytter Y, Alonso-Coello P (2008). GRADE: an emerging consensus on rating quality of evidence and strength of recommendations. Br Med J.

[3] World Health Organization (2014). Ensuring human rights in the provision of contraceptive information and services: guidance and recommendations.

[4] Plan of Action of the International Conference on Population and Development [Internet] (1994). http://www.un.org/popin/icpd/conference/offeng/poa.html.

[5] Fourth World Conference on Women (1995). Beijing Declaration and Platform for Action [Internet].

[6] World Health Organization (2004). Reproductive health strategy to accelerate progress towards the attainment of international development goals and targets. Report No.: WHO/RHR/04.8.

[7] The President's Emergency Plan for AIDS Relief (2013). Updated gender strategy [Internet].

[8] Van Damme L, Corneli A, Ahmed K, Agot K, Lombaard J, Kapiga S (2012). Preexposure prophylaxis for HIV infection among African women. N Engl J Med.

[9] Marrazzo J, Ramjee G, Nair G, Palanee T, Mkhize B, Nakabiito C Pre-exposure prophylaxis for HIV in women: daily oral tenofovir, oral tenofovir/emtricitabine, or vaginal tenofovir gel in the VOICE study (MTN 003) (Paper #26LB).

[10] Gray GE, Allen M, Moodie Z, Churchyard G, Bekker L-G, Nchabeleng M (2011). Safety and efficacy of the HVTN 503/Phambili study of a clade-B-based HIV-1 vaccine in South Africa: a double-blind, randomised, placebo-controlled test-of-concept phase 2b study. Lancet Infect Dis.

[11] Abdool Karim Q, Abdool Karim SS, Frohlich JA, Grobler AC, Baxter C, Mansoor LE (2010). Effectiveness and safety of tenofovir gel, an antiretroviral microbicide, for the prevention of HIV infection in women. Science.

[12] Weller SC, Davis-Beaty K (2002). Condom effectiveness in reducing heterosexual HIV transmission. Cochrane Database of Syst Rev.

[13] Rojanapithayakorn W (2006). The 100% condom use programme in Asia. Reprod Health Matters.

[14] Foss AM, Watts CH, Vickerman P, Heise L (2004). Condoms and prevention of HIV. Br Med J.

[15] Cleland J, Ali MM, Shah I (2006). Trends in protective behaviour among single vs. married young women in sub-Saharan Africa: the big picture. Reprod Health Matters.

[16] FACTS Consortium (2013). FACTS001 study [Internet].

[17] International Partnership for Microbicides Dapivirine microbicide ring.

[18] Microbicide Trials Network (2012). Phase III trial of dapivirine ring begins in Africa: ASPIRE testing new HIV prevention approach for women [Internet].

[19] Grant RM, Lama JR, Anderson PL, McMahan V, Liu AY, Vargas L (2010). Preexposure chemoprophylaxis for HIV prevention in men who have sex with men. N Engl J Med.

[20] Baeten JM, Donnell D, Ndase P, Mugo NR, Campbell JD, Wangisi J (2012). Antiretroviral prophylaxis for HIV prevention in heterosexual men and women. N Engl J Med.

[21] Choopanya K, Martin M, Suntharasamai P, Sangkum U, Mock PA, Leethochawalit M (2013). Antiretroviral prophylaxis for HIV infection in injecting drug users in Bangkok, Thailand (the Bangkok Tenofovir Study): a randomised, double-blind, placebo-controlled phase 3 trial. Lancet.

[22] Thigpen MC, Kebaabetswe PM, Paxton LA, Smith DK, Rose CE, Segolodi TM (2012). Antiretroviral preexposure prophylaxis for heterosexual HIV transmission in Botswana. N Engl J Med.

[23] WHO (2012). Guidance on oral pre-exposure prophylaxis (PrEP) for serodiscordant couples, men and transgender women who have sex with men at high risk of HIV [Internet].

[24] AVAC (2013). Ongoing and planned PrEP trials and demonstration projects [Internet].

[25] World Health Organization (2014). Meeting report on stakeholder consultation on priority implementation research to inform development of WHO normative guidance on topical pre-exposure prophylaxis.
